# Zengshengping improves lung cancer by regulating the intestinal barrier and intestinal microbiota

**DOI:** 10.3389/fphar.2023.1123819

**Published:** 2023-03-13

**Authors:** E. Sun, Xiangqi Meng, Zhaoxia Kang, Huimin Gu, Mingyu Li, Xiaobin Tan, Liang Feng, Xiaobin Jia

**Affiliations:** ^1^ Affiliated Hospital of Integrated Traditional Chinese and Western Medicine, Nanjing University of Chinese Medicine, Nanjing, China; ^2^ Key Laboratory of New Drug Delivery System of Chinese Meteria Medica, Jiangsu Provincial Academy of Chinese Medicine, Nanjing, China; ^3^ School of Traditional Chinese Pharmacy, China Pharmaceutical University, Nanjing, China

**Keywords:** Zengshengping, lung cancer, gut-lung axis, lewis, urethane, intestinal barrier, intestinal microbiota

## Abstract

Lung cancer is a common malignant tumor in clinical practice, and its morbidity and mortality are in the forefront of malignant tumors. Radiotherapy, chemotherapy, and surgical treatment play an important role in the treatment of lung cancer, however, radiotherapy has many complications and even causes partial loss of function, the recurrence rate after surgical resection is high, and the toxic and side effects of chemotherapy drugs are strong. Traditional Chinese medicine has played a huge role in the prognosis and improvement of lung cancer, among them, Zengshengping (ZSP) has the effect of preventing and treating lung cancer. Based on the “gut-lung axis” and from the perspective of “treating the lung from the intestine”, the purpose of this study was to research the effect of Zengshengping on the intestinal physical, biological, and immune barriers, and explore its role in the prevention and treatment of lung cancer. The Lewis lung cancer and urethane-induced lung cancer models were established in C57BL/6 mice. The tumor, spleen, and thymus were weighed, and the inhibition rate, splenic and thymus indexes analyzed. Inflammatory factors and immunological indexes were detected by enzyme-linked immunosorbent assay. Collecting lung and colon tissues, hematoxylin and eosin staining was performed on lung, colon tissues to observe histopathological damage. Immunohistochemistry and Western blotting were carried out to detect tight junction protein expression in colon tissues and expression of Ki67 and p53 proteins in tumor tissues. Finally, the feces of mice were collected to investigate the changes in intestinal microbiota using 16SrDNA high-throughput sequencing technology. ZSP significantly reduced tumor weight and increased the splenic and thymus indexes. It decreased expression of Ki67 protein and increased expression of p53 protein. Compared with Model group, ZSP group reduced the serum levels of interleukin (IL)-1β, IL-6, tumor necrosis factor α (TNF-α), and ZSP group increased the concentration of secretory immunoglobulin A (sIgA) in the colon and the bronchoalveolar lavage fluid (BALF). ZSPH significantly increased the level of tight junction proteins such as ZO-1, Occludin and Claudin-1. Model group significantly reduced the relative abundance of *Akkermansia* (*p* < 0.05) and significantly promoted the amount of *norank_f_*Muribaculaceae, *norank_f_*Lachnospiraceae (*p* < 0.05) compared with that in the Normal group. However, ZSP groups increased in probiotic strains (*Akkermansia*) and decreased in pathogens (*norank_f_*Muribaculaceae, *norank_f_*Lachnospiraceae). Compared with the urethane-induced lung cancer mice, the results showed that ZSP significantly increased the diversity and richness of the intestinal microbiota in the Lewis lung cancer mice. ZSP played an important role in the prevention and treatment of lung cancer by enhancing immunity, protecting the intestinal mucosa and regulating the intestinal microbiota.

## 1 Introduction

Lung cancer is one of the most common malignant tumors, with high morbidity and mortality globally (Thakur et al., 2020). Cancer has become the second leading cause of disease and the main cause of death among all malignant tumors, according to the data of the International Agency for Research on Cancer, affiliated to the World Health Organization (Sung et al., 2021). There are two main subtypes of lung cancer: small cell lung cancer and non-small cell lung cancer (NSCLC), of which NSCLC accounts for 85% of cases (Li et al., 2019). At present, the treatment regimen of NSCLC mainly comprises surgery, radiotherapy, and chemotherapy. The combination of surgery, chemotherapy and radiotherapy has made progress in the treatment of lung cancer, but the risk of surgery is high, the toxicity and side effects of radiotherapy and chemotherapy are high, and the overall prognosis of patients is poor (Rahal et al., 2017; Ye et al., 2017). Therefore, one of an important challenge for the clinical medical community and health departments in many countries is prevention and control of lung cancer. At present, there are many advocates for the use of traditional Chinese medicine (TCM) in the treatment of lung cancer, which could lead to the development of further lung cancer treatments in the future (Li et al., 2022). The main purpose of western medicine is to treat tumors, while the main purpose of traditional Chinese medicine is to treat patients as a whole to enhance their immunity (Zhang et al., 2021). The combination of the two strategies could potentially achieve the best outcome in the treatment of lung cancer.

Zengshengping (ZSP) is a widely used antitumor TCM compound in clinics. It is a Chinese herbal medicine formulation consisting of *Sophora tonkinensis* Gagnep, *Polygonum bistorta* L, *Prunella vulgaris* L, *Sonchus brachyotus* L, *Dictamnus dasycarpus* Turcz and *Dioscorea bulbifera* L. ZSP was mainly used in the treatment of esophageal cancer in the early 1990s, and it was later confirmed that it had a good preventive effect on oral cancer (Wang et al., 2013). The Alvin J Siteman Cancer Center reported the research results of animal experiments on using ZSP in the prevention of lung cancer, and believed that ZSP could significantly reduce the tumor diversity and tumor load of lung cancer in mice, affect the expression of related genes in multiple cell signal transduction pathways (*K-ras-2, p53, Ink4a/Arf*), and inhibit gene mutation (Zhang et al., 2004). Later, the center reported a key study on the blocking effect of ZSP on precancerous lesions of lung squamous carcinoma in mice, and observed the effects of ZSP on the occurrence of lung hyperplasia, metaplasia, carcinoma *in situ* and invasive carcinoma during chemical induction, and the absorption and distribution of ZSP in mice (Wang et al., 2009). The results showed that ZSP was an effective chemical preventive agent for blocking precancerous lesions of lung squamous carcinoma in mice. The specific mechanism of the anticancer effect of ZSP requires further exploration. Studies have shown that the six components in ZSP all have an inhibitory effect on tumors. *Sophora tonkinensis* Gagnep has a certain inhibitory effect on human lung cancer A549 tumor cells (Pan et al., 2016). The chloroform and hexane fractions of *P. bistorta* L and their subcomponents have inhibitory effects on P338 (mouse lymphocytic leukemia), HL60 (human leukemia) and LL2 (Lewis lung cancer) cell lines (Manoharan et al., 2007). The results of modern pharmacological studies showed that both the aqueous extract and the alcohol extract of *P. vulgaris* L exhibited good antitumor activity (Feng et al., 2010a). Dictamnin A and Dictamnin B, compounds in *D. dasycarpus* Turcz extract, have a strong inhibitory effect on the proliferation of A549 cells, followed by Dictamine, and the activities of flavonoids, rutevin X, fraxinellonone, and limonin are weak (Yoon et al., 2010). The extract of *S. brachyotus* L has been shown to have an antitumor effect (Huyan et al., 2016) and the extract of the *D. bulbifera* L seed has obvious inhibitory effects on the growth of three human cancer cell lines (cervical cancer cell lines SiHa and HeLa, liver cancer cell line HepG2) (Chaniad et al., 2020; Zhao, 2009). The above research results show that *S. tonkinensis* Gagnep, *P. bistorta* L, *P. vulgaris* L, and *D. dasycarpus* Turcz have anti-lung cancer properties. Although there is no known literature that clearly shows that *S. brachyotus* L and *D. bulbifera* L have an effect on lung cancer, these two medicines have a certain inhibitory effect on colon and gastric cancer (Zheng and Sun, 2016; Ahmad et al., 2018; Feng et al., 2010b; Gong et al., 2021).

“The exterior-interior relationship between the lung and the large intestine” is a traditional theory of traditional Chinese medicine. It means that the lung in the five zang-organs and the large intestine in the six fu-organs are interior-exteriorly related and complement each other. The ancients’ understanding of “the exterior-interior relationship between the lung and the large intestine” includes three aspects: meridian, qi movement and fluid conduction. The current explanation of “the exterior-interior relationship between the lung and the large intestine” includes: histoembryology, intestinal endotoxin, intestinal mucosal immunity, and microecological environment. It is believed that there are a large number of normal microflora on the surface of respiratory tract and gastrointestinal mucosa, and lung disease and intestinal disease will lead to another change of microflora. The theory of " lung and large intestine being interior-exteriorly related " has been reported and applied in lung diseases such as cough, asthma, pulmonary fibrosis and intestinal diseases such as constipation and enteritis, but it is less involved in the treatment of lung cancer.

Intestine is a symbiotic system with a large number of bacteria and other microorganisms, which plays an important role in maintaining human health. It constitutes an extremely complex micro-ecosystem and maintains a relatively stable balance, affecting the immune and anti-inflammatory axis (Holmes et al., 2012; Nicholson et al., 2012; Tremaroli and Bäckhed, 2012). Accumulative evidence shows that changes in the composition (imbalance), function or interaction between intestinal flora and host are directly related to many diseases. For example, the study reported the relationship between cancer and intestinal microflora (Gui et al., 2015; Routy et al., 2018; Zhang et al., 2018a). In addition, Intestinal microbial function of cancer patients declined in general, and *Enterococcus* and *Bifidobacterium* are reported as potential biomarkers of cancer (Zhuang et al., 2019). Furthermore, it is reported that the levels of *Kluyvera*, *Escherichia-Shigella*, *Dialister*, *Faecalibacterium* and *Enterobacter* in cancer patients are lower, while the levels of *Veillonella*, *Fusobacterium* and *Bacteroides* are significantly higher than those in healthy individuals. (Zhang et al., 2018b). Therefore, for lung cancer, a gut-lung axis strategy was proposed to change the composition of intestinal microbiota and the application of the idea of the “gut-lung axis” theory is based on the interrelation between the lungs and the large intestine to achieve the purpose of “treating the lung from the intestine” (Yan et al., 2020).

In the Zengshengping tablets, *S. tonkinensis* Gagnep and *D. bulbifera* L return to the lung meridian, *S. brachyotus* L returns to the large intestine meridian, and *P. bistorta* L to both the lung meridian and the large intestine meridian. From the perspective of attribution, the treatment of lung cancer by proliferative tablets is in line with the theory of “the exterior-interior relationship between the lung and the large intestine”.

This study, based on the theory of “the exterior-interior relationship between the lung and the large intestine”, used short-term model (five groups: blank group (Normal), model group (Model), positive group (DDP), ZSP low-dose group (ZSPL) and ZSP high-dose group (ZSPH), with six mice in each group.) and long-term lung cancer model (four groups: blank group (Normal), model group (Model), ZSP low-dose group (ZSPL) and ZSP high-dose group (ZSPH), with twenty mice in each group.) to investigate the effects of ZSP on the intestinal physical, biological, and immune barriers in mice with lung cancer. We discussed the correlation between intestinal microbiota and cancer occurrence, and the effect of intestinal microbiota on cancer treatment response. In addition, we demonstrated the therapeutic effect of ZSP on lung cancer and expanded the scope of potential ZSP clinical application.

## 2 Materials and methods

### 2.1 Reagents and materials

*Sophora tonkinensis* Gagnep (batch number: 180801; origin of medicinal materials: Guangxi, China), *P. vulgaris* L (batch number: 180801; origin of medicinal materials: Henan, China), *S. brachyotus* L (batch number: 180801; origin of medicinal materials: Hebei, China), *D. dasycarpus* Turcz (batch number: 180801; origin of medicinal materials: Liaoning, China), and *D. bulbifera* L (batch number: 180801; origin of medicinal materials: Guangxi, China), were purchased from Anhui Huchuntang Traditional Chinese Medicine Slices Co., Ltd. *Polygonum bistorta* L (batch number: 171101C366; origin of medicinal materials: Liaoning, China) was purchased from Hebei Golden Leaf Pharmaceutical Co., Ltd. The above prepared pieces were identified by Prof. Wang Long of China Pharmaceutical University as meeting the requirements of “Medicinal Materials and Prepared Pieces” in the First part of Pharmacopoeia 2020 Edition.

Urethane (content ≥99%) was purchased from Shanghai Aladdin Biochemical Technology Co., Ltd. (batch number: 51-79-6, Shanghai, China). Cisplatin (DDP) was purchased from Qilu Pharmaceutical Co., Ltd. (batch number: AA4A8020A; specification: 20 mg/dose). Dulbecco’s Modified Eagle Medium was purchased from Jiangsu KGI Biotechnology Co., Ltd. (batch number: KG037381, Jiangsu, China). Ultrapure water was prepared using a Milli-Q water purifier (Merck Millipore, Germany). Fetal bovine serum was purchased from Zhejiang Tianhang Biotechnology Co., LTD. (batch number: 19010501, Zhejiang, China). Trypsin (0.25%) was purchased from Jiangsu KGI Biotechnology Co., LTD (batch number: KGY0012-100). Dimethyl sulfoxide (DMSO) was purchased from Jiangsu KGI Biotechnology Co., LTD. (batch number: ST038, Jiangsu, China). The secretory immunoglobulin A (sIgA) kit was purchased from Sengbega Biotechnology Co., LTD. (batch number: BI-WB005). Enzyme-linked immunosorbent assay (ELISA) kits for interleukin (IL)-6 (batch number: SU-B20012), IL-1β (batch number: SU-B20533), and tumor necrosis factor α (TNF-α) (batch number: SU-B20220) were purchased from Kenuodi Biotechnology Co., Ltd. *ß*-actin was purchased from Servicebio (batch number: GB12001). ZO-1 was purchased from Wuhan Sanying (batch number: 21773-1-AP). Occludin was purchased from BI OSS (batch number: BS-1495R). Claudin-1 was purchased from Wuhan Sanying (batch number:13050-1-AP). HRP-labeled goat anti-rabbit IgG was purchased from Servicebio (batch number: GB23303). HRP labeled donkey anti-goat IgG was purchased from Servicebio (batch number: GB23404). HRP-labeled goat anti-mouse IgG was purchased from Servicebio (batch number:GB23301). HRP-labeled goat anti-rat IgG was purchased from Servicebio (batch number:GB23302). Citric acid (PH6.0) antigen repair solution was purchased from Wuhan Google Biotechnology Co., Ltd. (batch number: G1202). PBS buffer was purchased from Wuhan Google Biotechnology Co., Ltd. (batch number: G0002). Hydrogen peroxide solution was purchased from Sinopharm Chemical Reagents Co., Ltd. (batch number: 10011218). BSA was purchased from Solarbio (batch number: A8020). The DNA extraction kit was purchased from Omega Bio-Tek (batch number: OMEGA soil). Cell line: Mouse Lewis lung cancer cell line, was provided by Associate Researcher Jie Song of Jiangsu Provincial Academy of Chinese Medicine.

The equipment used was as follows: CO_2_ cell incubator (Thermo Forma, United States), enzyme-linked immunoassay (Molecular Instruments, Inc., United States), microscope (CIC, United States), Ultrafine spectrophotometer (Thermo Fisher Scientific, United States), electrophoresis apparatus (Liuyi Instrument Factory, Beijing, China), polymerase chain reaction (PCR) instrument (ABI, United States), MISEQ sequencer (Miseq, United States).

#### 2.1.1 Drug preparation

**FIGURE 1:**
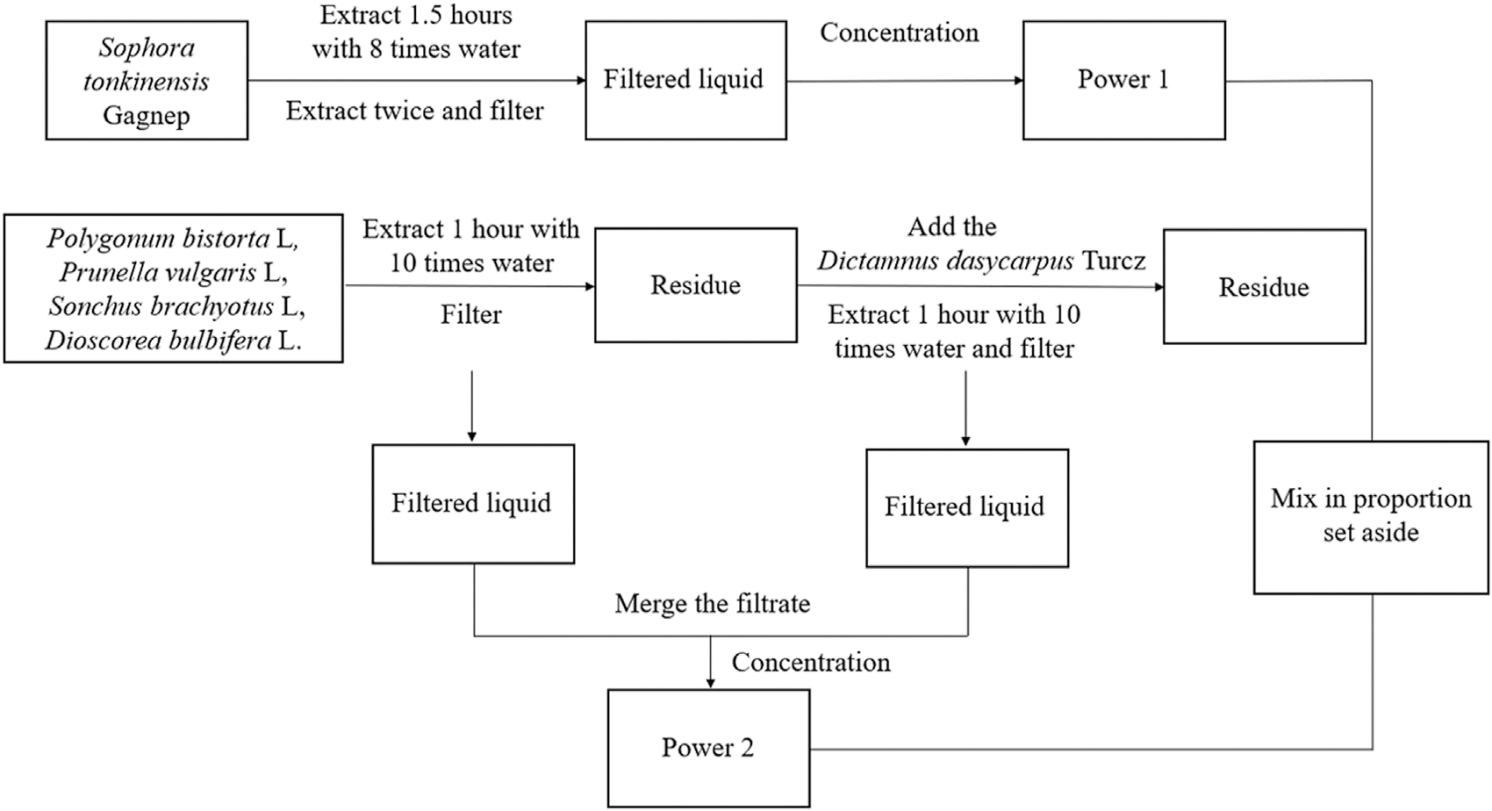
Extraction flow chart of Zengshengping.

The six herbal compounds, *S. tonkinensis* Gagnep, *P. bistorta* L, *P. vulgaris* L, *S. brachyotus* L, *D. dasycarpus* Turcz and *D. bulbifera* L were extracted at a ratio of 4.2:4.2:4.2:4.2:2.1:1 according to Figure 1. Powder one was obtained by weighing 147.0 g of *S. tonkinensis* Gagnep and the yield was 16.95%.

Powder two was obtained by weighing 147.0 g each of *P. bistorta* L, *P. vulgaris* L, and *S. brachyotus* L, 73.5 g of *D. dasycarpus* Turcz and 35.0 g of *D. bulbifera* L 35.0 g. The yield was 21.30%. All were stored in a dryer with the batch number of ZSP01.

DDP solution: 40 ml of 0.9% NaCl solution was added to a bottle of lyophilized DDP powder (containing 20 mg DDP) to make a 0.5 mg ml^-1^ solution (Dasari and Bernard, 2014; Ghosh, 2019).

ZSP solution: Ultrapure water was added to the powders to prepare the 35 g kg^-1^ (ZSPL) and 65 g kg^-1^ (ZSPH) concentrations of liquid medicine.

Urethane liquid: 1.04 g of urethane was added to ultrapure water to a total of 13 ml to prepare an 80 mg ml^-1^ of urethane solution (Imyanitov et al., 2020; Imyanitov et al., 2021; Sozio et al., 2021).

### 2.2 Animals and treatments

Experimental animals: C57BL/6 mice, male, weight 18–20 g, 6–8 weeks old, specific-pathogen-free grade, provided by the testing room of Beijing Weitong Lihua Experimental Animal Technology Co., Ltd. (License number: SCXK (Beijing) 2016-0002). Animal experiments were conducted to follow the guidelines of the Animal Care and Use Committee of Jiangsu Provincial Academy of Chinese Medicine (Animal ethics number: AEWC-20190306-74).

**Lewis lung cancer model:** The C57BL/6 mice were randomly divided into five groups. The mice were accommodated for 1 week, food and water were freely obtained under standard laboratory conditions (25°C ± 2°C, 45% ± 10% relative humidity, and 12 h:12 h light/dark cycle). Lewis lung cancer cells were cultured to logarithmic growth phase and washed twice with phosphate buffered saline (PBS). After cell digestion, the medium was added to prepare 1×10^7^ cells·ml^-1^ cell suspension. Except for the blank group, 0.2 ml cell suspension (containing 2*10^6^ cells) was subcutaneously injected into the right forelimb axilla of each mouse. The Normal group was the sham operation group, and normal saline was injected into the right forearm of the mice, 0.2 ml per mouse. The Normal group and the Model group were administered respectively 0.4 ml distilled water intragastrically. The ZSPL group and the ZSPH group were intragastric administered 0.4 ml ZSP solution per mouse. The DDP group was intraperitoneally injected with 0.2 ml DDP (0.5 mg ml^-1^), which was used on a regular basis and given every other day. The day of inoculation was recorded as day 0, and the administration began on day 1 and continued for 15 days.

**Urethane-induced lung cancer model:** Apart from the Normal group, the remaining groups were used to establish the urethane-induced lung cancer model by intraperitoneally injecting urethane solution 0.2 ml on Monday and Friday of each week for 5 weeks (Imyanitov et al., 2020; Imyanitov et al., 2021; Sozio et al., 2021). The Normal group was intraperitoneally injected with normal saline of equal volume for 5 weeks. The ZSPL and ZSPH groups were administered 0.4 ml of ZSP solution intragastrically each day, and the Normal and Model groups were intragastrically administered the same volume of distilled water each day for 17 weeks.

#### 2.2.1 Animal handling

Two hours following the final administration of ZSP, DDP or distilled water, the mice in each group were weighed, and the mice then euthanized. Blood, bronchoalveolar lavage fluid (BALF) and feces were collected and stored in the refrigerator at −80°C. In addition, the tumor tissue, spleen and thymus were completely exfoliated and weighed. The tumor inhibitory rate and spleen and thymus indexes calculated. Tumor tissues were stored in 10% formaldehyde after weighing, histopathological examination and immunohistochemical analysis were performed. The colon was removed and a section preserved in 10% formaldehyde. Histopathological examination and immunohistochemical analysis of ZO-1, Occludin and Claudin-1 were performed on some and some was stored in the refrigerator at −80°C, and the expression of tight junction protein was detected using Western blotting.

### 2.3 Effects on tumor inhibition rate and immune organs

The intact tumor was dissected, and the spleen and thymus were stripped and weighed. Tumor inhibition rate, splenic and thymus indexes were calculated.

### 2.4 Immunohistochemical analysis

The tumor was washed with PBS, fixed with 10% formaldehyde, embedded in conventional paraffin, sectioned, repaired with antigen, inactivated with 3% H_2_O_2_, dropped with 3% room temperature sealing, added with Ki67 and p53, ZO-1, Occludin, Claudin-1 antibodies, incubated overnight at 4°C, incubated with the second antibody at room temperature for 50 min, DAB chromogenic, re-stained nucleus, dehydrated and sealed. The images were collected under the light microscope for analysis (Yoshimura et al., 2019).

### 2.5 ELISA detection

The proinflammatory cytokines (IL-6, IL-1β and TNF-α) in the serum and the sIgA content in the BALF and colon of the mice were determined using the corresponding ELISA kits. Add 50 μl of microplate tag reagent to each well, seal the plate with a membrane and incubate at 37°C protected from light for 1 h. After the incubation, discard the reagent in the well of the enzyme label, spin dry, dilute and wash the plate with wash solution for three washes. Add 50 μl of reaction solution per well and incubate at 37°C protected from light for 30 min. Then add 50 μl of stop solution per well to determine the absorbance of each well at 450 nm.

### 2.6 Colonic damage evaluation

Hematoxylin and eosin (H&E) staining: The lungs and colon were washed with PBS, fixed in 10% neutral formaldehyde for 24 h, properly trimmed into small tissue blocks, and loaded into embedded boxes. The encapsulated cassette was dehydrated by concentration gradient alcohol, and the xylene transparent and paraffin wax was impregnated and sliced with a paraffin pathological microtome with a thickness of about 4 μm. The sections were subjected to conventional HE staining, paraffin sections were dewaxed, hematoxylin was stained for 5 min and rinsed naturally with running water, then acidified with 1% hydrochloric acid alcohol for 5 s and then washed back to blue with tap water, 1% eosin staining solution for 2 min, soaked in tap water for 30 s and then dehydrated, and finally sealed with neutral gum to observe the histopathological changes of lung and colon under light microscopy.

Periodic acid-Schiff staining (PAS): The iodate was dripped onto the slide and incubated at room temperature for 10 min, then washed with distilled water. After adding Schiff solution, incubated at room temperature for 20 min followed by hematoxylin stain and the slides observed under a light microscope.

### 2.7 Western blot analysis

Harvest the cells and rinse with pre-chilled PBS. Then, centrifuge with protein lysate (100 μg) for 15 min at 4°C. Samples of whole-cell protein lysates were electrophoresis on a 10% sodium dodecyl sulfate polyacrylamide gel and then electroblot transferred to a polyvinylidene fluoride (PVDF) membrane. Incubate the membrane with the corresponding primary antibody. Subsequently, the protein bands are incubated with HRP-bound secondary antibodies. Use Image Pro Plus (version 6.0) to quantify band strength.

### 2.8 16S rDNA high-throughput sequencing

The total DNA was extracted according to the instructions of the kit. NanoDrop2000 was used to detect the concentration and purity of DNA, and 1% agarose gel electrophoresis was used to detect the quality of DNA extraction. PCR amplification of the V3-V4 (ACT​CCT​ACG​GGA​GGC​AGC​AG, GGACTACHVGGGTWTCTAAT) variable region was performed with 338F (5′-ACT​CCT​ACG​GGA​GGC​AGC​AG-3′) and 806R (5′-GGACTACHVGGGTWTCTAAT-3′) primers. The amplification procedure was as follows: Predenaturation at 95°C for 3 min, 27 cycles (denaturation at 95°C for 30 s, annealing at 55°C for 30 s, extension at 72°C for 30 s), and finally extension at 72°C for 10 min. The amplification system was 20 μL: 4 μl 5*FastPfu buffer, 2 μl 2.5 mM dNTPs, 0.8 μl primer (5 μM), 0.4 μl FastPfu polymerase, 10 ng DNA template. PCR products were recovered using 2% agarose gel, purified by an AxyPrep DNA Gel Extraction Kit, eluted by Tris-HCl, and detected using 2% agarose gel electrophoresis. Quantification was performed using a QuantiFluor™-ST. According to the Illumina MiSeq standard operating procedure, a paired end 2*300 library was constructed using purified amplified fragments. Steps for library construction: 1) Connect the “Y" connector; 2) Use magnetic bead screening to remove the self-connecting section of the joint; 3) PCR amplification enrichment library template; 4) sodium hydroxide denaturation to produce single-stranded DNA fragments. Sequencing was performed using Illumina’s Miseq PE300 platform (Magi Biomedical Technology Co., LTD., Shanghai, China).

### 2.9 Statistical analysis

The data were expressed as the mean ± SD. The data obtained were statistically analyzed with SPSS 21.0 software (SPSS Inc., Chicago, United States). Values of *p* < 0.05 were considered statistically different.

## 3 Results

### 3.1 Therapeutic effect of ZSP on lung cancer based on the lewis short-term lung cancer model

#### 3.1.1 The effect of ZSP on tumor and its influence on immune organs

**FIGURE 2:**
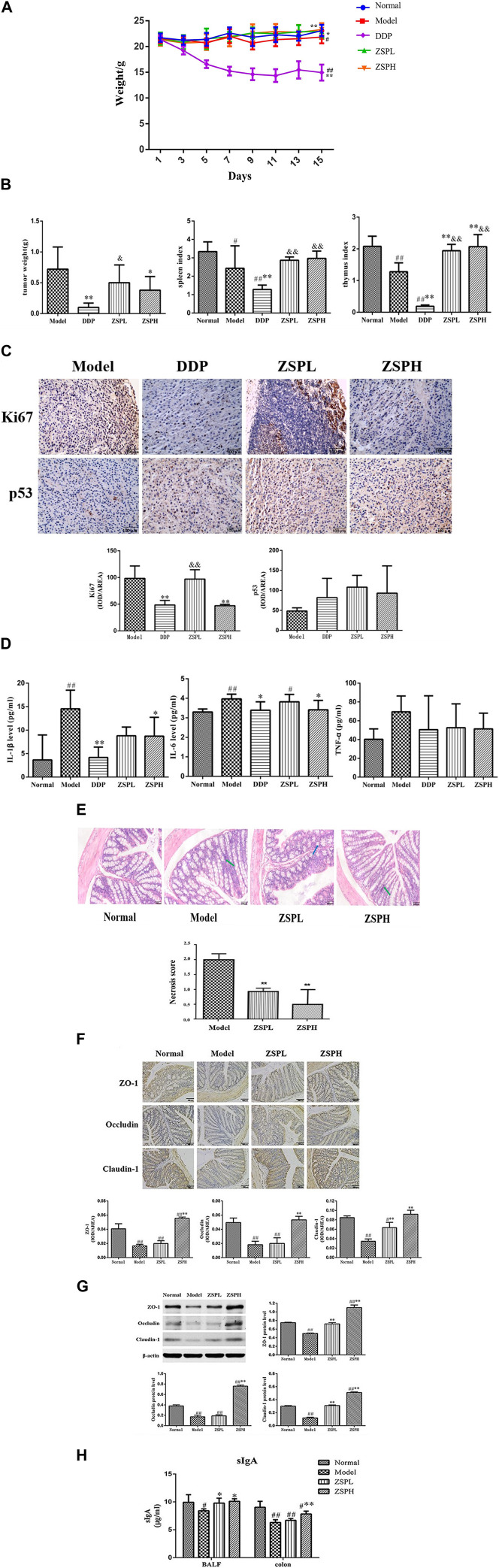
(Continued).

The weight changes of the mice in each group are shown in Figure 2A. On the 15th day, the weight of the mice in the Model group (*p* < 0.05) and DDP group (*p* < 0.01) showed a significantly decrease compared with those in the Normal group. Compared with the Model group, the DDP group decreased significantly (*p* < 0.01), while the ZSPL group (*p* < 0.05) and ZSPH group (*p* < 0.01) increased significantly.

As shown in Figure 2B, following intragastric administration of ZSP, the tumor growth of tumor-bearing mice decreased to different degrees. Compared with the Model group, the tumor weight in the DDP group was significantly reduced to 0.10 ± 0.07 g (*p* < 0.01), and the tumor inhibition rate was as high as 86.45%. The tumor weight of the ZSPL and ZSPH groups were reduced to 0.50 ± 0.29 g and 0.38 ± 0.22 g respectively (*p* < 0.05), and the tumor inhibition rates reached 30.88% and 47.66% respectively. The tumor weight was therefore reduced in the ZSPL group, however DDP and ZSPH significantly inhibited tumor growth (*p* < 0.01, *p* < 0.05). The tumor weight in the ZSPL and ZSPH groups were greater than in the DDP group with the ZSPL group significantly greater (*p* < 0.05). The splenic and thymus indexes of the Model group were significantly lower than the Normal group (*p* < 0.01, *p* < 0.05), indicating that the immune function of the tumor-bearing mice was decreased. The splenic and thymus indexes of the DDP group were also significantly lower than the Normal and Model groups (*p* < 0.01), indicating that DDP had inflicted significant damage to the immune function of the mice. Compared with the Model group, the spleen indexes of the ZSPL and ZSPH groups were increased, and the thymus indexes increased significantly (*p* < 0.01). Compared with the DDP group, the spleen and thymus indexes of the ZSPL and ZSPH groups were increased significantly (*p* < 0.01).

#### 3.1.2 Expression of Ki67 and p53 proteins in tumor tissues

Ki67 is a general marker of cell proliferation and p53 is a tumor suppressor protein that plays an important role in the process of tumor generation and growth, both widely used in clinical diagnosis (Lei et al., 2013). As shown in Figure 2C, compared with the Model group, the expression of Ki67 protein in the DDP and ZSPH groups was significantly downregulated (*p* < 0.01). Compared with the Model group, there was no significant difference in the expression of p53 protein in the ZSPL and ZSPH groups, but it was upregulated. The results indicate that ZSP had a good inhibitory effect on lung tumor growth.

#### 3.1.3 Levels of IL-1β, IL-6 and TNF-α in serum

As shown in Figure 2D, the levels of IL-1β, IL-6 and TNF-α in the Model group serum were higher than in the Normal group and IL-1β and IL-6 were significantly increased (*p* < 0.01). Compared with the Model group, the levels of IL-1β, IL-6 and TNF-α in the DDP group were decreased, of which the IL-1β and IL-6 were significantly decreased (*p* < 0.05, *p* < 0.01), and the IL-1β and IL-6 in the ZSPH group were significantly decreased (*p* < 0.05), and although TNF-α was not significantly decreased, ZSP showed a trend of regression. Compared with the DDP group, the expression levels of inflammatory factors in the ZSPL and ZSPH groups showed an upward trend, but there was no significant difference. These results indicate that ZSP can inhibit the expression of IL-1β, IL-6 and TNF-α, which regulate the inflammation of the body to a certain extent, to achieve the antitumor effect.

### 3.2 Effect of ZSP on the intestinal barrier

#### 3.2.1 The pathological changes of colon tissue

Under the light microscope, the pathological changes of the colon tissue in each group were observed in the Lewis model, as shown in Figure 2E. In the Normal group, the morphology and structure of the epithelial cells in the colonic mucosa layer were intact, the morphology and structure of the loose connective tissue in the submucosa were intact, and the structure of the muscularis and serosa layer were intact and clearly distributed. In the Model group, the morphological structure of colonic epithelial cells was intact, and some villus apical submucosal spaces appeared. In the ZSPL and ZSPH groups, the morphology and structure of colonic mucosal epithelial cells were intact and arranged neatly, the intercellular space was slightly widened, there was no obvious proliferation of cells and the structure of the muscularis and serosa layers were intact. The semi-quantitative score of the percentage of colon tissue necrosis after resection showed that the model group had the highest percentage (about 20%), while the ZSPH group had the lowest percentage (about 5%). Although the morphology and structure of the colon tissue of the four groups were different, the overall physiological state was similar, which pathological changes were not obvious.

#### 3.2.2 Expression of tight junction proteins

ZO-1 protein was positively expressed in the cytoplasmic region and cell membrane of intestinal mucosal epithelial cells in the Normal mice colons, Occludin protein was positively expressed in the cell membrane and the proximal side of the membrane, and Claudin-1 protein was positively expressed in the cell membrane of intestinal mucosal epithelial cells. Image-Pro Plus (version 6.0) software was used for semi-quantification, and the average optical density value was calculated. The results of protein expression detected using immunohistochemistry are shown in Figure 2F. Compared with the Normal group, expression of the tight junction proteins ZO-1, Occludin and Claudin-1 in the Model group were significantly decreased (*p* < 0.01). In addition, compared with the Model group, the expressions of ZO-1 and Occludin were significantly increased in the ZSPH group (*p* < 0.01). The expression of Claudin-1 was significantly increased in the ZSPL and ZSPH groups (*p* < 0.01).

Further analysis of expression of the three proteins was carried out using Western blotting, and the results are shown in Figure 2G. The expression of ZO-1, Occludin and Claudin-1 were decreased in the Model group compared with the Normal group. ZSP increased the expression of ZO-1, Occludin and Claudin-1 proteins, with significant effect in the ZSPH group, which was consistent with the immunohistochemistry results. It shows that lung disease can affect the intestine; lung cancer can reduce the protein expression of ZO-1, Occludin and Claudin-1 in the colon tissue of mice, and ZSP can inhibit this protein expression change in the colon tissue caused by the pathological transformation of the lung and intestine. ZSP has a protective effect on the intestinal mucosal physical barrier in mice with lung cancer.

#### 3.2.3 sIgA content in the BALF and colon

SIgA is the immunoglobulin most synthesized and secreted by the body. It is the common molecular basis and main executor of mucosal immunity. sIgA in human lung and intestinal tissues is specifically correlated, which is one of the important material bases reflecting “the exterior-interior relationship between the lung and the large intestine” (Zhang et al., 2018a). As shown in Figure 2H compared with the Normal group, the quantity of sIgA in the BALF of the Model group and the quantity of sIgA the colon of the Model, ZSPL and ZSPH groups were significantly decreased (*p* < 0.05, *p* < 0.01). Compared with the Model group, the content of sIgA in BALF of the ZSPL and ZSPH groups was significantly increased (*p* < 0.05), and the content of sIgA in the colon of the ZSPH group was significantly increased (*p* < 0.01). The trend of the BALF was consistent with that of the colon. The results showed that ZSP had a protective effect on the intestinal mucosal immune barrier of lung cancer mice.

#### 3.2.4 Alpha-diversity of intestinal microbiota and rarefaction curves

**FIGURE 3:**
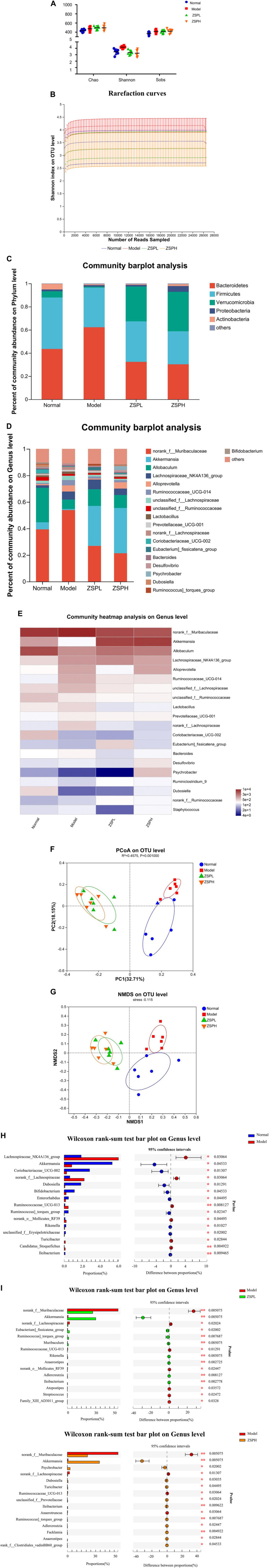
(Continued).

To examine whether ZSP regulates intestinal microbiota, 16S rDNA sequencing was performed on mouse fecal samples. Alpha-diversity is an ecological measure of how many taxonomic groups are present within each sample and whether such groups are evenly distributed (Cui et al., 2021). As shown in Figure 3A, Alpha-diversity includes the Chao, Shannon and Sobs indexes; species richness information is reflected by the Chao index, while species richnesas and evenness are reflected by the Shannon index, and the Sobs index refers to the number of operational taxonomic units (OTUs) actually observed (Li et al., 2022). The Chao index of the treatment groups were significantly higher than the Normal group, indicating that the abundance of intestinal bacteria in the ZSPL and ZSPH groups were higher than in the Normal group. The Shannon index of the Model group was higher than that of the Normal group, which may be related to the increase in pathogenic bacteria. The coverage index of the four groups were all greater than 0.997, indicating that the sequencing results could represent the genuine composition of intestinal bacteria in the samples. Rarefaction curves reflected that the depth of sequencing covered new rare phylotypes and the majority of the diversity (Cui et al., 2021). Some flattening rarefaction curves were obtained, which confirmed that the microbial diversity was mostly obtained (Figure 3B).

#### 3.2.5 Composition and abundance of intestinal microbiota

Intestinal microbiota structure changes are closely related to the occurrence and development of lung cancer. To determine the specific composition of the intestinal microbiota, we assessed microbial differences at different levels (phylum, genus). On the phylum level, as shown in Figure 3C, five major bacterial phyla were identified. *Firmicutes*, *Bacteroidetes*, and *Verrucomicrobia* were found to be predominant in all the samples. Compared with the Normal group, in the Model group, the presence of *Bacteroidetes* was significantly increased, whereas *Firmicutes* and *Verrucomicrobia* were significantly decreased. ZSPL and ZSPH significantly reduced the ratio of *Bacteroidetes*, the abundance of *Firmicutes* could be significantly reduced in the ZSPL group. In addition, *Verrucomicrobia*, a beneficial bacterium, was increased in the ZSPL and ZSPH groups, even significantly higher than in the Normal group. On the genus level, as shown in Figure 3D, compared with the Normal group, the abundance of *norank_f_*Muribaculaceae was significantly increased in the Model group, and ZSPL and ZSPH reversed this increase. Concurrently, the abundance of the beneficial bacteria *Akkermansia* and *Allobaculum* was decreased in the Model group, while ZSPL and ZSPH increased their abundance. As shown in Figure 3E, the use of community heatmap to determine microbial populations on the genus level is consistent with the above analysis results.

#### 3.2.6 Beta-diversity analysis

Beta-diversity is a measure that refers to the comparison of microbial community composition and evaluates the differences among microbial communities (Cui et al., 2021). In this study, principal coordinates analysis and non-metric multidimensional scaling (NMDS) were used to determine beta diversity in each group. As shown in Figure 3F, the Model group was well distinguished from the Normal group in terms of the Bray-Curtis distance at the OTU level, and the composition of bacteria within the ZSPL and ZSPH groups was similar. NMDS analysis of the intestinal microbiota also showed a clear difference (Figure 3G).

#### 3.2.7 Test for significance of differences between groups

Taxon-based analysis also revealed changes in intestinal microbiota composition after ZSP treatment. The extended error bar showed significant differences in the mean proportions of bacterial taxa in samples from the Normal, Model, and ZSP groups. As shown in Figure 3H, the disruption of gut microbiota symbiosis in the Model group was characterized by a higher abundance of Lachnospiraceae*_NK4A136_group*, *norank_f_*Lachnospiraceae, Ruminococcaceae*_UCG-013*, and a lower abundance of *Akkermansia*, Coriobacteriaceae*_UCG_002*, *Dubosiella* and *Bifidobacterium* than in the Normal group. Moreover, as shown in Figure 3I, treatment with ZSPL significantly reduced the amount of *norank_f_*Muribaculaceae, *norank_f_*Lachnospiraceae (*p* = 0.0051 and *p* = 0.0202, respectively), and significantly promoted the relative abundance of *Akkermansia* (*p* = 0.0051) compared with that in the Model group. Concurrently, treatment with ZSPH significantly reduced the amount of *norank_f_*Muribaculaceae, *norank_f_*Lachnospiraceae (*p* = 0.0051 and *p* = 0.0131, respectively), and significantly promoted the relative abundance of *Akkermansia* (*p* = 0.0051) and *Psychrobacter* (*p* = 0.0200) compared with that in the Model group. The significant results were similar to the ZSPL group.

### 3.3 Therapeutic effect of ZSP on lung cancer based on the urethane-induced long-term lung cancer model

#### 3.3.1 Pathological changes of lung and colon tissue

**FIGURE 4:**
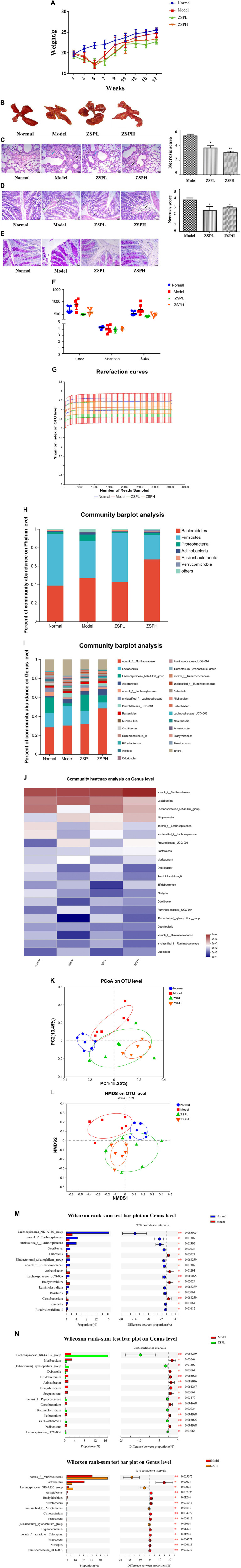
(Continued).

In order to more systematically study the effects of ZSP on the intestinal flora and intestinal barrier of lung cancer mice, our research team further supplemented the long-term model experiment of lung cancer induced by urethane (mainly observing lung and intestinal injury and intestinal flora). Firstly, the weight of the mice with lung cancer was observed over a long period. The weight of the mice reached its lowest point during the fifth week, followed by an upward trend (Figure 4A). At 17 weeks, lung cancer nodules were visible to the naked eye under natural light, indicating successful modeling (Figure 4B). As shown in Figure 4C, compared with the Normal group, the lung tissue in the Model group was severely damaged, the alveolar wall thickened in large areas with a small amount of inflammatory cell infiltration (black arrow) in the visual field, and perivascular edema was occasionally seen with a small amount of lymphocyte infiltration (blue arrow). The ZSP treatment groups showed mitigation of the degree of damage. The semi-quantitative score of the percentage of tumor tissue necrosis after resection showed that the Model group had the highest percentage (about 55%), while the ZSPH group had the lowest percentage (about 35%). Further evaluation of the pathological damage of the colon tissue using H&E (Figure 4D) and PAS staining (Figure 4E), when compared with the Normal group, the Model group showed a small amount of lymphocytic infiltration at the bottom of the lamina propria and a large area of alveolar wall thickening was observed in the colonic field. In the ZSP treatment groups, a small amount of lymphocyte infiltration was observed at the bottom of the lamina propria and the serosa layer in the colonic field, and thickening was reduced. Compared with the Normal group, the proportion of the Model group positive area showed hyperplasia. Compared with the Model group, the proportion of the positive areas of the ZSPL and ZSPH groups were significantly decreased. The semi-quantitative score of the percentage of colon tissue necrosis after resection showed that the Model group had the highest percentage (about 38%), while the ZSPL group had the lowest percentage (about 25%). Compared with the short-term lung cancer model, the long-term lung cancer model showed obvious pathological damage.

#### 3.3.2 Alpha-diversity of intestinal microbiota and rarefaction curves

As shown in Figure 4F, the Chao index of the Model group was significantly higher than the Normal and ZSP treatment groups, indicating that there may be more harmful bacteria in the Model group. The Shannon index showed no significant difference among the four groups. The coverage index of the four groups of mice were all greater than 0.995, indicating that the sequencing results could represent the genuine composition of intestinal bacteria in the samples. Rarefaction curves reflected that the depth of sequencing covered new rare phylotypes and of the majority of the diversity (Cui et al., 2021). Some flattening rarefaction curves were obtained, which confirmed that the microbial diversity was mostly obtained (Figure 4G).

#### 3.3.3 Composition and abundance of intestinal microbiota

As shown in Figure 4H, on the phylum level, six major bacterial phyla were identified. *Bacteroidetes*, *Firmicutes* and *Proteobacteria* were found to be predominant in all the samples. A relatively higher abundance of *Bacteroidetes* has been reported in the gut of lung cancer patients, consistent with these experimental results; however, ZSPL reversed this upward trend. *Firmicutes*, a beneficial bacterium, showed a decrease in abundance in the Model group; however, it was significantly increased in the ZSPL group. *Proteobacteria*, a harmful bacterium, when compared with the Normal group, showed a significant increase in the Model group. while the ZSP treatment groups showed a significant reduction in *Proteobacteria* abundance. As shown in Figure 4I, on the genus level, *norank_f_*Muribaculaceae, Lachnospiraceae*_NK4A136_group*, and *Alloprevotella* were beneficial bacterium. Compared with the Model group, ZSPL and ZSPH significantly increased the abundance of Lachnospiraceae*_NK4A136_group*, and ZSPH significantly increased the abundance of *norank_f_*Muribaculaceae and *Alloprevotella*. Prevotellaceae*_UCG-001*, a harmful bacterium, was significantly increased in the Model group, and ZSPL significantly reversed this increase. The use of community heatmap to determine microbial populations on the genus level is consistent with the above analysis results (Figure 4J).

#### 3.3.4 Beta-diversity analysis

As shown in Figure 4K, the Model group was well distinguished from the Normal group in terms of the Bray-Curtis distance at the OTU level, and the composition of bacteria within the ZSPL and ZSPH groups was similar. The dispersion was not as good as in the Lewis model. NMDS analysis of the intestinal microbiota also showed a difference (Figure 4L).

#### 3.3.5 Test for significance of differences between groups

As shown in Figure 4M, Lachnospiraceae*_NK4A136_group*, *norank_f_*Lachnospiraceae, *unclassified_f_*Lachnospiraceae and Lachnospiraceae*_UCG-006* had higher abundance in the Normal group, and *Dubosiella* and *Acinetobacter* had higher abundance in the Model group. As shown in Figure 4N, ZSPL significantly reduced the abundance of *Muribaculum* and *Dubosiella* (*p* = 0.0306), and significantly promoted the relative abundance of Lachnospiraceae*_NK4A136_group* (*p* = 0.0082) compared with the Model group. Concurrently, ZSPH significantly reduced the abundance of *Lactobacillus* and *Acinetobacter* (*p* = 0.0202 and *p* = 0.0077, respectively), and significantly promoted the relative abundance of *norank_f_*Muribaculaceae and Lachnospiraceae*_NK4A136_group* (*p* = 0.0051 and *p* = 0.0202, respectively) compared with the Model group.

## 4 Discussion

In this study, lung cancer was taken as the research object, ZSP was used as the model drug, “the exterior-interior relationship between the lung and the large intestine” was taken as the guiding theory and combined with modern technological methods to study the prevention and treatment of lung cancer with ZSP. Firstly, a Lewis short-term model of heterotopic transplanted tumor was established, and the effect of ZSP on the prevention and treatment of lung cancer preliminarily evaluated from the aspects of tumor inhibitory rate, immune organ index and protein expression in tumor tissues. After confirming the efficacy, the effects of ZSP on intestinal mucosal injury in mice with lung cancer were studied in relation to damage to the intestinal mucosal physical, immune, and biological barriers (i.e., intestinal flora). The effect of further supplementing a urethane-induced long-term lung cancer model on intestinal flora in mice was examined (select safe dose induction) (Sozio et al., 2021; Yu et al., 2022). And a combination of the two models used to study the prevention and treatment effect of ZSP on lung cancer from the perspective of “treating the lung from the intestine”.

The Lewis lung cancer model was established in C57BL/6 mice. The splenic and thymus indexes of the DDP group were also significantly lower than the Normal and Model groups (*p* < 0.01), indicating that DDP had inflicted significant damage to the immune function of the mice. Compared with the Model group, the spleen indexes of the ZSPL and ZSPH groups were increased, and the thymus indexes increased significantly (*p* < 0.01). Compared with the DDP group, the spleen and thymus indexes of the ZSPL and ZSPH groups were increased significantly (*p* < 0.01). Cisplatin has side effects in the digestive tract. In the experiment, it was also observed that the amount of food and water consumed by mice in the DDP group was significantly less than that of other groups. Cisplatin can damage immune organs (spleen and thymus) and reduce weight, renal toxicity has also been reported in literature (Dasari and Bernard, 2014; Yam et al., 2015; Ying et al., 2016). H&E staining was then used to observe the pathological injury of the tissue. In the short-term model, the colon tissue of normal and tumor-bearing mice showed no significant pathological changes. Ki67 is a nuclear protein involved in cell proliferation and closely related to RNA transcription. It is not only expressed in the G1, S, G2 and M phases of the cell cycle, but it can also be used to evaluate the proliferation ratio of a cell population, and thus used as a marker of cell proliferation (Cheng et al., 2020a). p53 is a kind of nucleoprotein, transcription factor and tumor suppressor, many related to cancer cells can respond to stress source, including DNA damage, cancer gene signal transduction and metabolic disorders, its function is to induce cell cycle arrest and apoptosis, senescence, DNA repair and change the metabolism in response to these cellular stressors (Oduah and Grossman, 2020). Immunohistochemical analysis of tumor tissues showed that ZSP could reduce the expression of Ki67 protein and increase the expression of p53 protein. In this study, the expression of Ki67 in lung cancer tissues was significantly higher than in Normal lung tissues, which was consistent with the clinical conclusion that the proliferation of Ki67 increases with the increase in malignancy of tumor cells. With the aggravation caused by lung cancer, the expression of p53 also decreases, which is consistent with the clinical trend; however, the administration of ZSP could reverse this trend. The development of tumors is an extremely complex pathological process with many influencing factors. Previous studies have shown that the abnormal expression of inflammatory factors is closely related to the occurrence and development of tumors (Daou, 2020). On one hand, inflammatory factors play a regulatory role in the tumor immune defense response, while on the other hand, inflammatory factors can also promote tumor growth and metastasis (Taranu et al., 2018). Like most solid tumors, the pathogenesis of NSCLC is closely related to the levels of many inflammatory factors. Some studies have shown that the sustained high expression of TNF-α can promote tumor growth and invasion, and patients with high expression of TNF-α usually have high metastasis and recurrence rates (Liao et al., 2019; Wang et al., 2019a). IL-1β is mainly synthesized and secreted by mononuclear macrophages, and IL-6 is produced by T and B lymphocytes and monocytes. In addition to participating in inflammatory responses and anti-infection defenses, IL-1β and IL-6 also participate in the development and metastasis of some malignant tumors. Some studies have shown that the level of IL-1β is higher in patients with lung cancer (Peukert et al., 2021). IL-6 has been proven to promote tumor angiogenesis and enhance tumor metastasis and invasion (Weber et al., 2021). The levels of cytokines in the mouse blood were detected using an ELISA kit and it was found that the levels of IL-1β, IL-6 and TNF-α were decreased by administration of ZSP. The experimental results show that ZSP can inhibit the proliferation of tumor cells, significantly increase the level of tumor suppressors, and reduce the level of inflammatory factors, indicating that ZSP can play a role in the prevention and treatment of lung cancer by improving the immune function of the mouse body.

The foundation of the intestinal mucosal barrier is the physical barrier (Yang et al., 2021). The physical barrier is composed of intestinal mucosal epithelial cells, tight junction proteins and biofilm, which effectively prevent harmful substances such as bacteria from entering the blood through the intestinal mucosa and prevent a series of inflammatory reactions (Qi et al., 2020). Once this intestinal mucosal barrier is damaged, intestinal function becomes abnormal and the health of the body becomes endangered to different degrees. In this study, the extent of intestinal mucosal injury of lung cancer mice was investigated by pathological examination. The expression of ZO-1, Occludin and Claudin-1 protein in lung cancer mice were detected, and the repair effect on the intestinal mucosal physical barrier in lung cancer mice was investigated. Occludin is a key TJ protein that interacts with intracellular signaling pathways to adjust and maintain intestinal permeability. ZO-1 is a peripheral membrane protein that interacts with Occludin, then indirectly interacts with actin to maintain tight junction stability and permeability (Jiang et al., 2021; Li et al., 2021). Claudin is a major component of tight junctions, which act as a major barrier to paracellular spread, and it also maintains cell polarity in normal epithelium and endothelial cells (Yamamoto et al., 2020). In cancer cells, in addition to being a component of tight junctions, Claudin also plays other roles and is involved in acellular division or invasion, among which Claudin-1 has the greatest potential in the diagnosis and prognosis of many types of cancer (Yamamoto et al., 2020). The detection results showed that the expression of ZO-1, Occludin and Claudin-1 in the Model group were decreased, and ZSP can alleviate the protein expression changes in the colon tissue caused by lung and intestinal pathological translocation to a certain extent, improving the intestinal physical barrier.

The determination of sIgA content in the BALF is conducive to revealing the local immune status of lung tissues in different diseases. sIgA is one of the material bases of “the exterior-interior relationship between the lung and the large intestine”. sIgA can suppress and exclude pathogenic factors, neutralize toxins through immune responses, and protect mucosal function through its immune barrier function. The quantities of sIgA in the BALF and colon of the ZSP groups were increased, and the quantities of sIgA in the ZSPH group was significantly increased (*p* < 0.05, *p* < 0.01). The change in the BALF was consistent with those in the colon. The results showed that ZSP had a protective effect on the intestinal mucosal immune barrier in lung cancer mice.

The results show that by protecting the immune function of the body and the physical and immune barriers of the intestinal mucosa, the intestinal damage can be alleviated, the immunity can be increased, and the pathogenic bacteria can be precluded from invading the intestine, so as to achieve the prevention and treatment of lung cancer. In the short-term model, there was no obvious pathological change in the colon tissue of mice; therefore, it was illustrated that the pathological transmission between lung and intestine were more reflected in the micro-regulation of these factors.

The intestinal microbiota is one of the most studied microecosystems due to the abundant microbial communities in the intestine, and the characteristics of the gastrointestinal microbial community can be analyzed through fecal samples, which are often easy to obtain (Budden et al., 2017). Recent studies have reported the role of intestinal microbiota in respiratory diseases. This relationship is called the “gut-lung axis”, and intestinal microbiota is considered to play a crucial role in this axis (Zhang et al., 2020). The structural changes in intestinal microbiota are closely related to the occurrence and development of lung cancer (Cheng et al., 2020b). The study found that the structure of the intestinal microbiota of the lung cancer model had changed compared with that of the healthy model. The intestinal microbiota of the lung cancer model had a higher relative abundance of *Bacteroidetes*, *Fusobacteria*, *Bacteroides*, *Veillonella and Fusobacterium,* while having a lower relative abundance of *Firmicutes, Proteobacteria* and *Escherichia coli Shigella* (Houghton et al., 2016; Zhuang et al., 2019). Liu et al. also confirmed that the intestinal microbiota structure of lung cancer patients had changed significantly compared with that of healthy people (Liu et al., 2019). Zheng et al. also established a specific intestinal microbiota characteristic model that can effectively and accurately predict early lung cancer (Zheng et al., 2020).

In this study, a short-term Lewis lung cancer model and a long-term urethane-induced lung cancer model were constructed to investigate the changes in the intestinal microbiota structure in lung cancer mice. The results showed that the composition of *Firmicutes, Bacteroidetes, Proteobacteria, Verrucomicrobia* and *Actinobacteria* changed in a short period of time in lung cancer mice. In the long-term model induced by urethane, *Firmicutes, Bacteroidetes* and *Proteobacteria* showed significant changes. The number of intestinal microbiota is complex and diverse. According to the significantly changed microbiota screened by the two lung cancer models, the relationship between intestinal microbiota and lung cancer is briefly expounded as follows. The abundance of *Bacteroidetes* was higher in the intestine of lung cancer patients, while *Firmicutes* and *Proteobacteria* were often less abundant (Zhang et al., 2018b), which was consistent with the results of our short-term lung model. Following ZSP intervention, the relative abundance of these three bacteria could be restored. *norank_f_*Muribaculaceae belongs to a genus of bacteria in the family *Muribaculacea* under the order *Bacteroides*. Muribaculaceae comes from the family S24-7 and was recently named by Lagkouvardos I et al. This family is highly abundant in the intestinal tract of mice, but its function is still unclear (Lagkouvardos et al., 2019). In this experiment, *norank_f_*Muribaculaceae was highly abundant in all groups, and the abundance of this genus significantly increased following establishment of the Lewis lung cancer models, while the abundance of this genus significantly decreased following treatment with ZSP in the lung cancer mice. These results indicate that the changes in this genus play an important role in the occurrence and development of lung cancer and it is also an important target bacteria for ZSP in relation to its anticancer effect. *Verrucomicrobia* are enriched in the intestinal mucus layer and represented by *Akkermansia*. *Akkermansia* is abundant in the human intestine and colonizes the colon with a relative abundance of 1%–4% (Ouwerkerk et al., 2013). *Akkermansia*, is also abundant in human feces, can degrade gastrointestinal mucinase and plays an important role in NSCLC (Derosa et al., 2022). There is evidence to show that ZSP increases the content of *Akkermansia* in the intestine, improves the intestinal barrier function, regulates the body’s immunity, and then plays an inhibitory role in tumor growth (Wang et al., 2019b). *Allobaculum* is a butyrate producing bacterium. Butyrate in the intestine is widely believed to play a key role in maintaining the nutrition of intestinal epithelial cells, enhancing the intestinal mucosal barrier, inhibiting abnormal immune activation in the intestine and maintaining intestinal homeostasis (Chen and Vitetta, 2018; Fu et al., 2019; Chen et al., 2020). However, ZSPL and ZSPH can increase the abundance of *Allobaculum*, therefore repairing intestinal barrier damage, providing energy for intestinal epithelial cells, and maintaining the stability of the intestinal microecology. Lachnospiraceae is enriched in the folds of intestinal mucosa, which directly interacts with intestinal mucosal immune cells, prevents adhesion and colonization of intestinal pathogens, is responsible for the degradation of various fibers and polysaccharides, and maintains intestinal health. Lachnospiraceae is also an important butyric acid-producing bacterium, which accelerates the differentiation of regulatory T cells in dendritic cells and reduces inflammation (Yang et al., 2018). Therefore, the conclusion is drawn that Lachnospiraceae can protect intestinal mucosa, reduce inflammation, and improve immune production in the prevention and treatment of lung cancer. Lachnospiraceae*_NK4A136_group, norank_f_*Lachnospiraceae and *unclassified_f_*Lachnospiraceae are genera of bacteria in the family Lachnospiraceae under the order *Clostridium*. In the urethane-induced lung cancer model, the abundance of Lachnospiraceae*_NK4A136_group*, *norank_f_*Lachnospiraceae and *unclassified_f_*Lachnospiraceae decreased, and ZSP could reverse this trend. These results indicate that ZSP can increase the microorganisms beneficial to the body health, prevent inflammation and protect the intestinal tract from a variety of pathogens and bacteria, to improve the body’s immunity.

*Acinetobacter*, is a genus of bacteria in the family Moraxellaceae. Moraxellaceae is the main pathogenic bacteria of upper respiratory tract disease in children (Adams and Brown, 2019), can cause sinusitis and otitis media, and is especially pathogenic in patients with low immunity (Yang et al., 2015). The urethane-induced model showed a significant increase in the abundance of the harmful *Acinetobacter* bacterium and its level decreased following administration of ZSP. Prevotellaceae*_UCG-001* is a genus of bacteria in the family Prevotellaceae, Members of the family Prevotellaceae have previously been shown to increase the severity of dextran sulfate sodium-induced colitis in mice. (Ibrahim et al., 2019). In the lung cancer model induced by urethane, the abundance of Prevotellaceae*_UCG-001* in the Model group was significantly increased compared with the Normal group, and the abundance of Prevotellaceae*_UCG-001* was significantly decreased following administration of ZSP. This suggests that ZSP can inhibit the growth of these harmful bacteria during the development of lung cancer.

In summary, ZSP prevents the translocation of intestinal microbiota, inhibits the transfer of pathogenic bacteria, improves the structure of intestinal microbiota, reverses the imbalance of intestinal microecology, plays a role in the prevention and treatment of lung cancer by regulating the intestinal physiological barrier. In this paper, the effect of lung cancer on the intestine and the regulation of Zengshengping on the intestinal barrier and flora were discussed. In the future, we will further verify the hypothesis through quantitative analysis of differential bacteria and fecal colonization, and clarify the relationship between gut microbial function and recovery of lung cancer.

## 5 Conclusion

In this study, we used the Lewis lung cancer model, H&E staining, ELISA, immunohistochemistry and other techniques to investigate the development of lung cancer, tumor cell proliferation, immune organ index, inflammatory factor level, Ki67 and p53 and tight junction protein expression, to evaluate the efficacy of ZSP in the prevention and treatment of lung cancer. Protein immunoblotting, immunohistochemistry and other methods were used to investigate the physical barrier and immune barrier of the intestinal mucosa, 16Sr high-throughput sequencing technology was used to investigate the intestinal mucosal biological barrier of Lewis lung cancer model mice and urethane-induced lung cancer model mice, and evaluate the effect of ZSP on intestinal mucosa of lung cancer mice. “Lung cancer”, “gut-lung axis” and “treating the lung from the intestine” are three aspects of progressive development, and the role of ZSP in the prevention and treatment of lung cancer is expounded.

## Data Availability

The data presented in the study are deposited in the NCBI repository, accession number PRJNA938874.

## Abbreviations

BALF, bronchoalveolar lavage fluid; DDP, Cisplatin; DMSO, Dimethyl sulfoxide; ELISA, Enzyme-linked immunosorbent assay; HRP, horseradish peroxidase; H&E, Hematoxylin and eosin; NSCLC, non-small cell lung cancer; NMDS, non-metric multidimensional scaling analysis; OTUs, operational taxonomic units; PBS, phosphate-buffered saline; PAS, Periodic acid-Schiff staining; ZSP, Zengshengping; TCM, traditional Chinese medicine; sIgA, secretory immunoglobulin A; TNF-α, tumor necrosis factor α; PCR, polymerase chain reaction; ZSPL, ZSP low-dose; ZSPH, ZSP high-dose.
