# Supplementary material for: Zengshengping improves lung cancer by regulating the intestinal barrier and intestinal microbiota
Source: Front Pharmacol. 2023 Mar 13;14:1123819. doi: 10.3389/fphar.2023.1123819 (PMC10040556; doi:10.3389/fphar.2023.1123819)
Supplement: Supplementary file 1 [file DataSheet3.docx]

***Supplementary Material***

**Zengshengping improves lung cancer by regulating the intestinal barrier and intestinal microbiota**

**E Sun^1,^****^2^**^†^**, Xiangqi Meng****^1,2^**^†^**, Zhaoxia Kang^1,2^, Huimin Gu^1^, Mingyu Li^1^, Xiaobin Tan^1,2^,** **Liang Feng^3*^, Xiaobin Jia^1,3*^**

^1^Affiliated Hospital of Integrated Traditional Chinese and Western Medicine, Nanjing University of Chinese Medicine, Nanjing 210028, China

^2^Key Laboratory of New Drug Delivery System of Chinese Meteria Medica, Jiangsu Provincial Academy of Chinese Medicine, Nanjing 210028, China

^3^School of Traditional Chinese Pharmacy, China Pharmaceutical University, Nanjing 211198, China

***Correspondence:**

Liang Feng, [wenmoxiushi@163.com](mailto:wenmoxiushi@163.com)

Xiaobin Jia, jiaxiaobin2015@163.com

1. **Supplementary** **Tables and Figures**

**1.1 Supplementary Tables 1.** Body weight of Lewis lung cancer mice (g)

|  | **Normal** | | **Model DDP** | | | | **ZSPL** | | | **ZSPH** | | |
| --- | --- | --- | --- | --- | --- | --- | --- | --- | --- | --- | --- | --- |
| Day | Mean | SD | Mean | SD | Mean | SD | | Mean | SD | Mean | SD |  |
| 1 | 21.71 | 0.82 | 21.54 | 0.91 | 21.36 | 0.68 | | 21.53 | 1.19 | 21.34 | 1.18 |  |
| 3 | 21.23 | 0.88 | 21.26 | 0.78 | 19.21 | 0.75 | | 20.85 | 1.36 | 20.70 | 1.25 |  |
| 5 | 21.39 | 1.26 | 20.70 | 0.89 | 16.57 | 0.75 | | 21.50 | 1.99 | 20.86 | 1.51 |  |
| 7 | 22.63 | 1.09 | 22.00 | 1.34 | 15.23 | 0.84 | | 21.88 | 0.96 | 21.76 | 1.73 |  |
| 9 | 21.81 | 1.73 | 20.67 | 1.24 | 14.61 | 1.15 | | 22.65 | 1.04 | 22.59 | 1.71 |  |
| 11 | 22.31 | 1.44 | 21.34 | 1.28 | 14.35 | 1.24 | | 22.52 | 1.06 | 22.93 | 1.44 |  |
| 13 | 22.03 | 0.89 | 21.54 | 1.26 | 15.46 | 1.67 | | 22.87 | 1.28 | 22.77 | 1.43 |  |
| 15 | 23.10 | 1.12 | 21.84 | 1.22 | 14.94 | 1.55 | | 22.95 | 1.27 | 23.30 | 1.23 |  |

## 1.2 Supplementary Figures

##
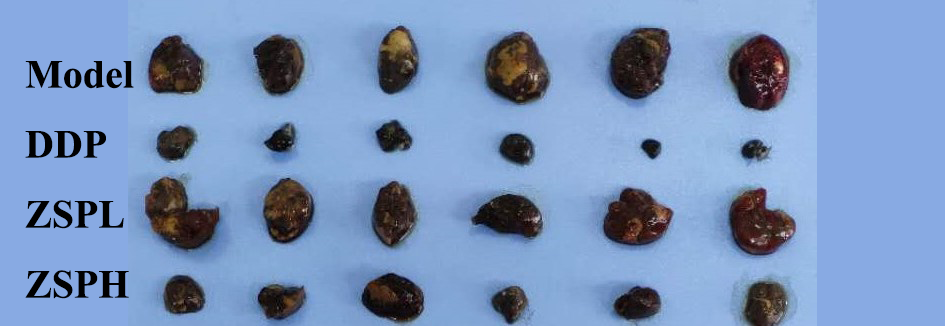


**Supplementary Figure 1.** Tumor picture


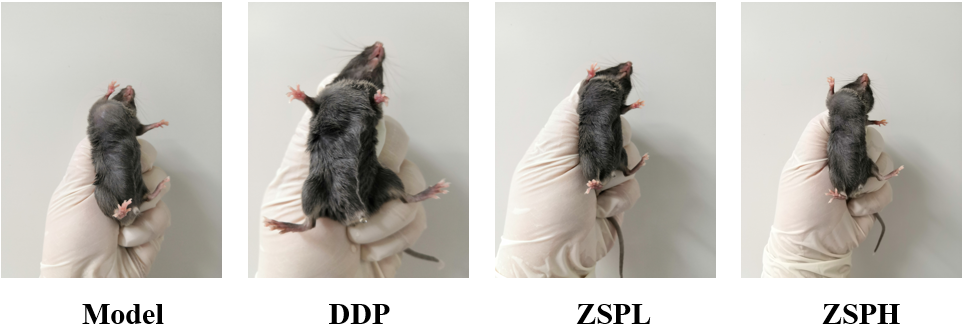


**Supplementary Figure 2.** Mice picture


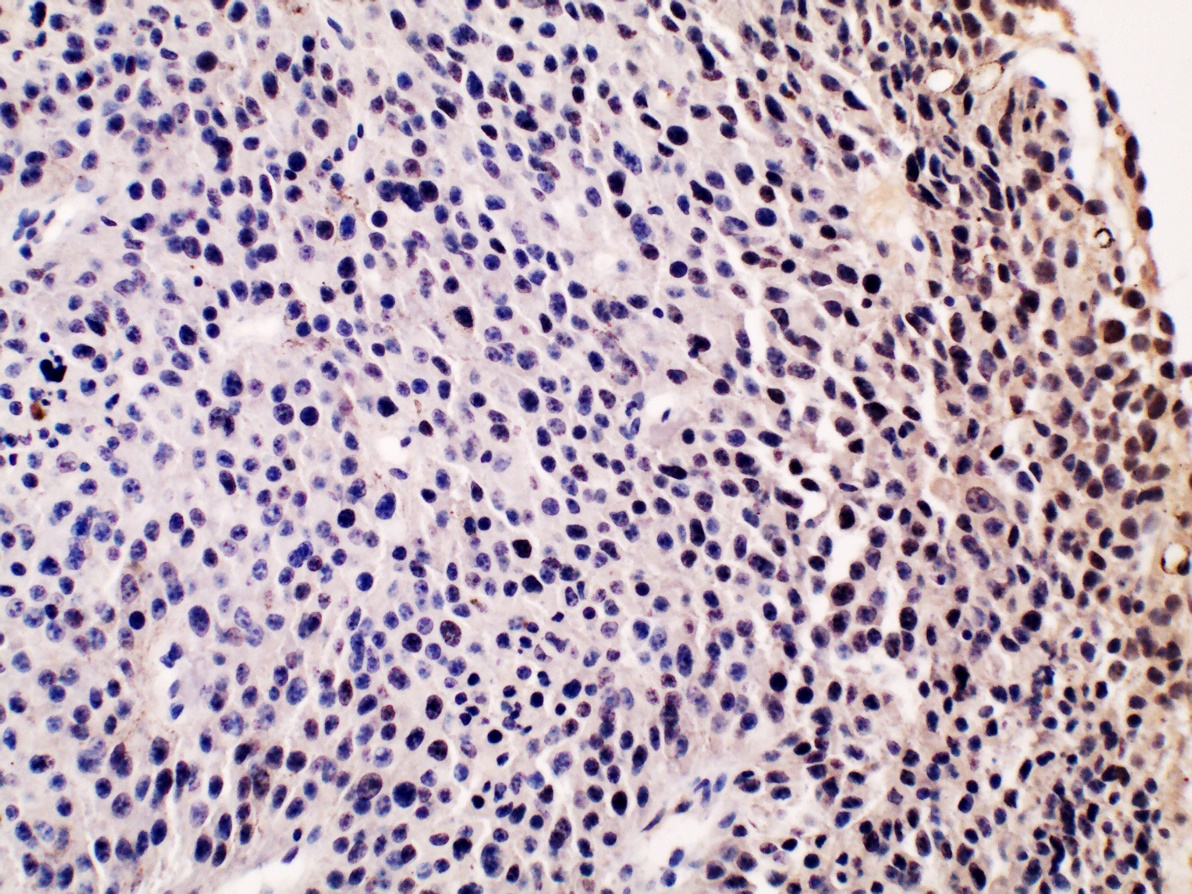


**Supplementary Figure 3.** Expression of Ki67 (Model, ×400)


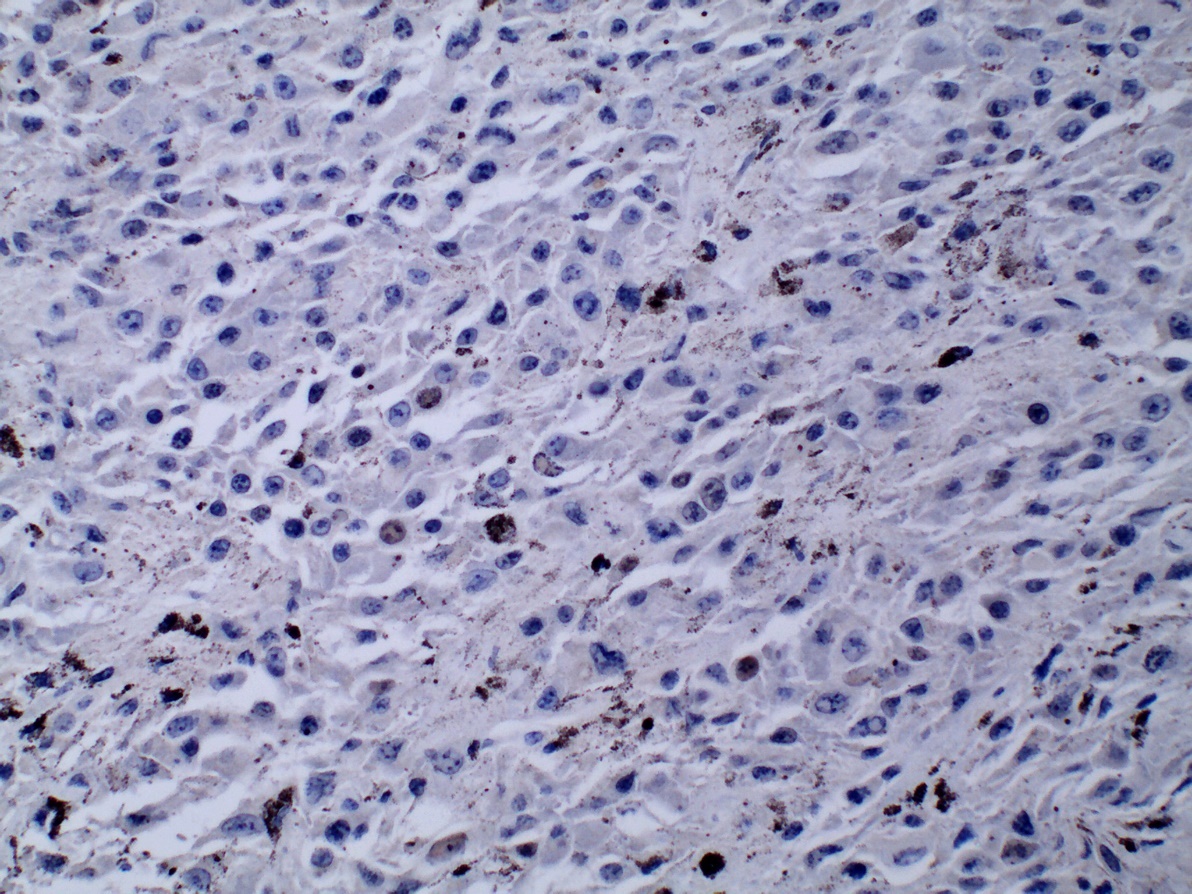


**Supplementary Figure 4.** Expression of Ki67 (DDP, ×400)


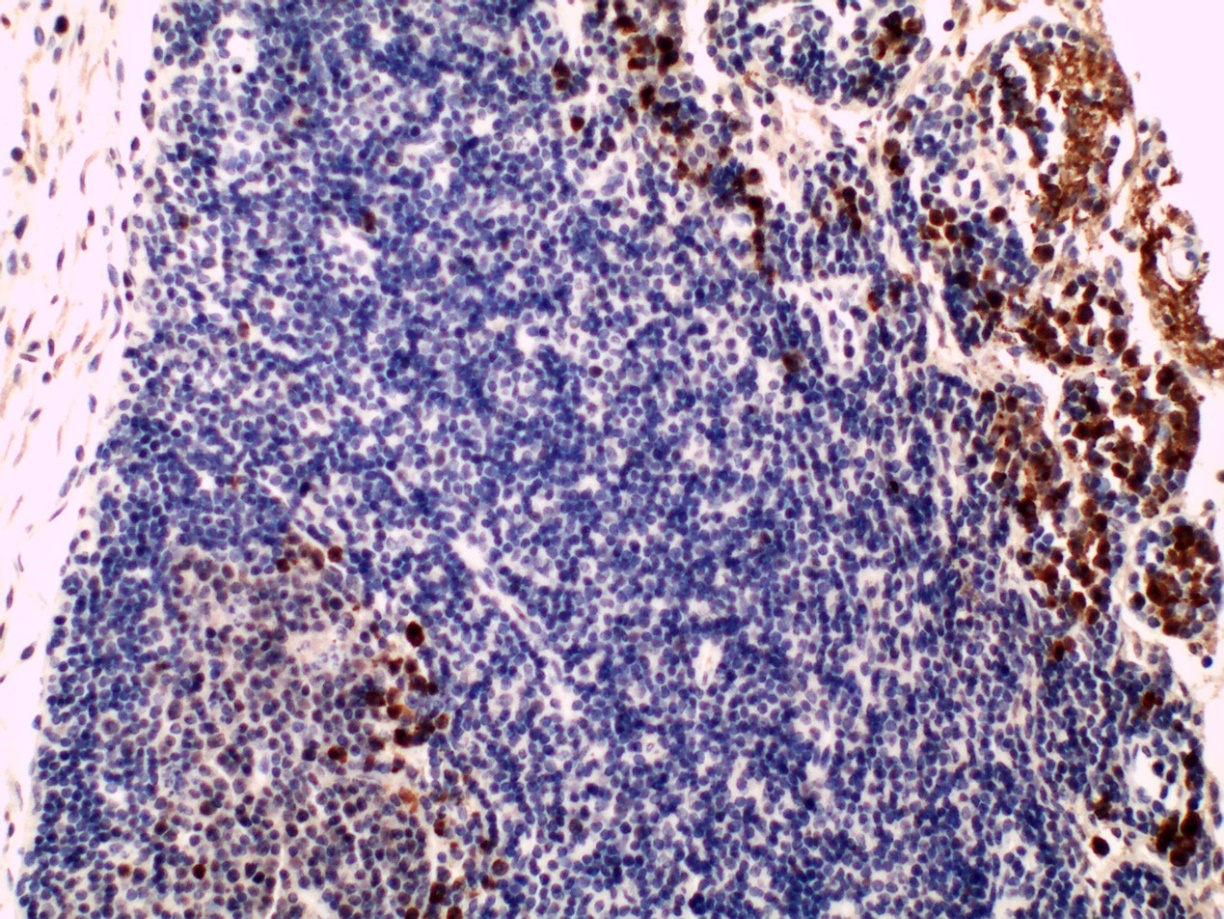


**Supplementary Figure 5.** Expression of Ki67 (ZSPL, ×400)


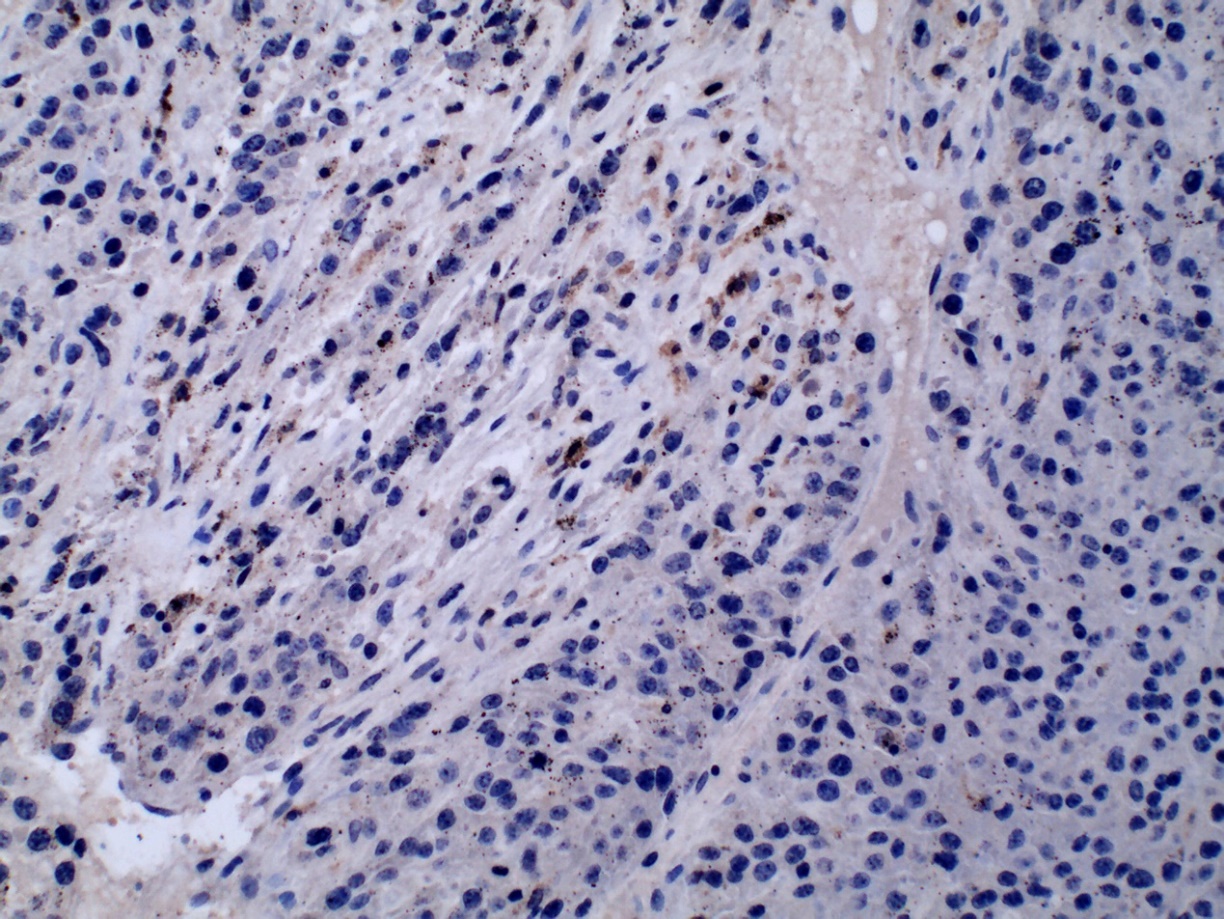


**Supplementary Figure 6.** Expression of Ki67 (ZSPH, ×400)


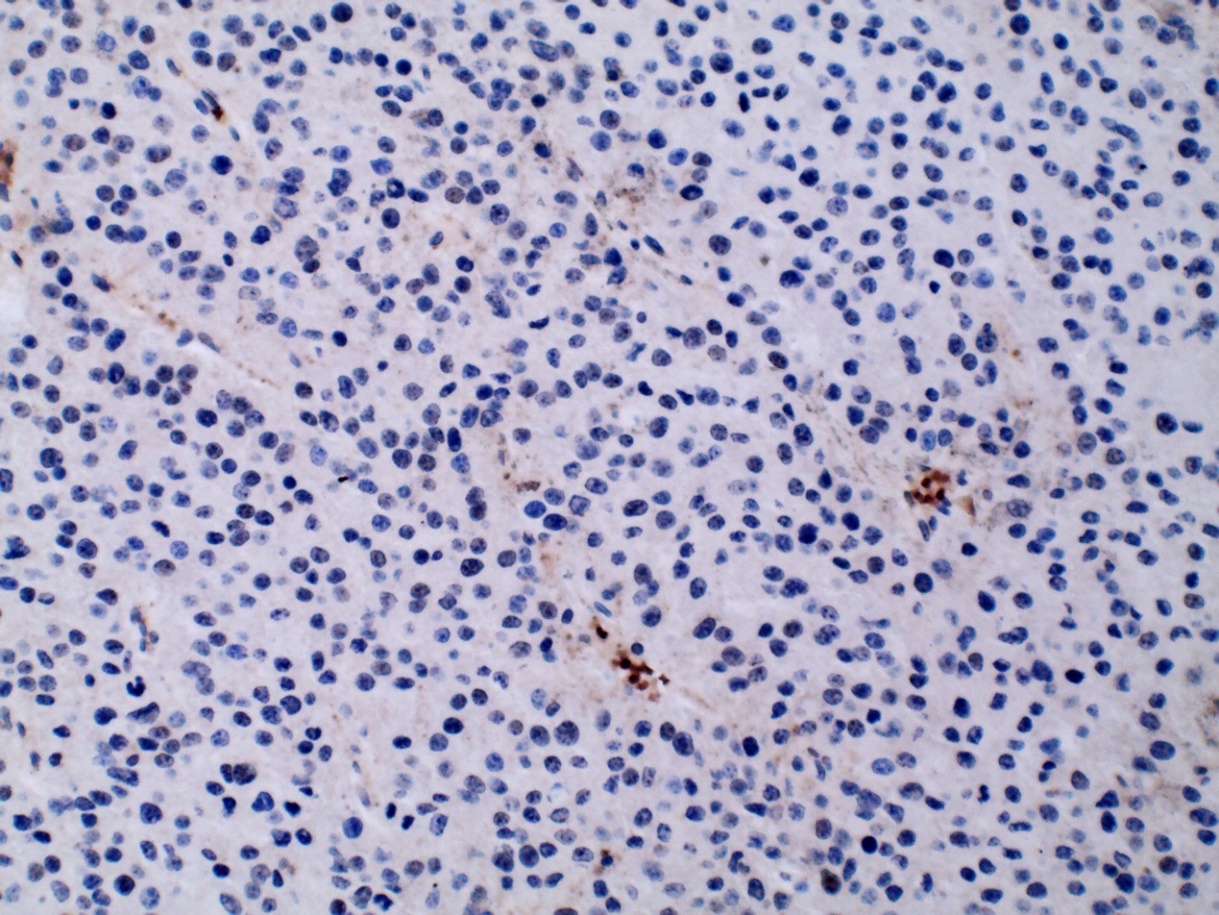


**Supplementary Figure 7.** Expression of p53 (Model, ×400)


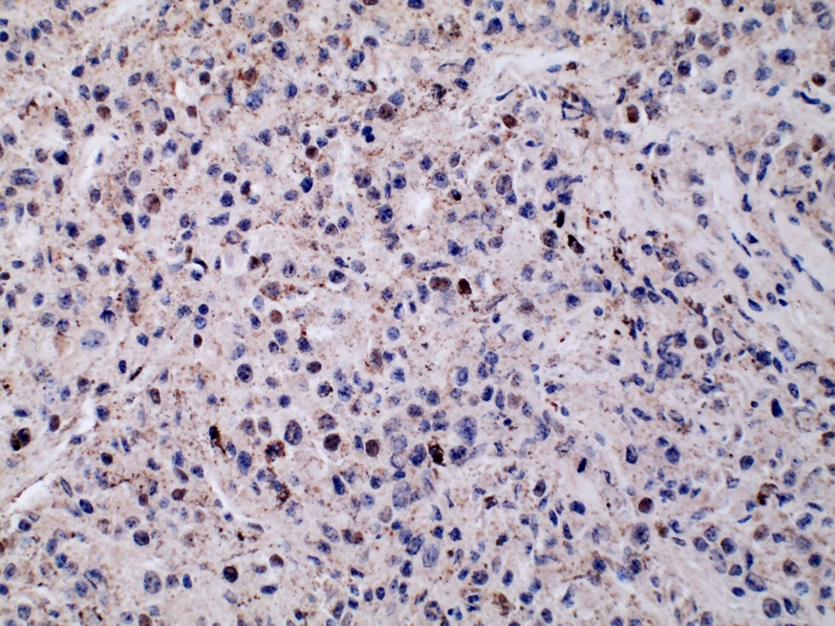


**Supplementary Figure 8.** Expression of p53 (DDP, ×400)


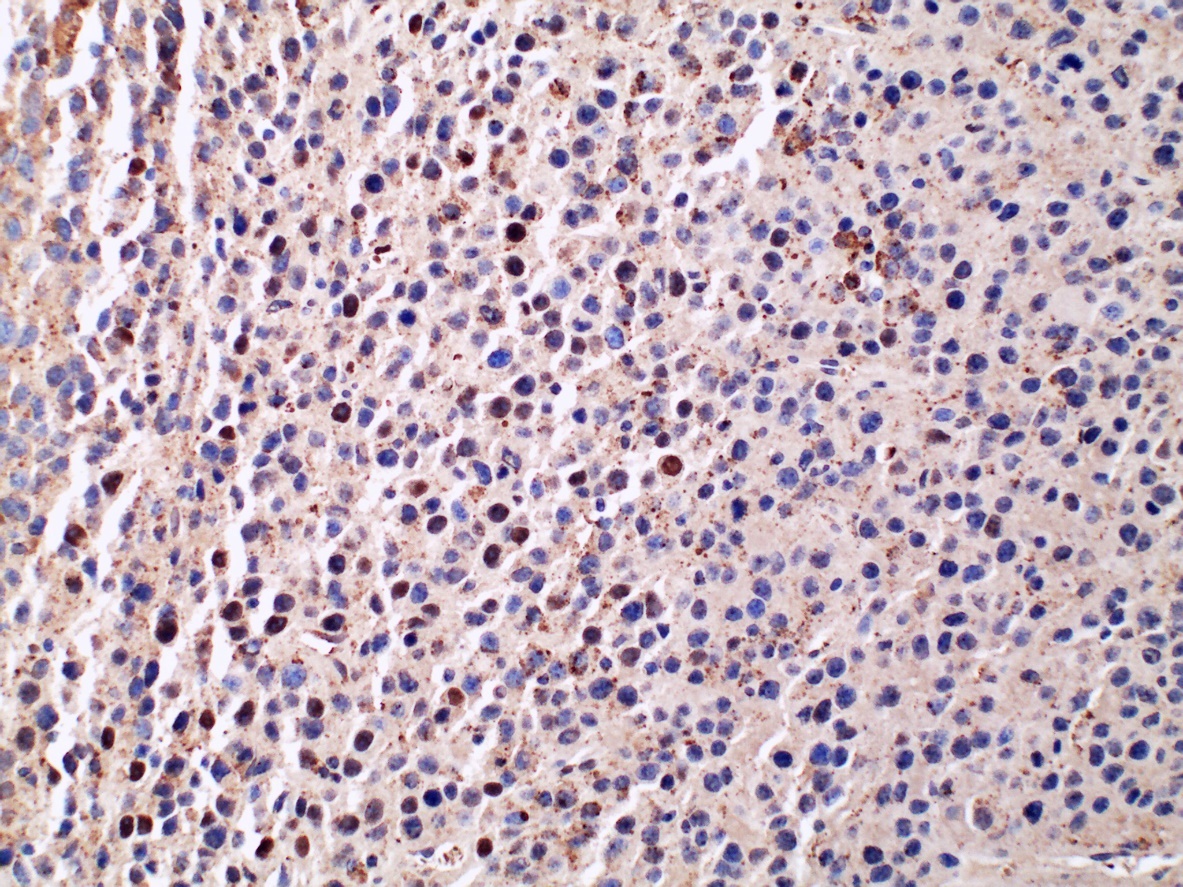


**Supplementary Figure 9.** Expression of p53 (ZSPL, ×400)


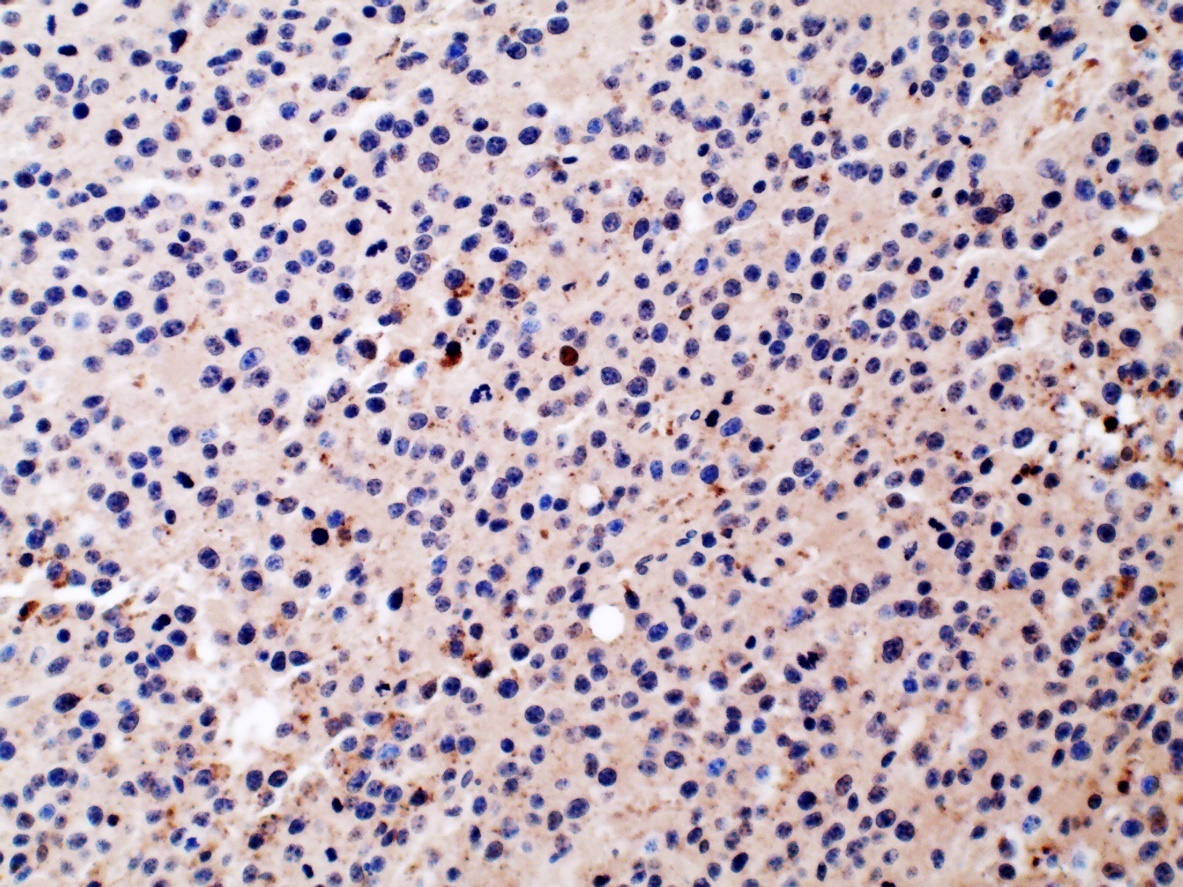


**Supplementary Figure 10.** Expression of p53 (ZSPH, ×400)


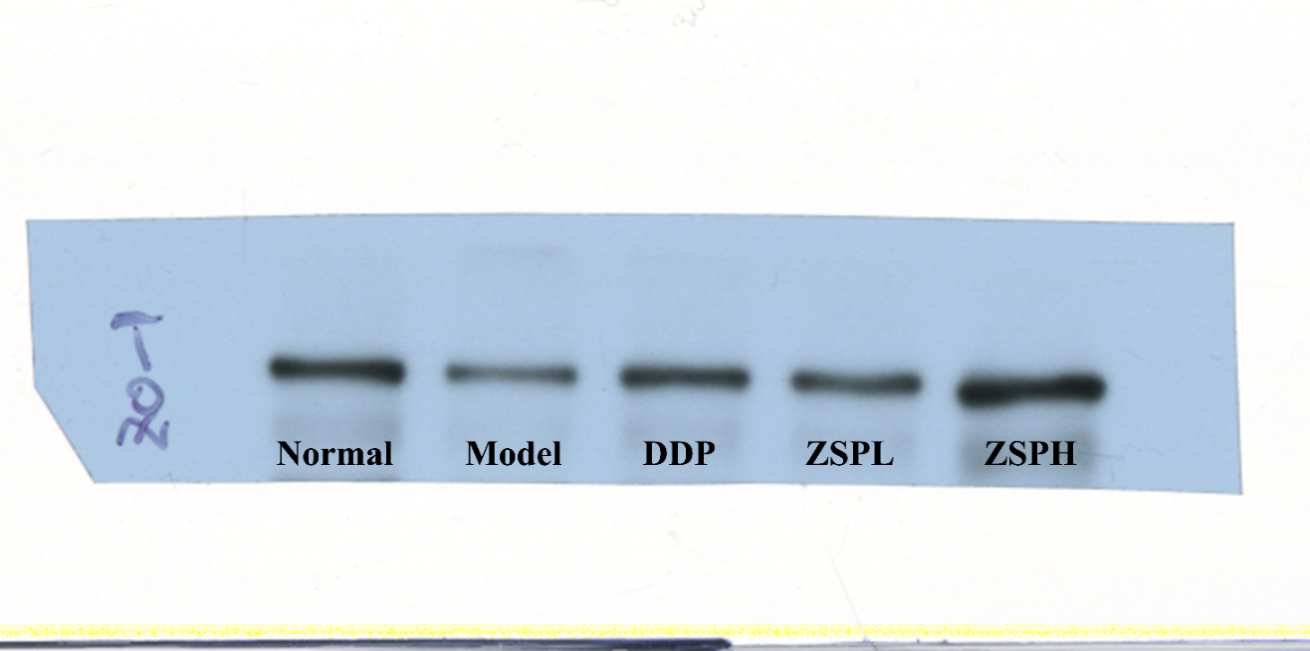


**Supplementary Figure 11.** Western blot original stripe (ZO-1)


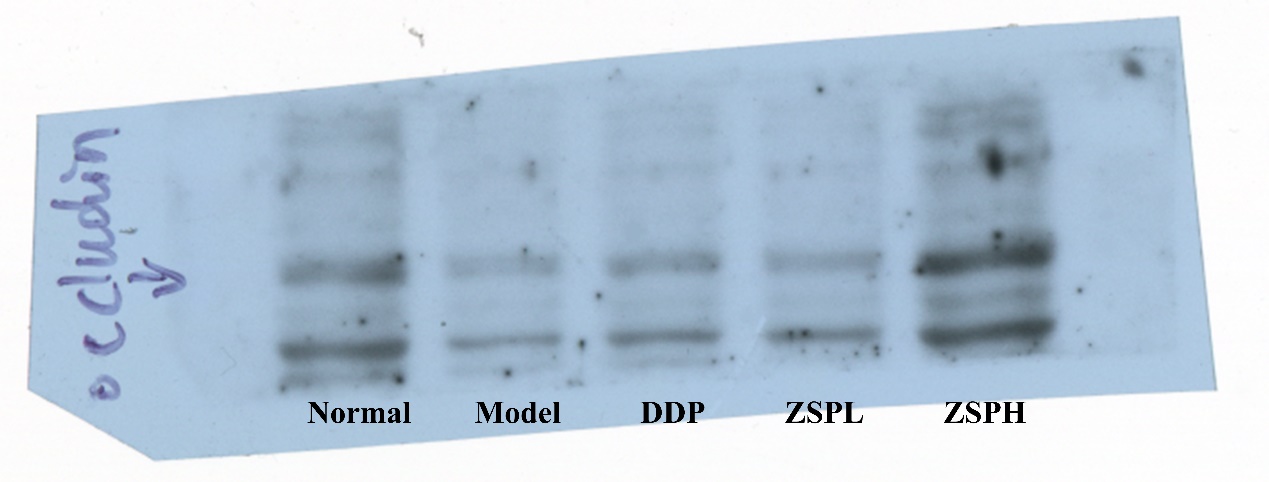


**Supplementary Figure 12.** Western blot original stripe (occludin)


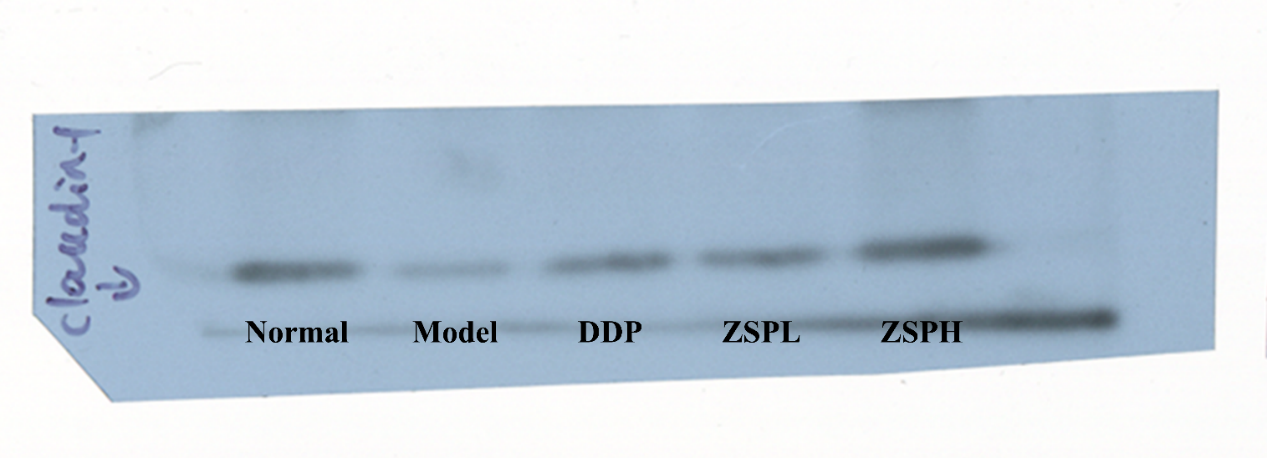


**Supplementary Figure 13.** Western blot original stripe (Claudin-1)


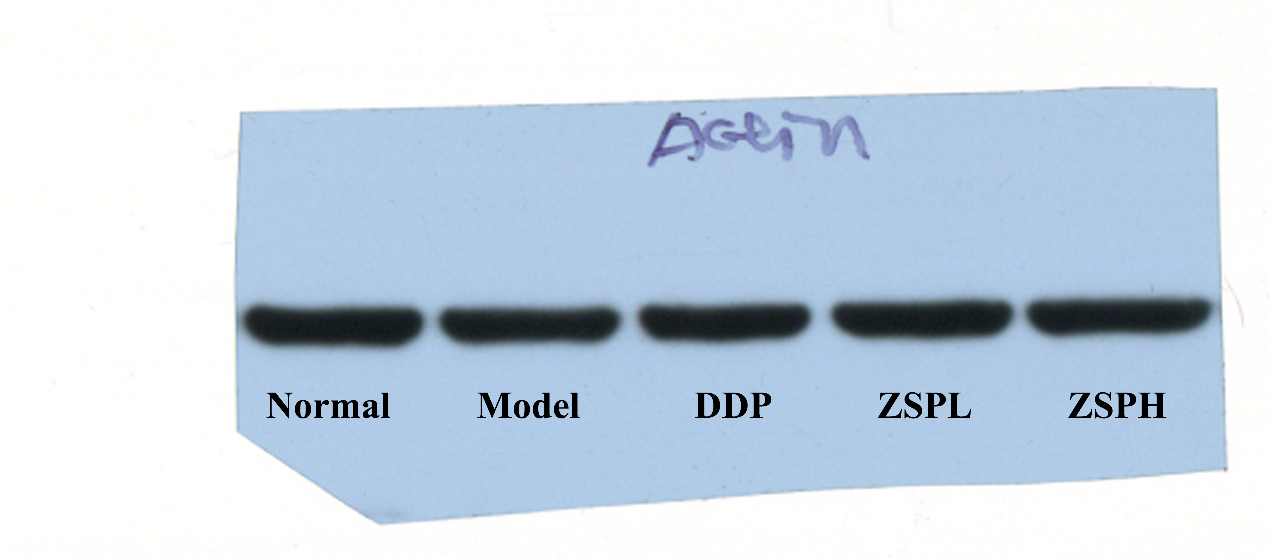
 **Supplementary Figure 14.** Western blot original stripe (Actin)
