# Supplementary material for: Zengshengping improves lung cancer by regulating the intestinal barrier and intestinal microbiota
Source: Front Pharmacol. 2023 Mar 13;14:1123819. doi: 10.3389/fphar.2023.1123819 (PMC10040556; doi:10.3389/fphar.2023.1123819)
Supplement: Supplementary file 2 [file DataSheet1.docx]

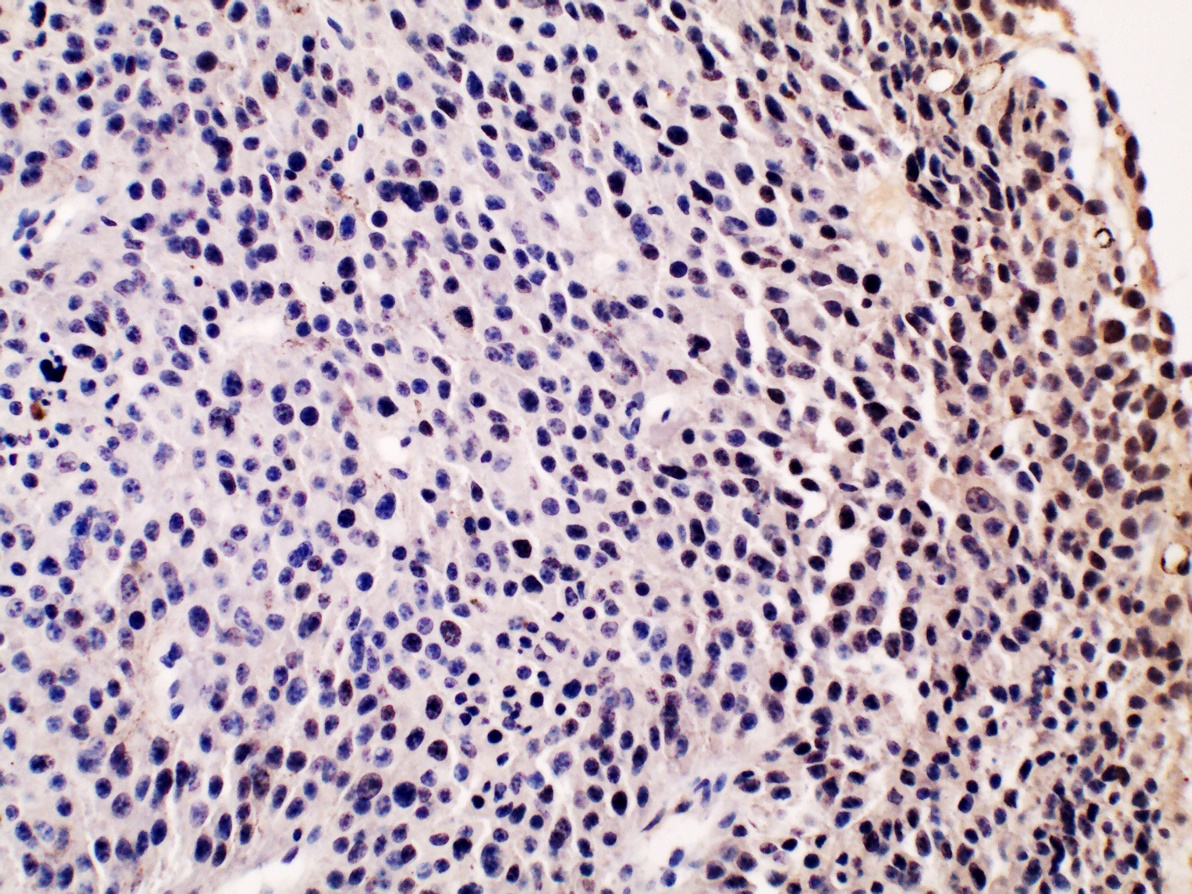


Figure 1 Expression of Ki67 (Model, ×400)


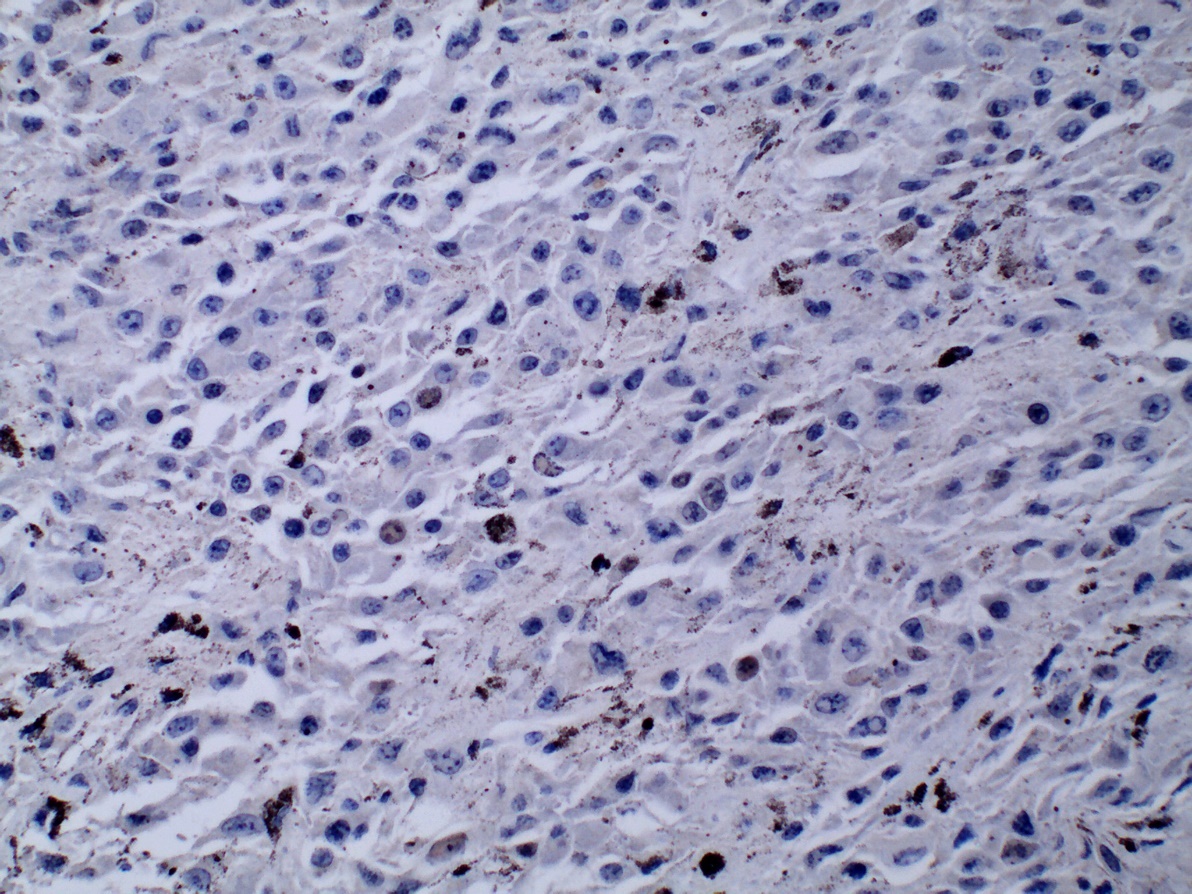


Figure 2 Expression of Ki67 (DDP, ×400)


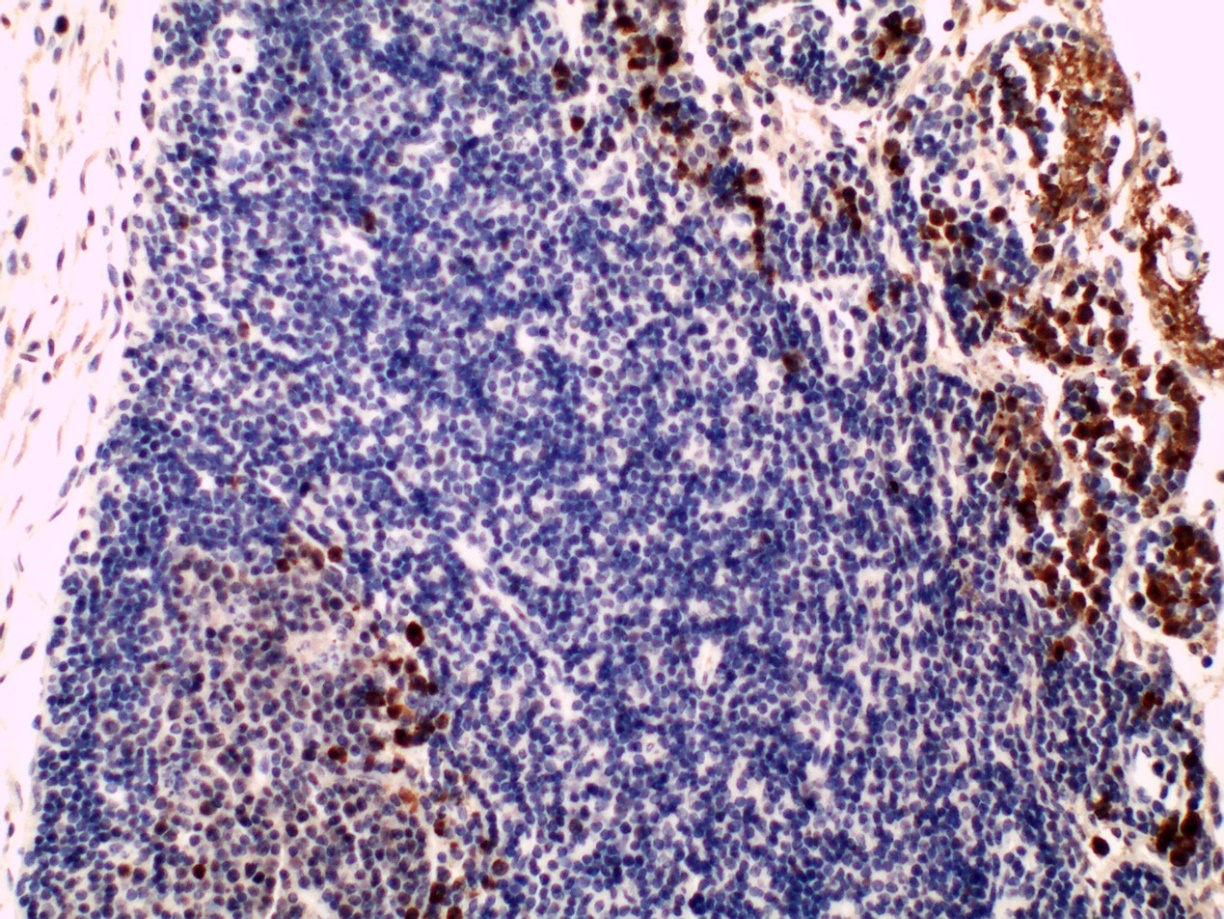


Figure 3 Expression of Ki67 (ZSPL, ×400)


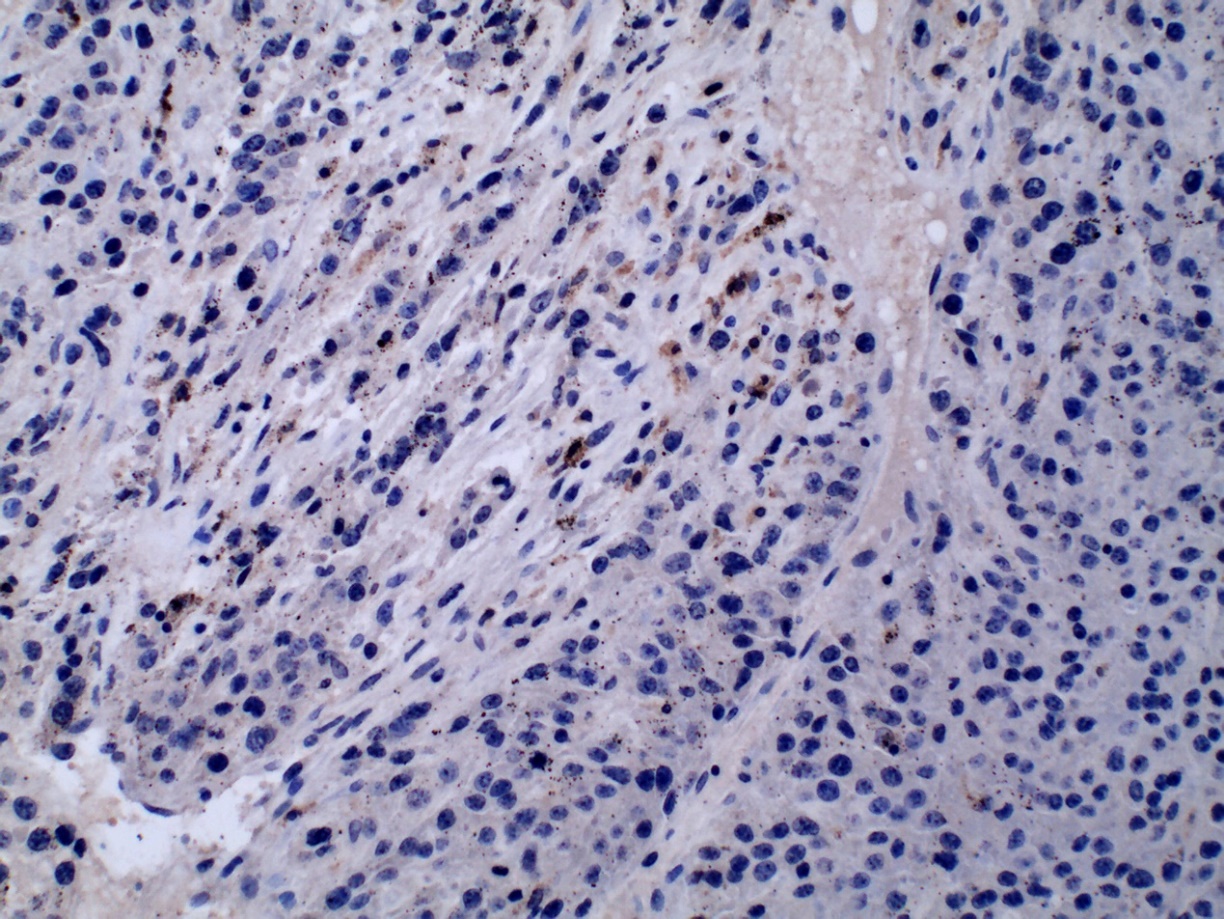


Figure 4 Expression of Ki67 (ZSPH, ×400)


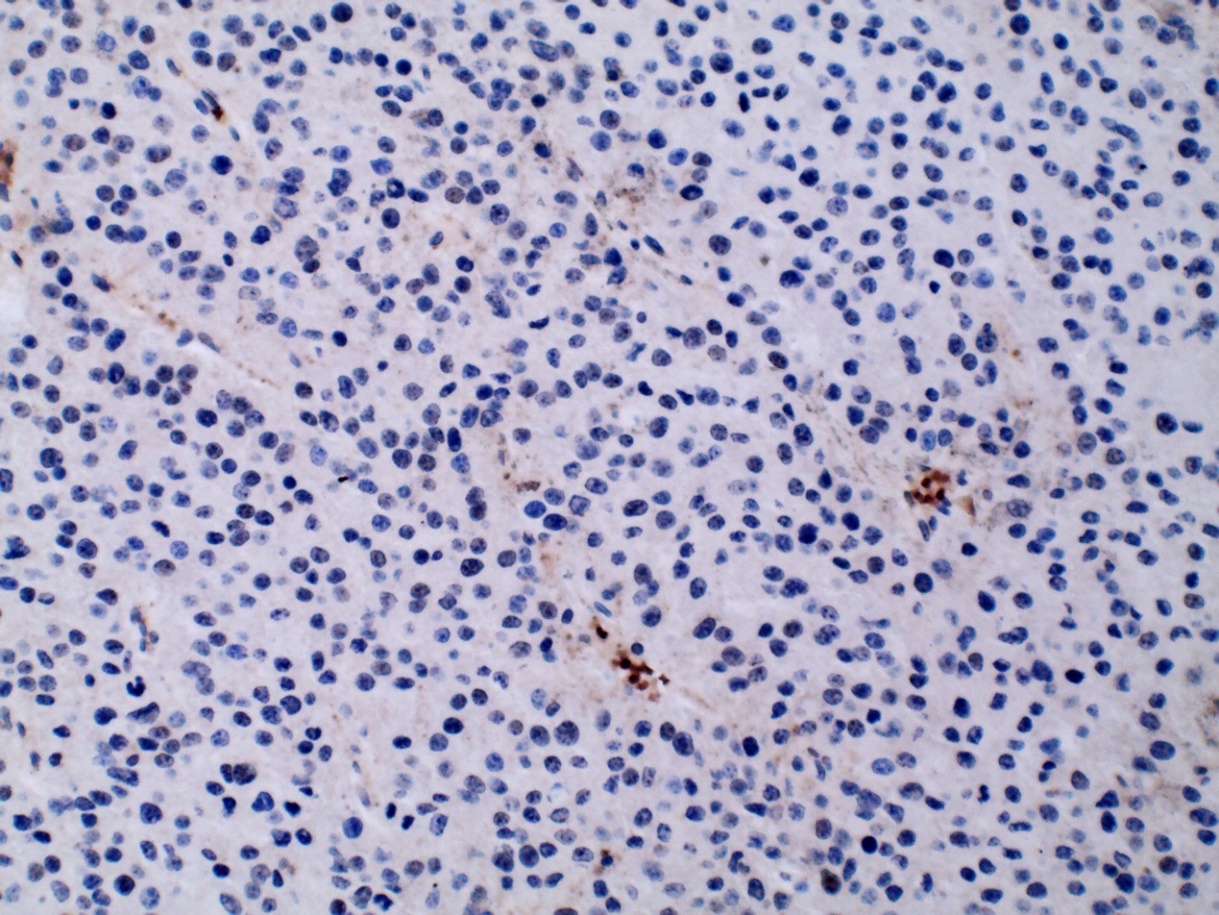


Figure 5 Expression of p53 (Model, ×400)


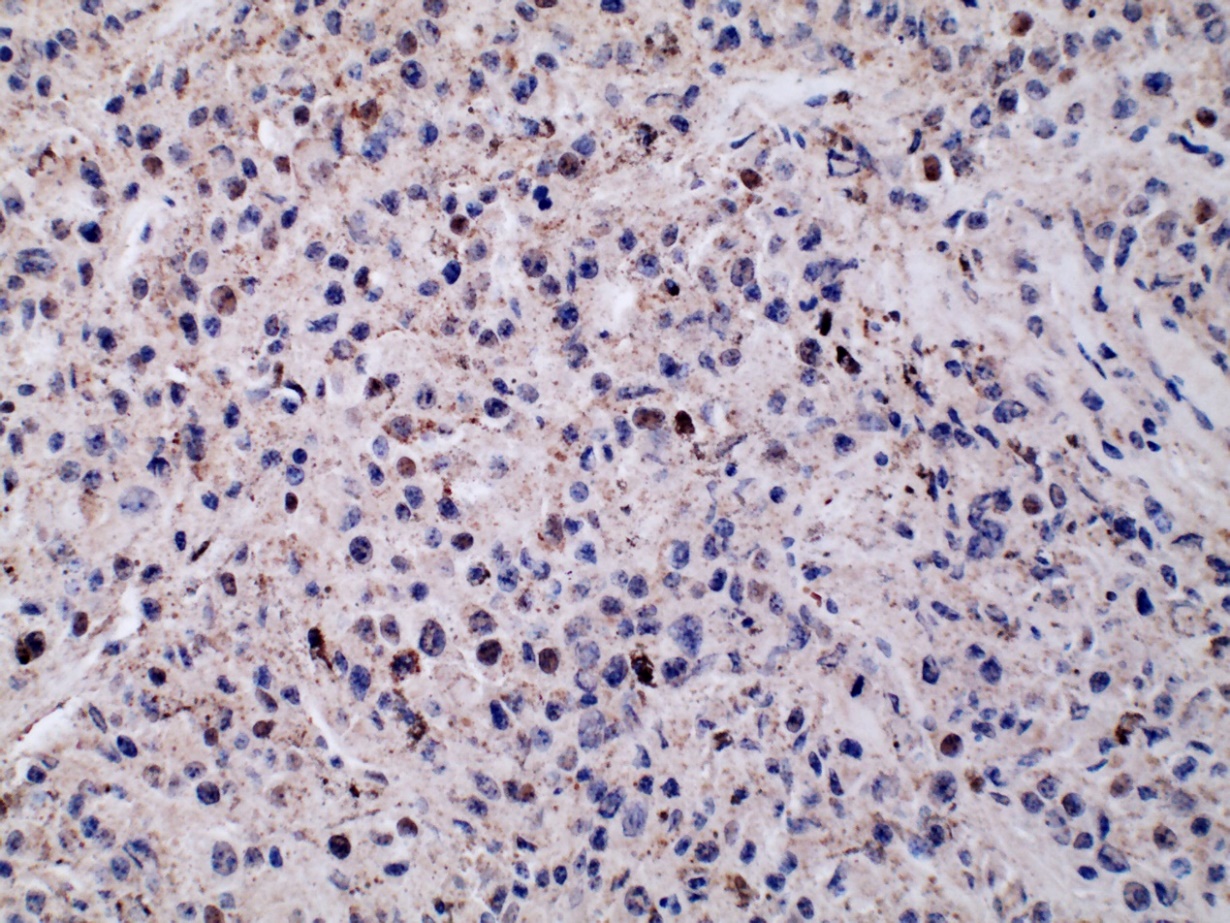


Figure 6 Expression of p53 (DDP, ×400)


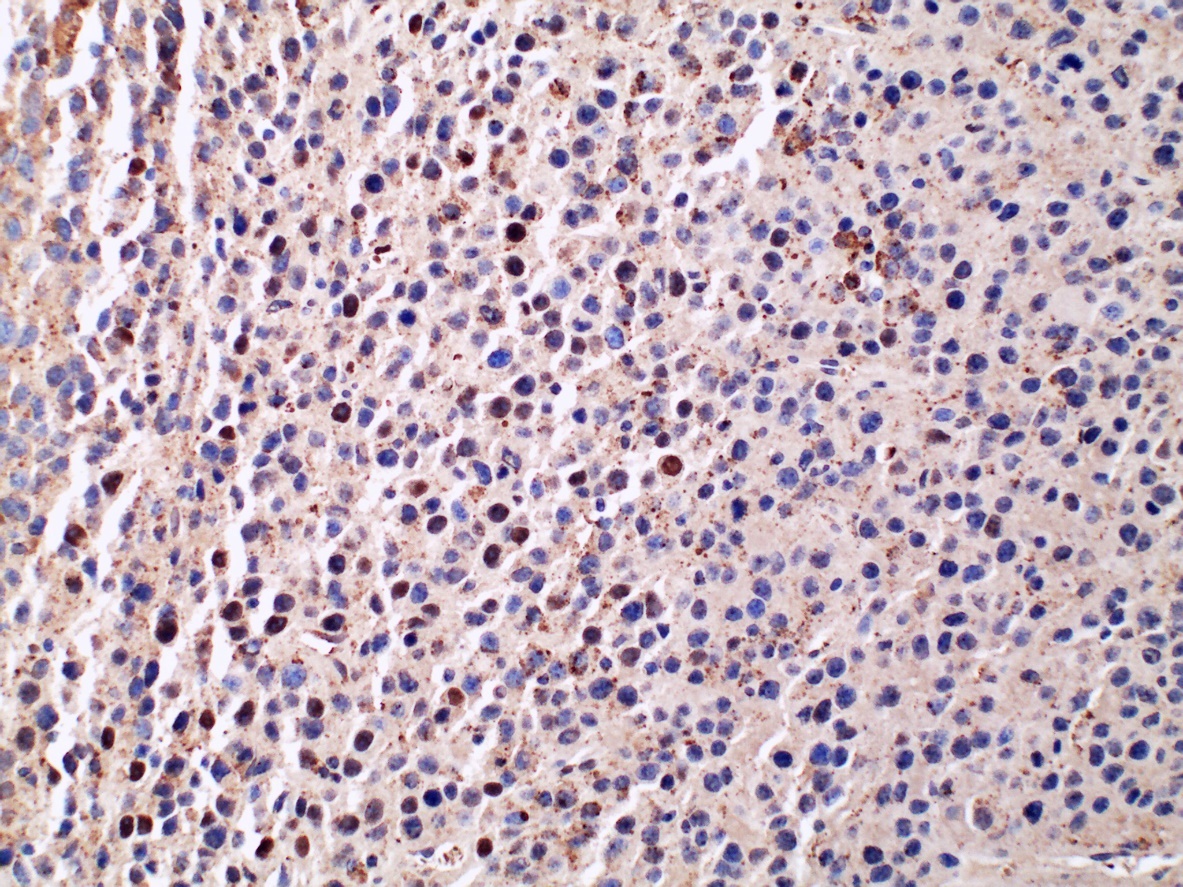


Figure 7 Expression of p53 (ZSPL, ×400)


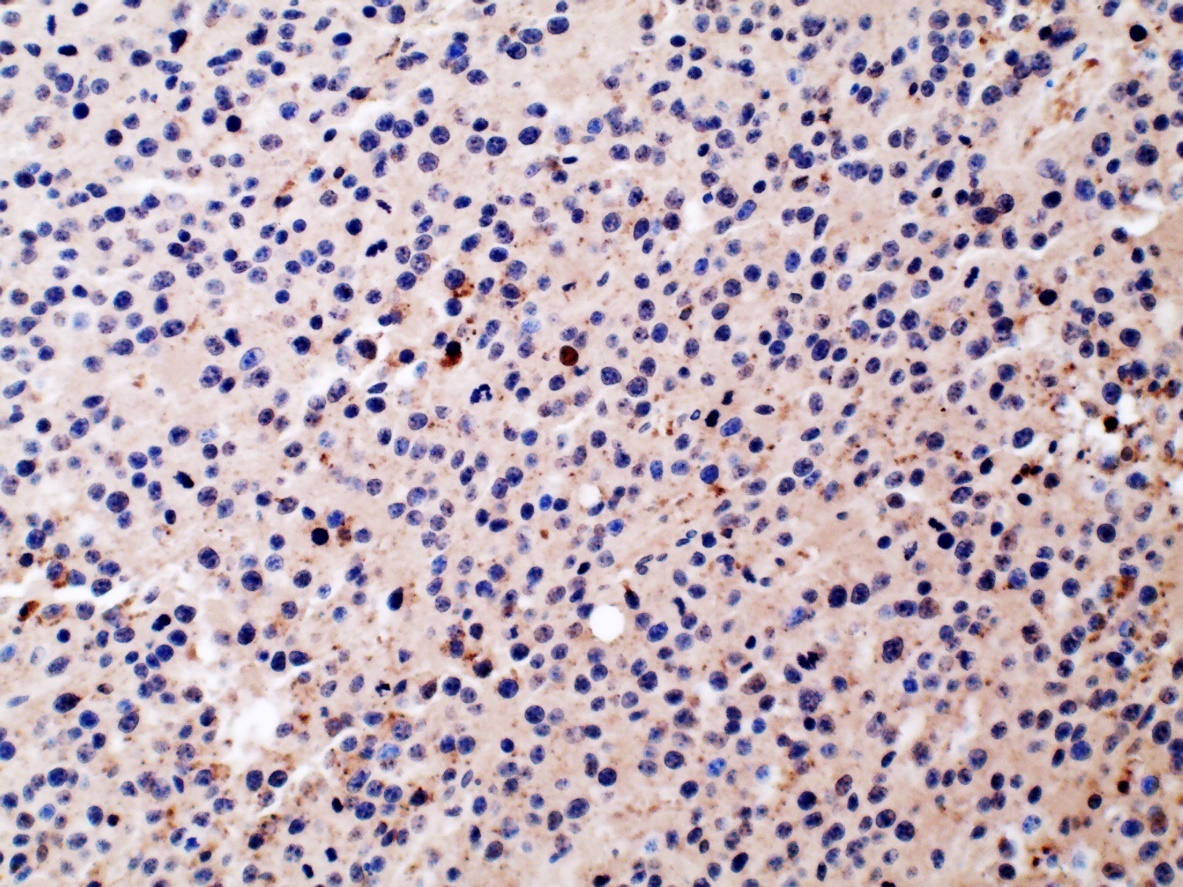


Figure 8 Expression of p53 (ZSPH, ×400)


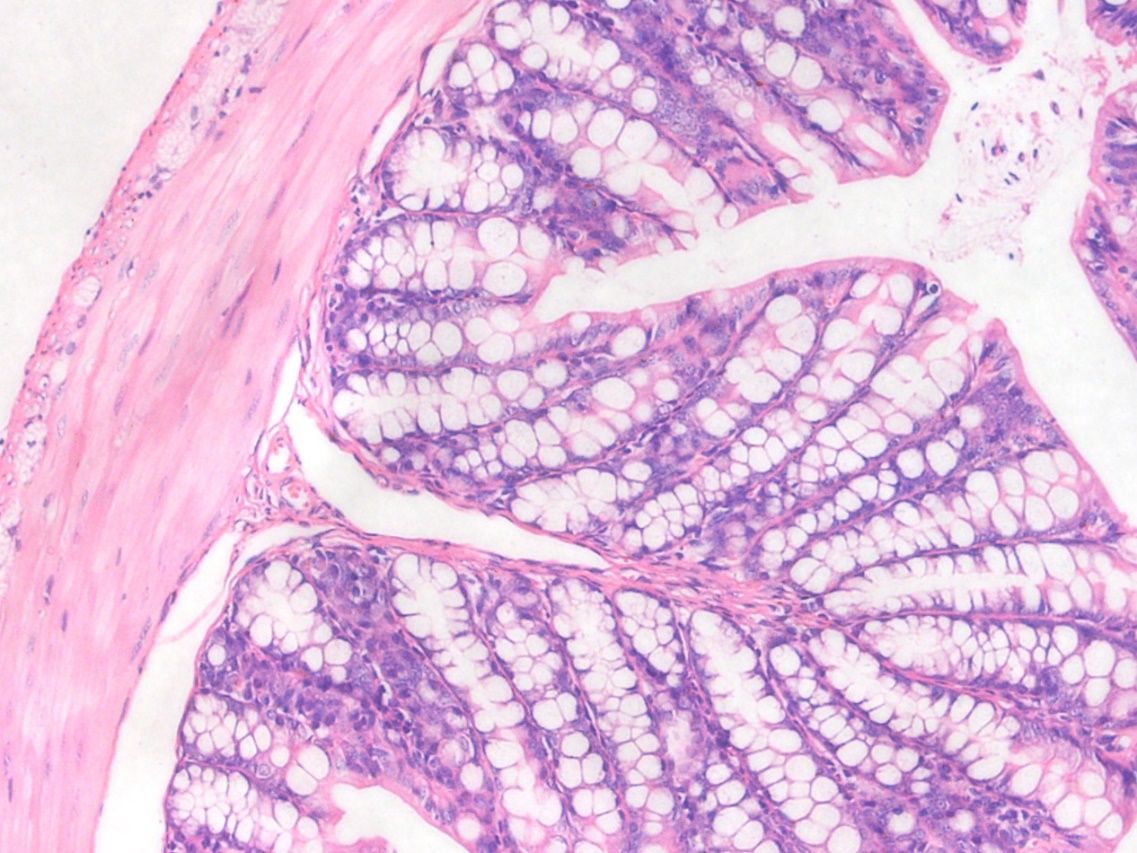


Figure 9 Hematoxylin and eosin staining of colon tissue (Lewis lung cancer mice, Normal, ×400)


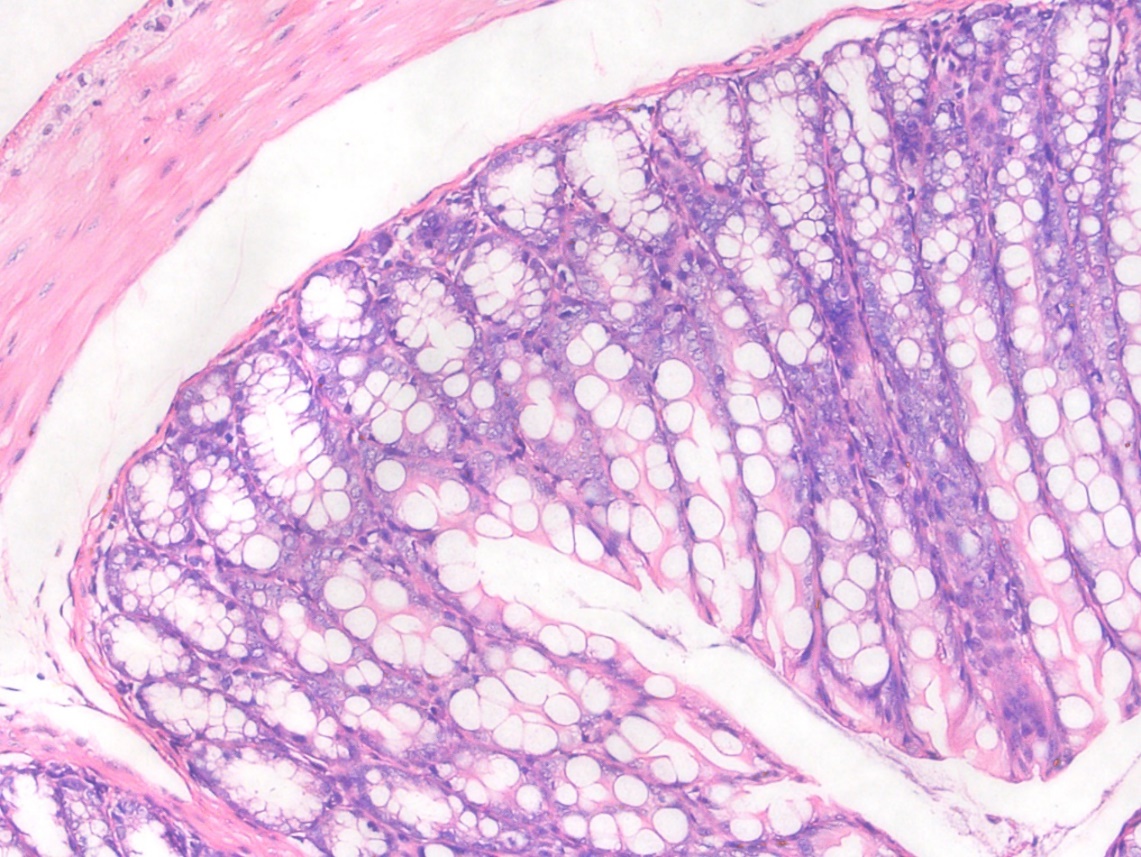


Figure 10 Hematoxylin and eosin staining of colon tissue (Lewis lung cancer mice, Model, ×400)


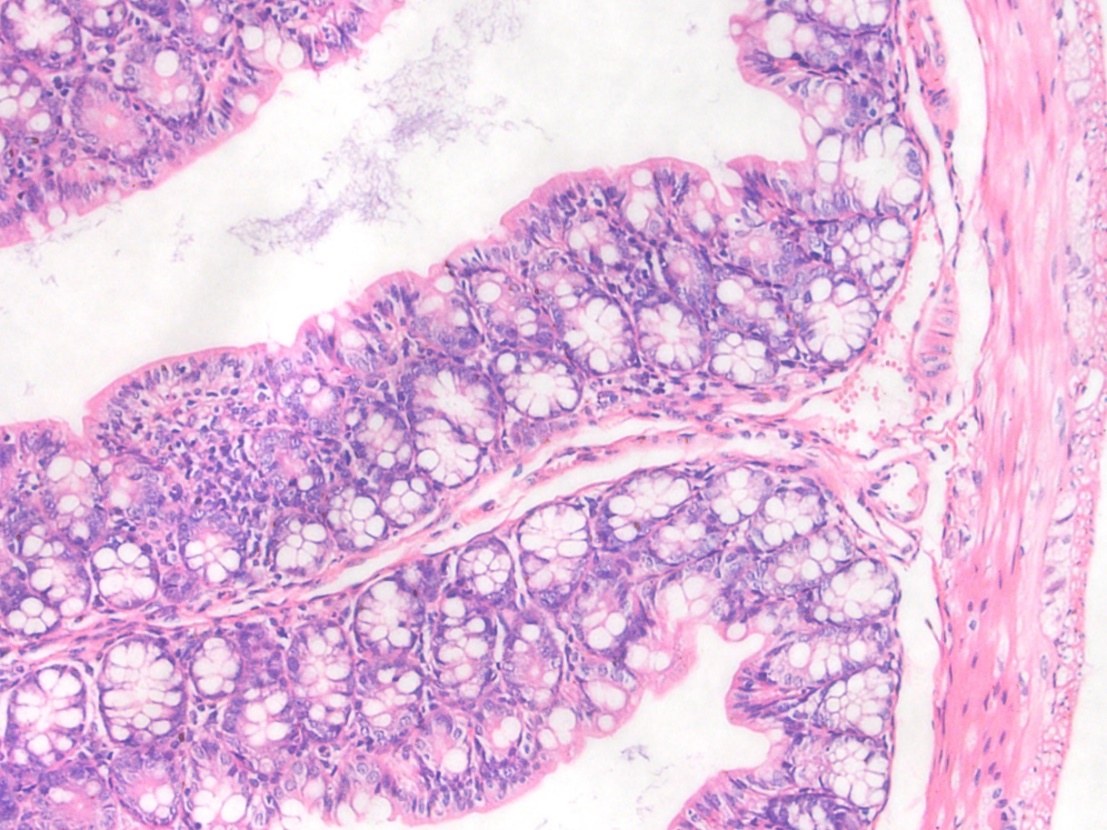


Figure 11 Hematoxylin and eosin staining of colon tissue (Lewis lung cancer mice, ZSPL, ×400)


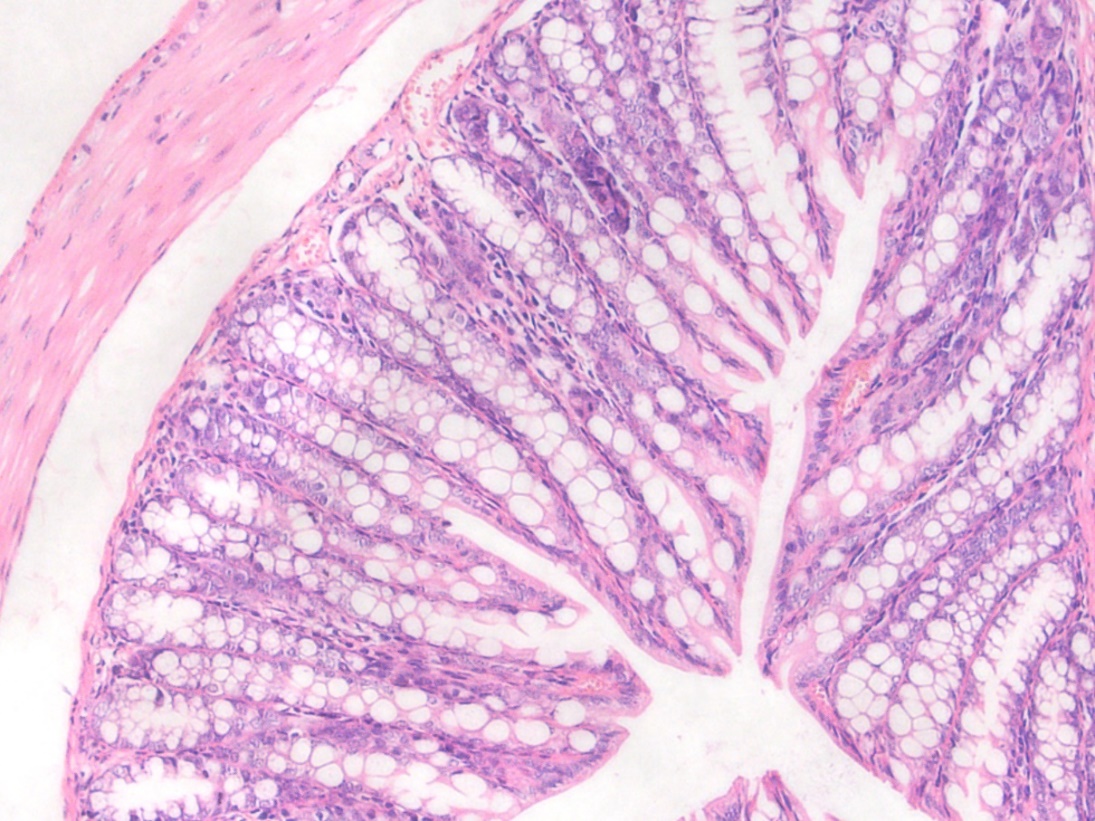


Figure 12 Hematoxylin and eosin staining of colon tissue (Lewis lung cancer mice, ZSPH, ×400)


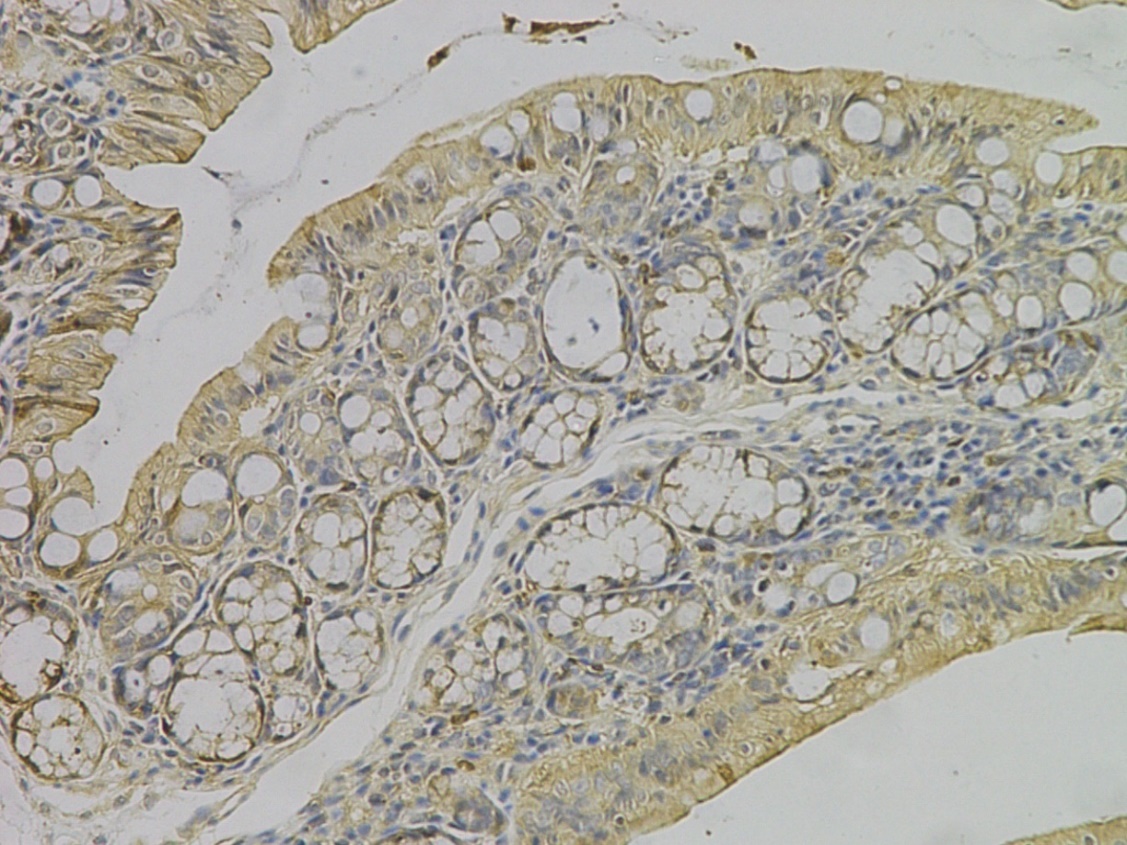


Figure 13 Detection of ZO-1 by immunohistochemical assay (Normal, ×400)


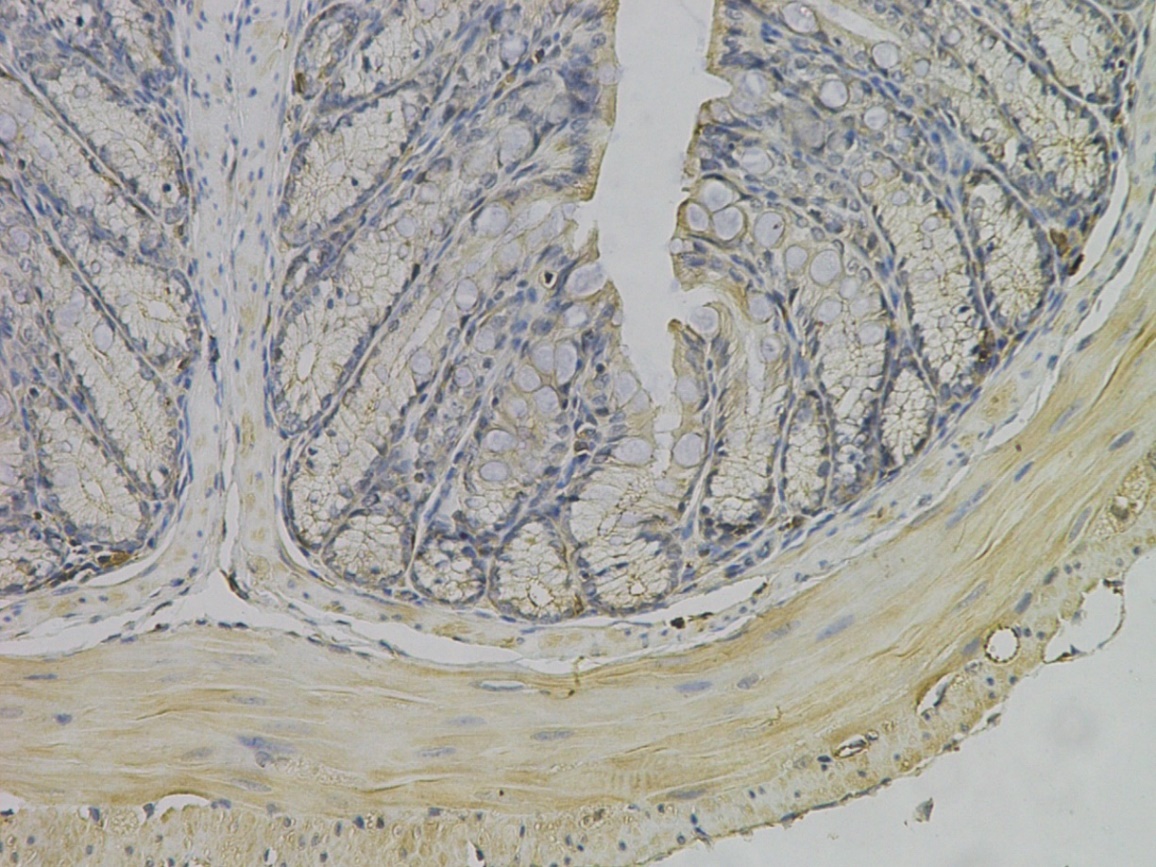


Figure14 Detection of ZO-1 by immunohistochemical assay (Model, ×400)


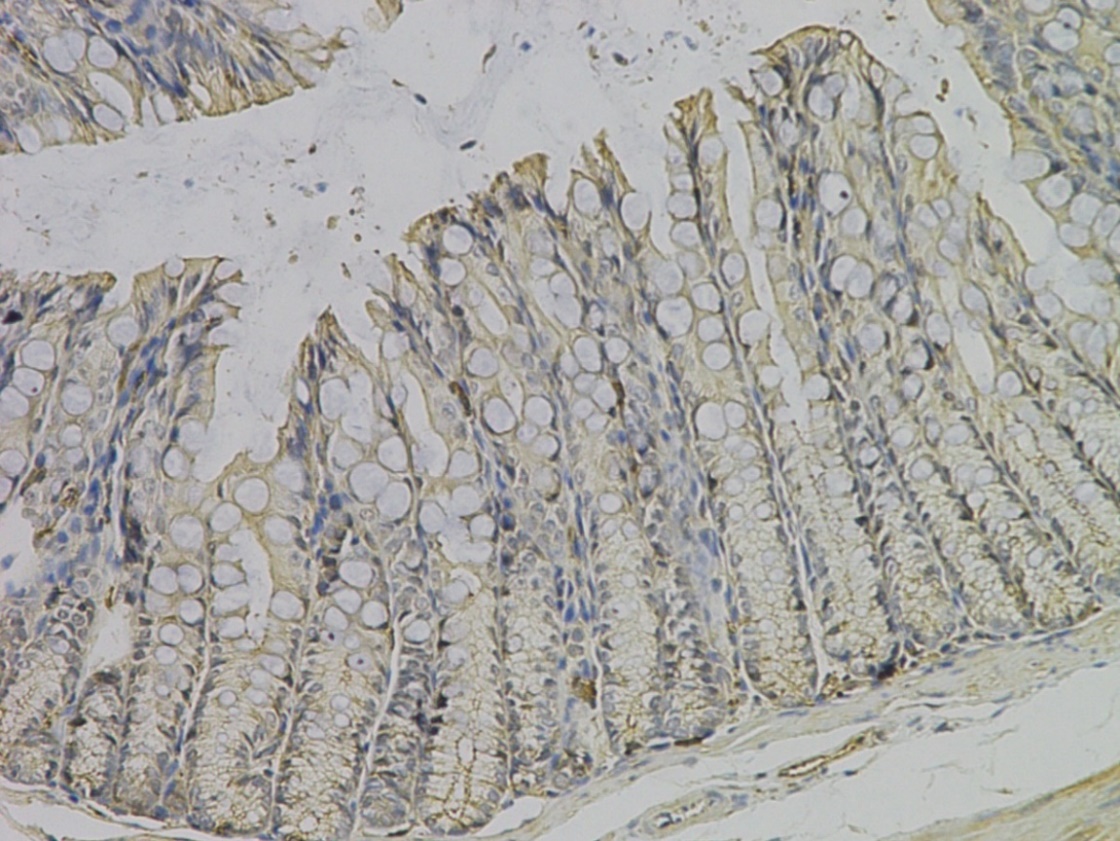


Figure 15 Detection of ZO-1 by immunohistochemical assay (ZSPL, ×400)


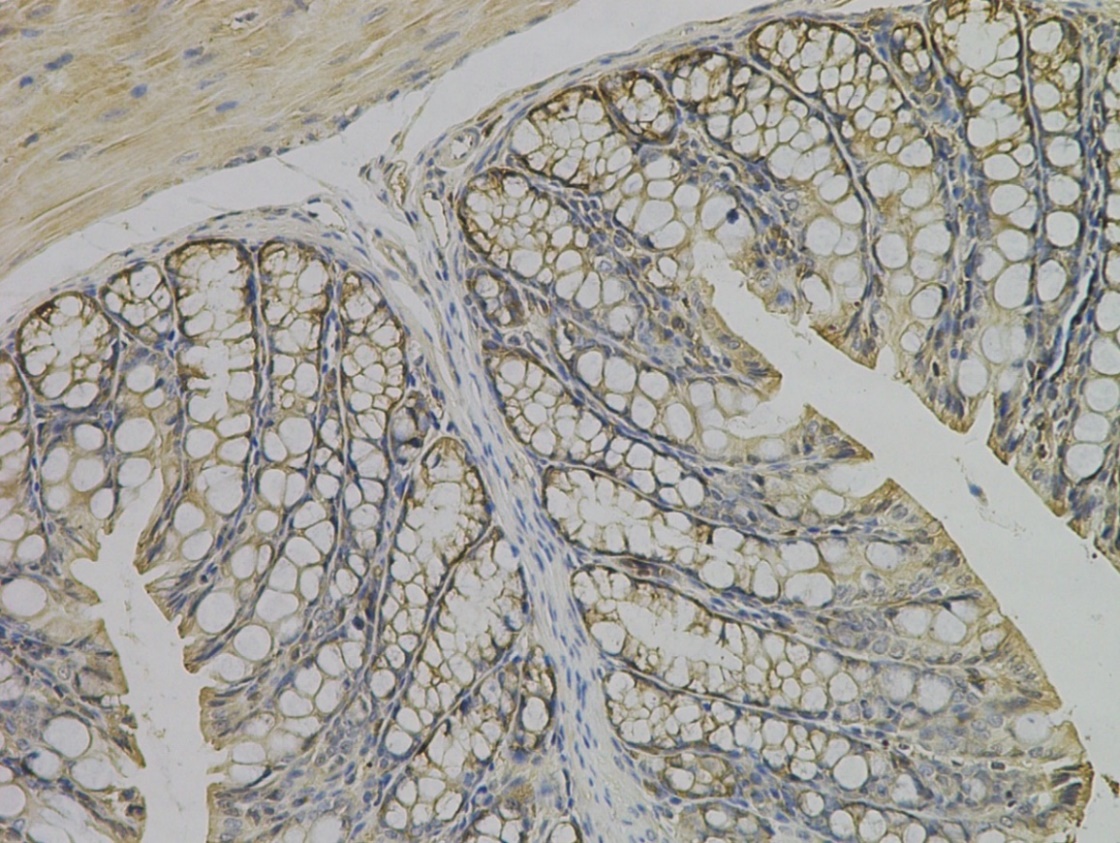


Figure 16 Detection of ZO-1 by immunohistochemical assay (ZSPH, ×400)


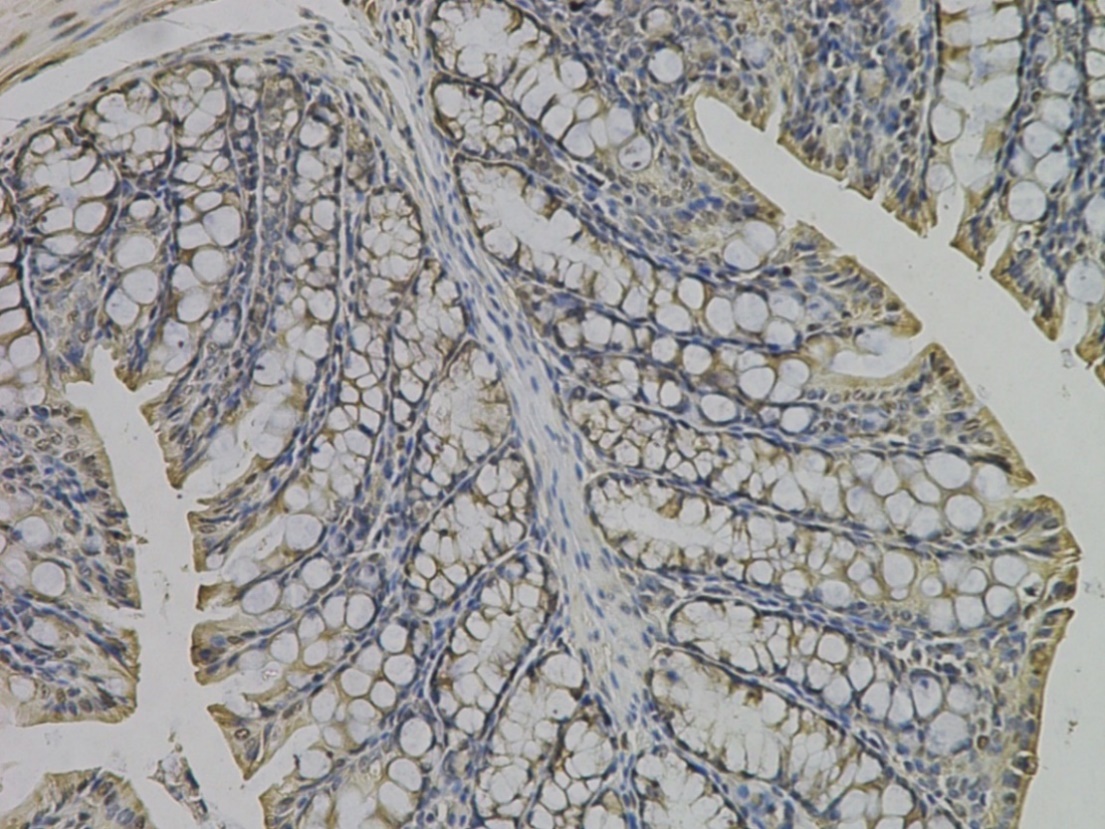


Figure 17 Detection of Occludin by immunohistochemical assay (Normal, ×400)


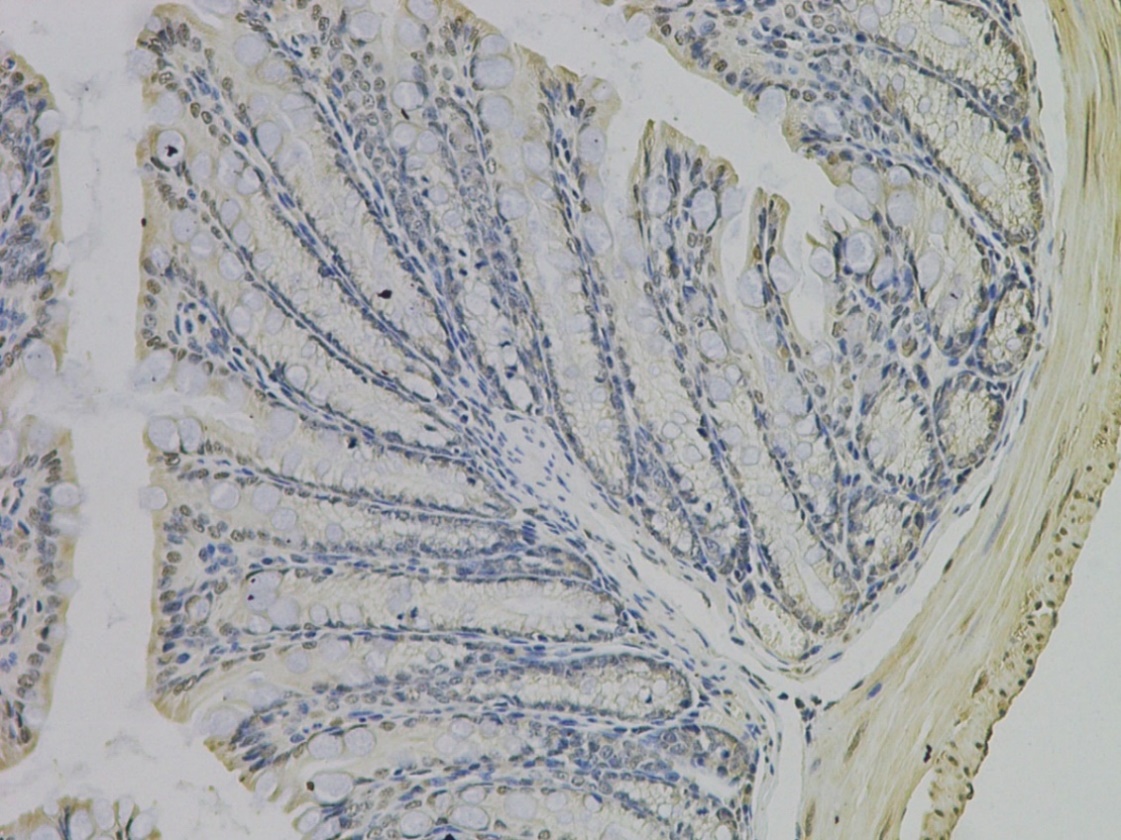


Figure 18 Detection of Occludin by immunohistochemical assay (Model, ×400)


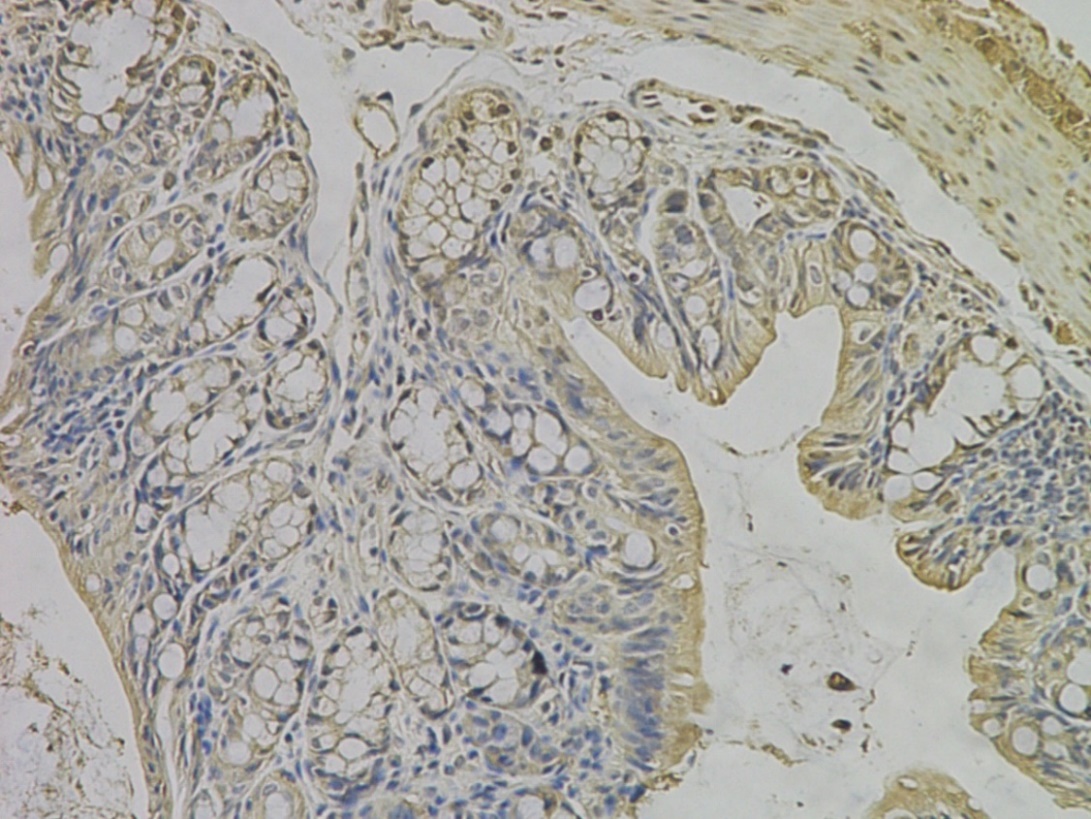


Figure 19 Detection of Occludin by immunohistochemical assay (ZSPL, ×400)


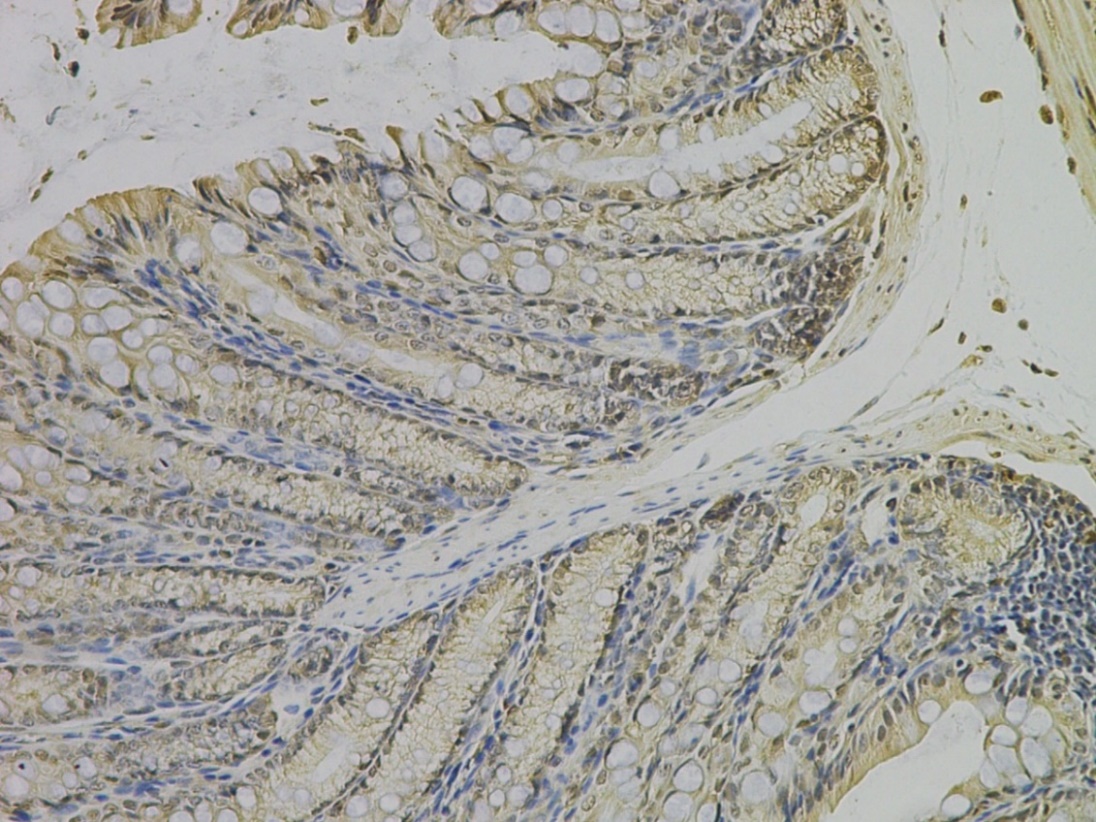


Figure 20 Detection of Occludin by immunohistochemical assay (ZSPH, ×400)


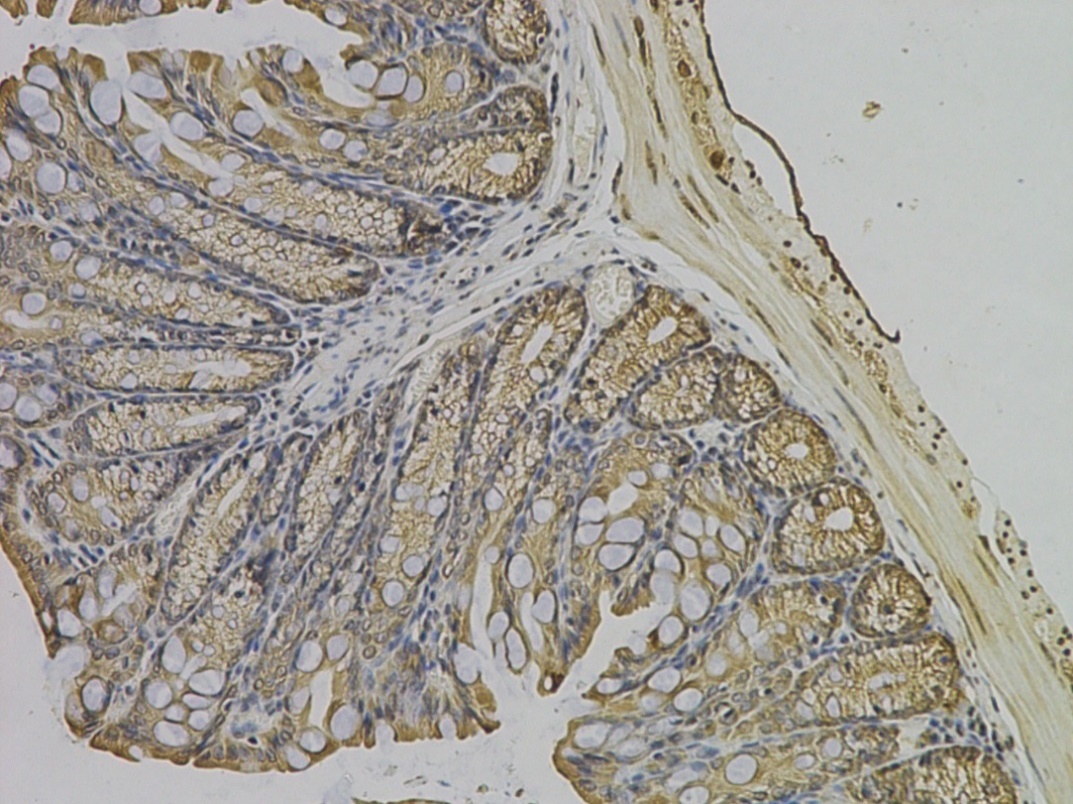


Figure 21 Detection of Claudin-1 by immunohistochemical assay(Normal, ×400)


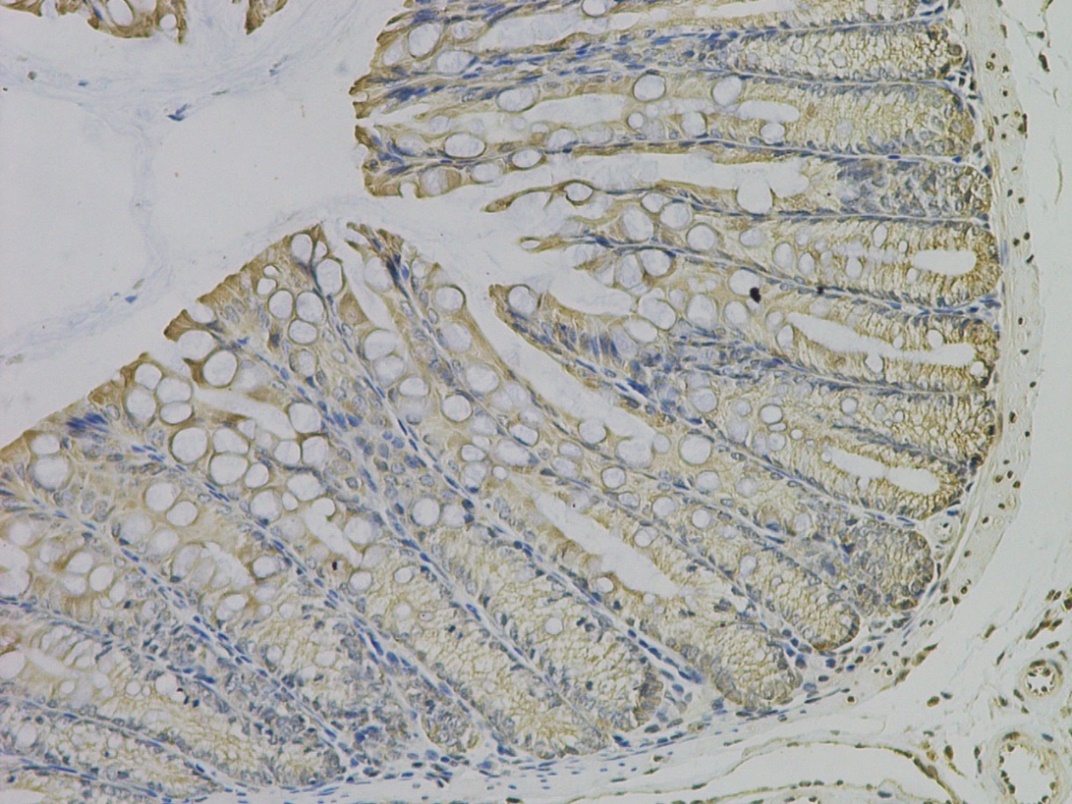


Figure 22 Detection of Claudin-1 by immunohistochemical assay(Model, ×400)


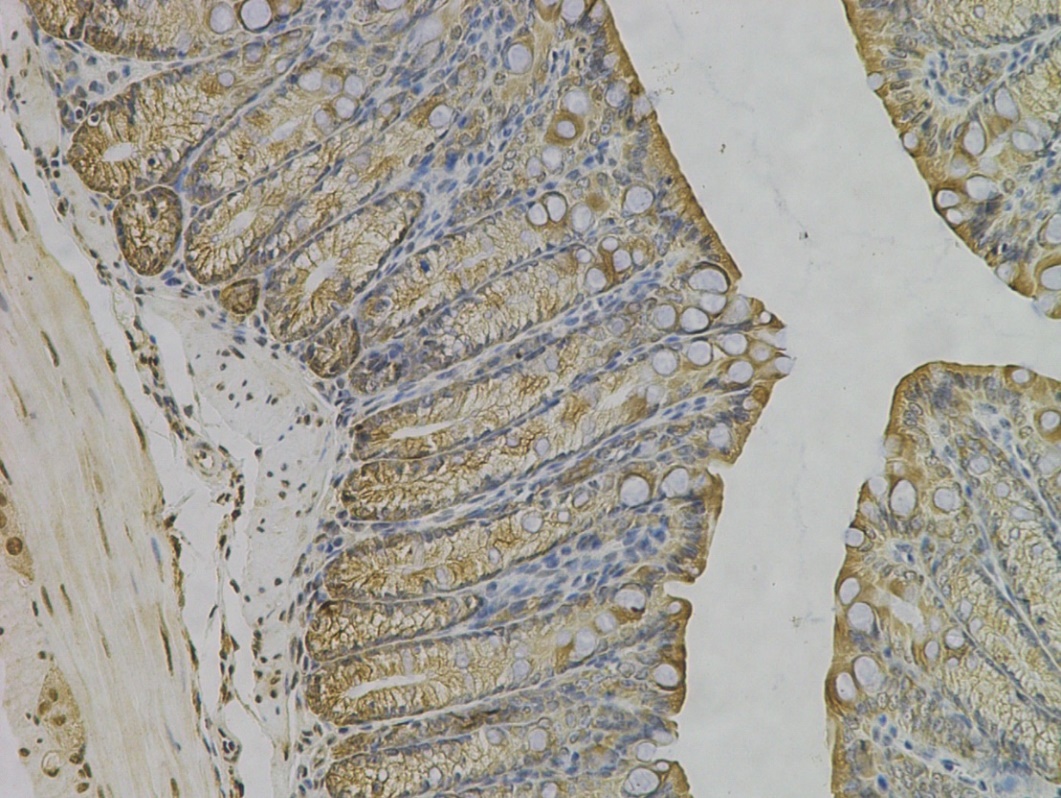


Figure 23 Detection of Claudin-1 by immunohistochemical assay(ZSPL, ×400)


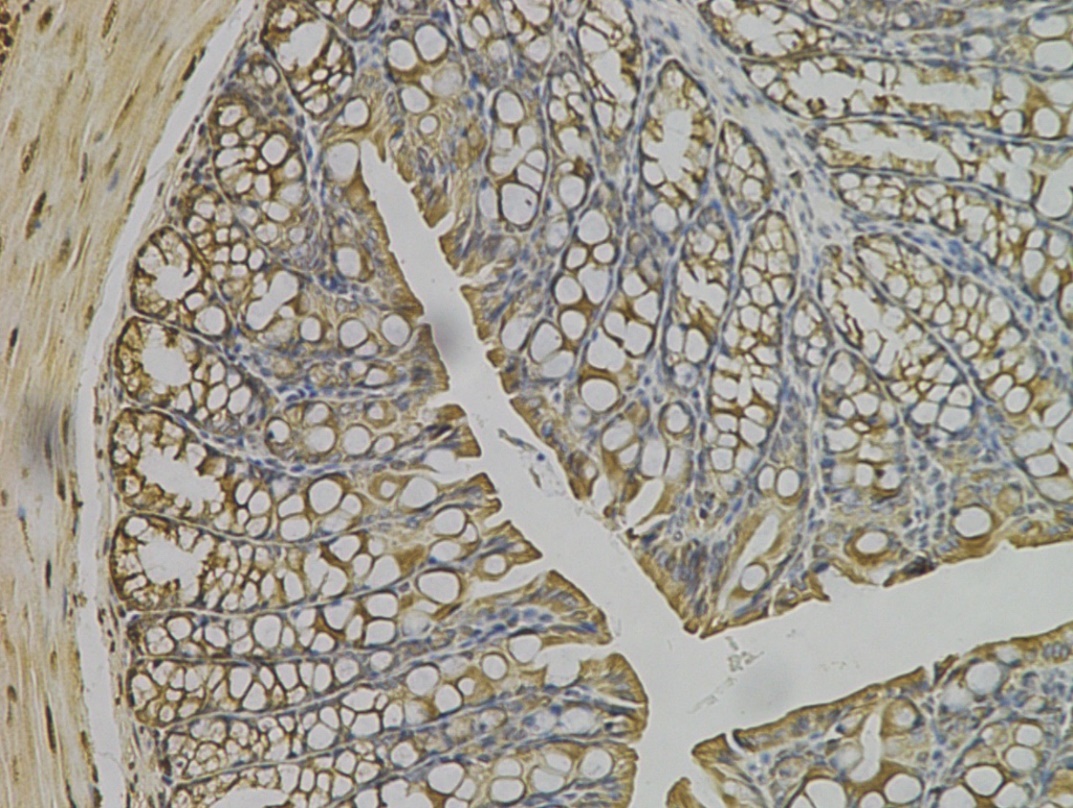


Figure 24 Detection of Claudin-1 by immunohistochemical assay(ZSPH, ×400)


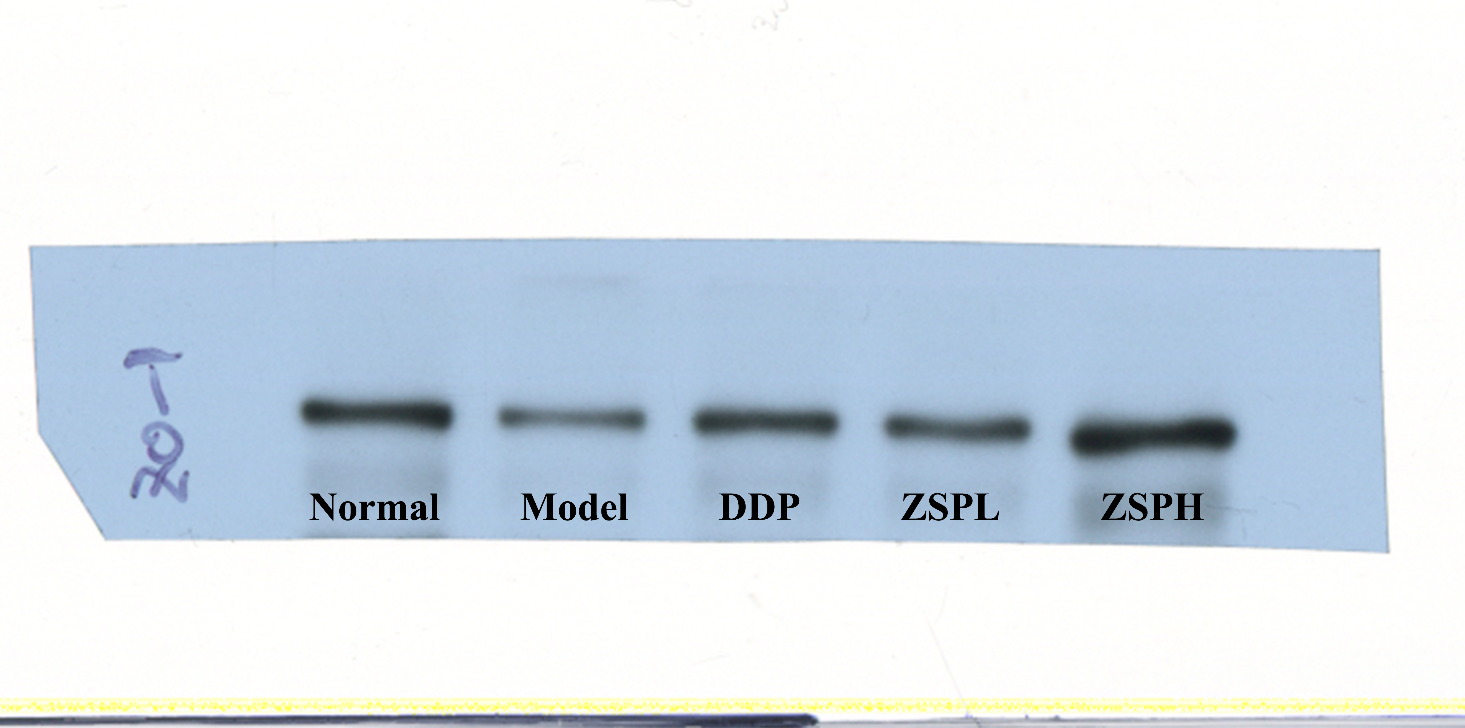


Figure 25 Western blot original stripe (ZO-1)


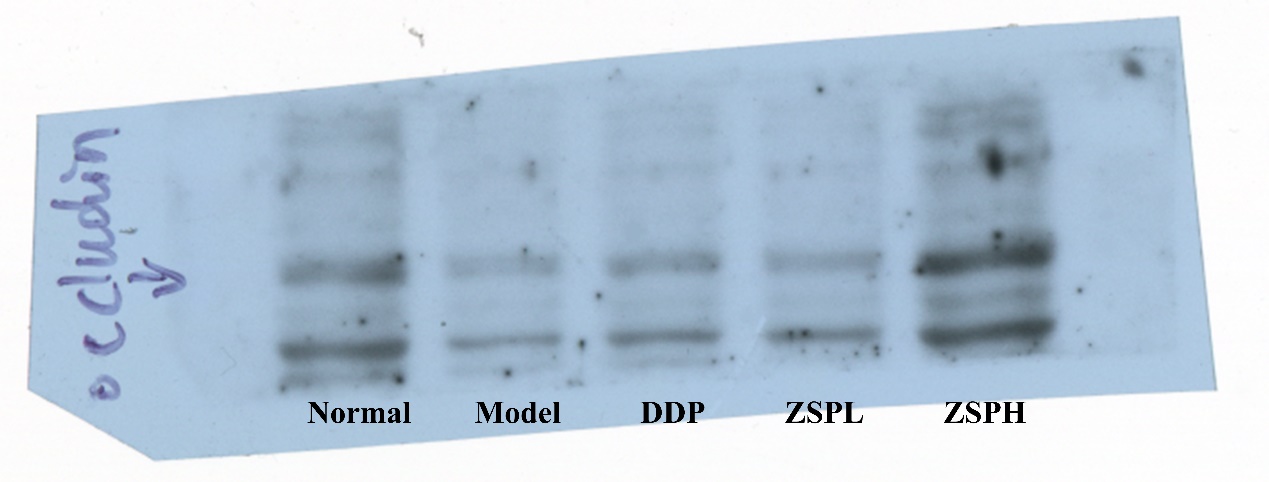


Figure 26 Western blot original stripe (occludin)


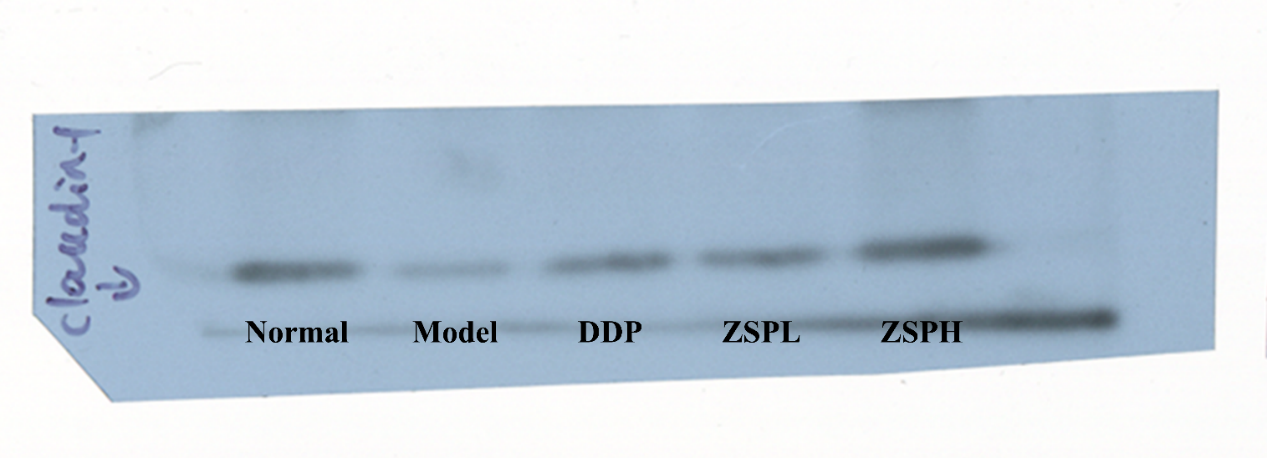


Figure 27 Western blot original stripe (Claudin-1)


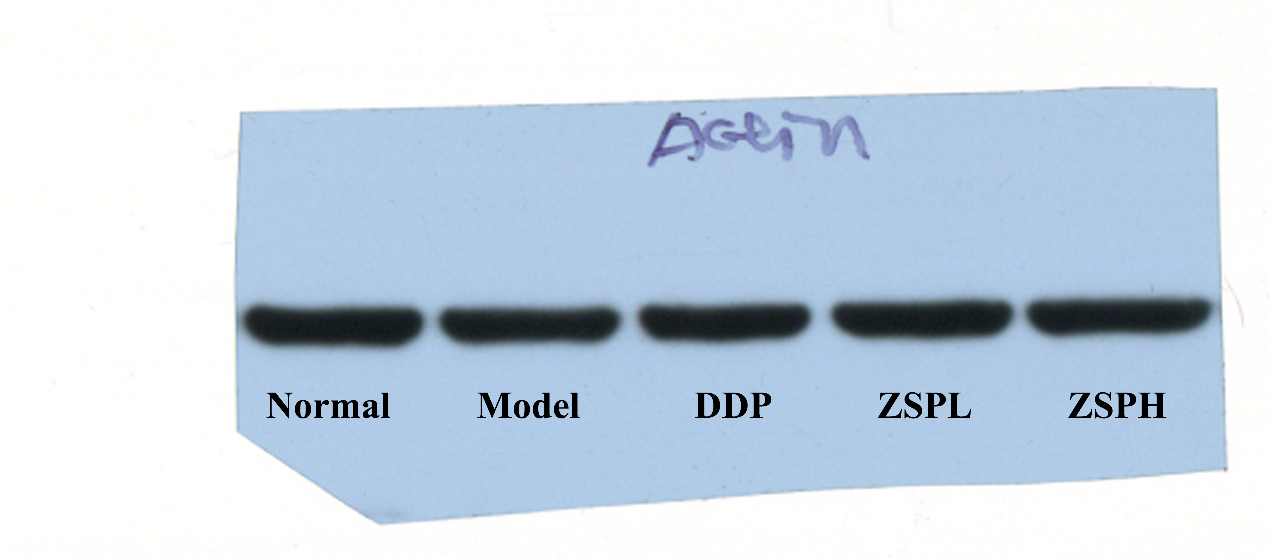


Figure 28 Western blot original stripe (Actin)

**Note: The DDP group was not discussed in this experiment, so the picture in the manuscript was cut.**


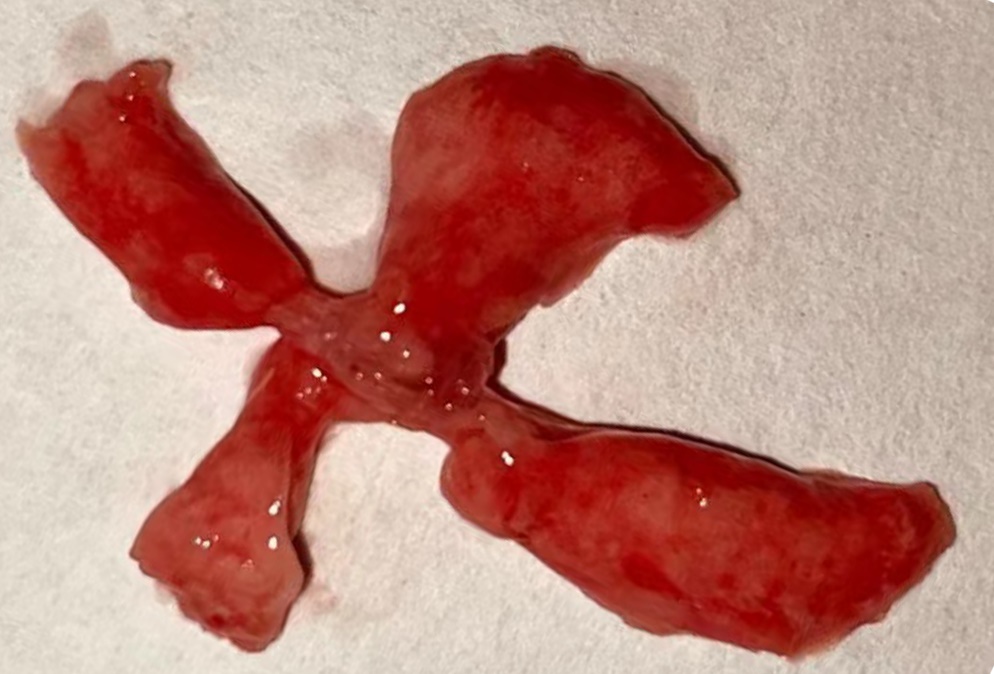


Figure 29 The whole lung by the naked eye (Urethane-induced lung cancer mice, Normal)


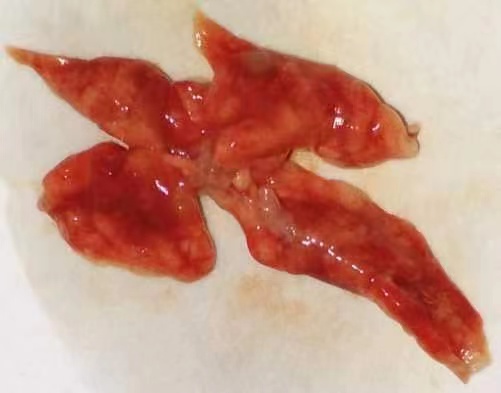


Figure 30 The whole lung by the naked eye (Urethane-induced lung cancer mice, Model)


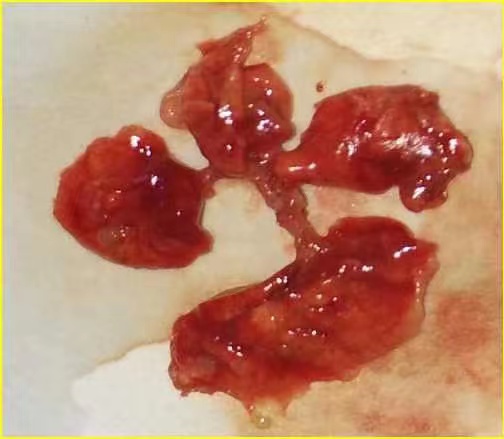


Figure 31 The whole lung by the naked eye (Urethane-induced lung cancer mice, ZSPL)


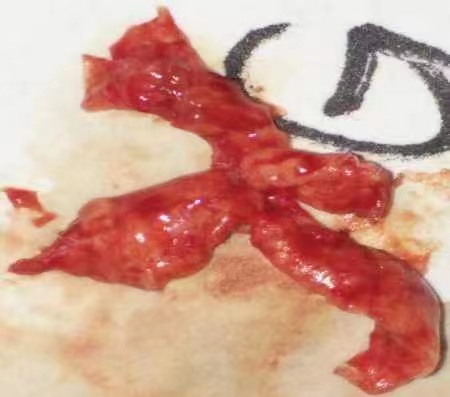


Figure 32 The whole lung by the naked eye (Urethane-induced lung cancer mice, ZSPH)


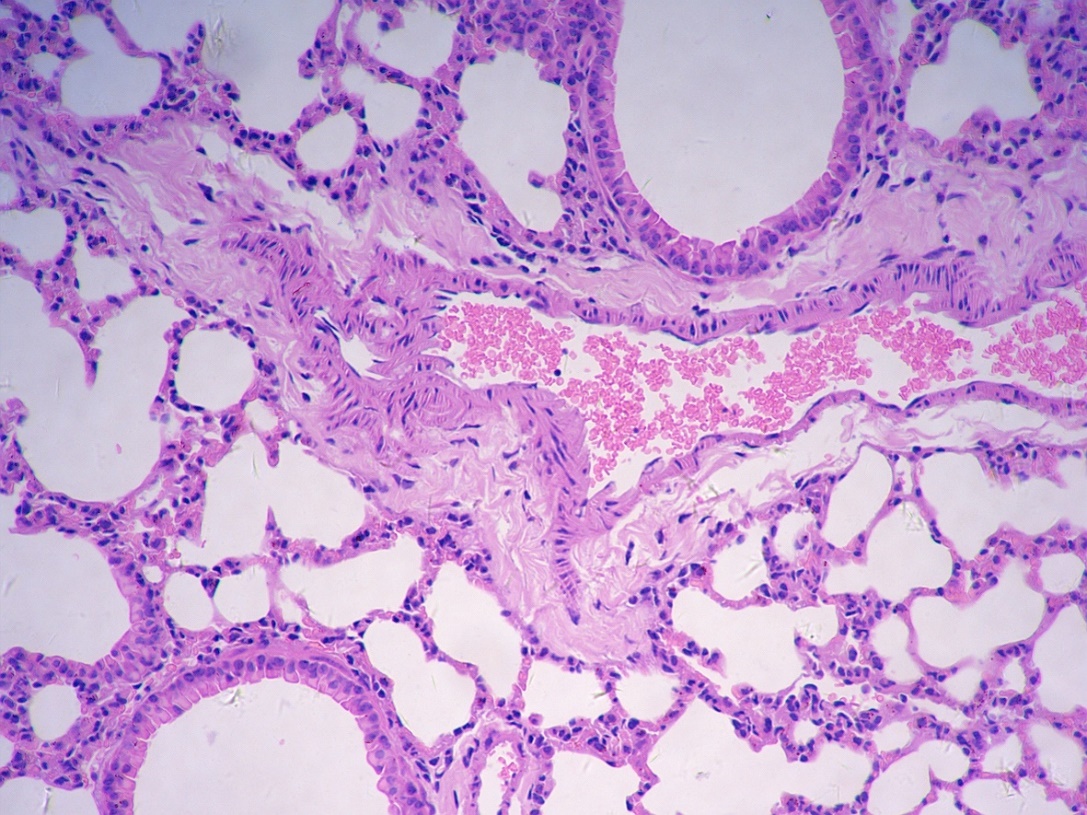


Figure 33 H&E staining of lung tissue (Urethane-induced lung cancer mice, Normal, ×200)


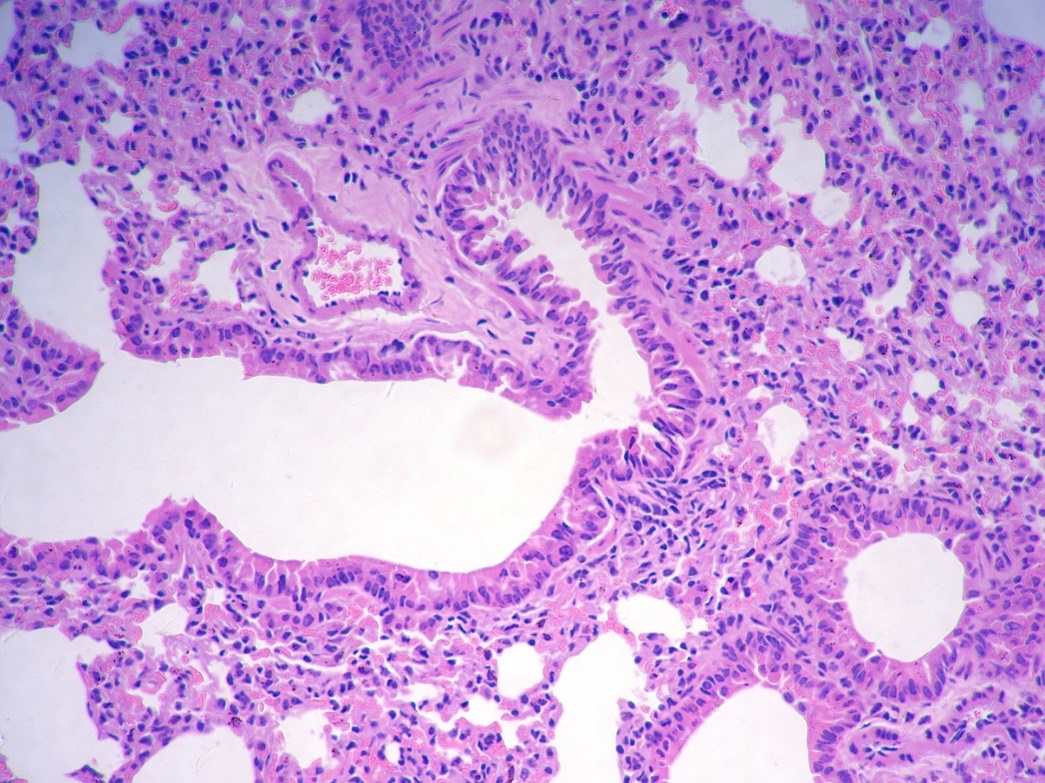


Figure 34 H&E staining of lung tissue (Urethane-induced lung cancer mice, Model, ×200)


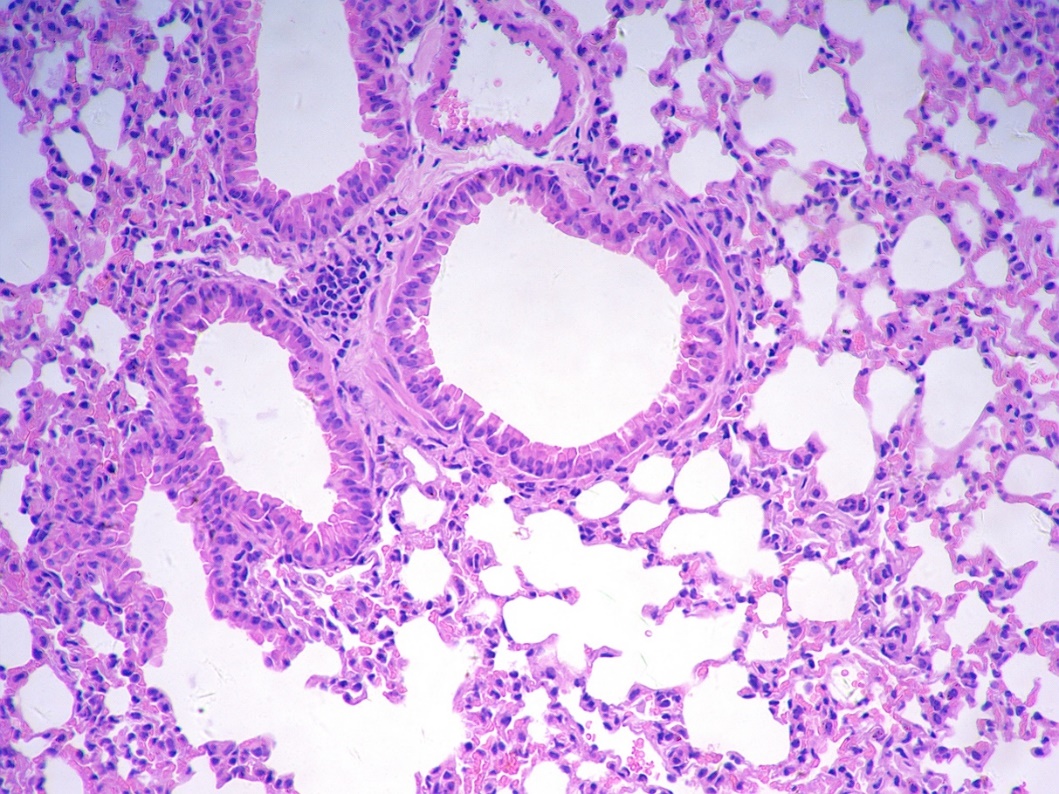


Figure 35 H&E staining of lung tissue (Urethane-induced lung cancer mice, ZSPL, ×200)


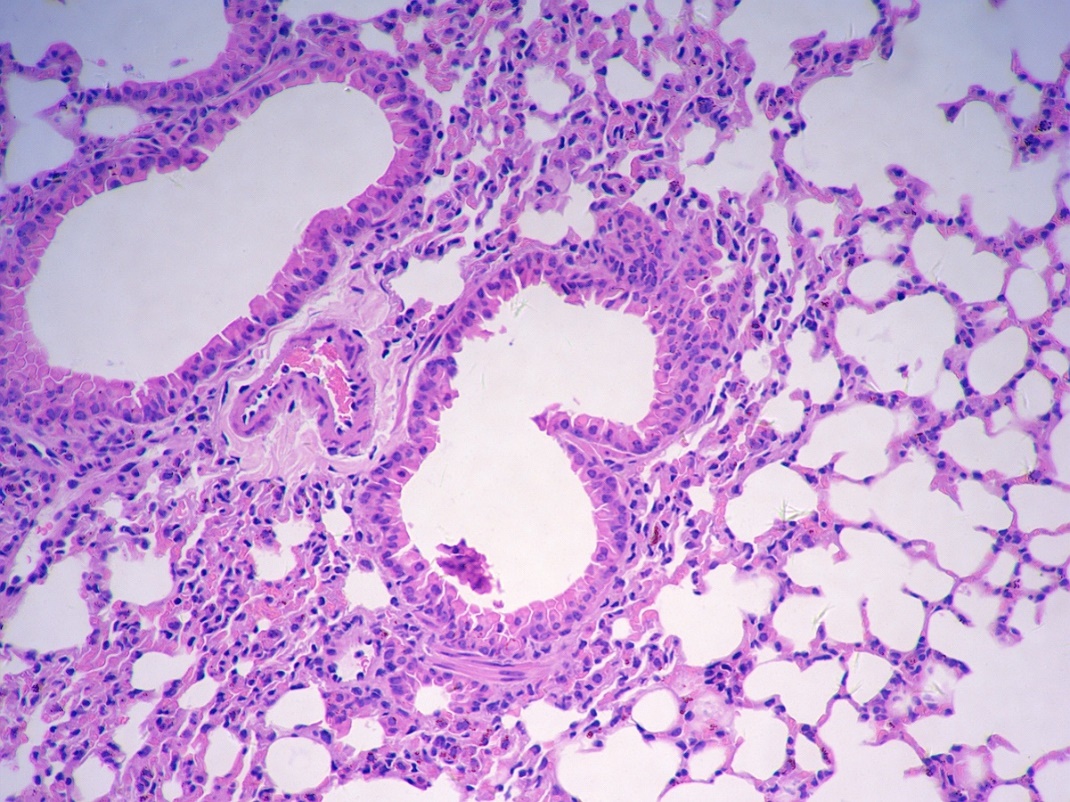


Figure 36 H&E staining of lung tissue (Urethane-induced lung cancer mice, ZSPH, ×200)


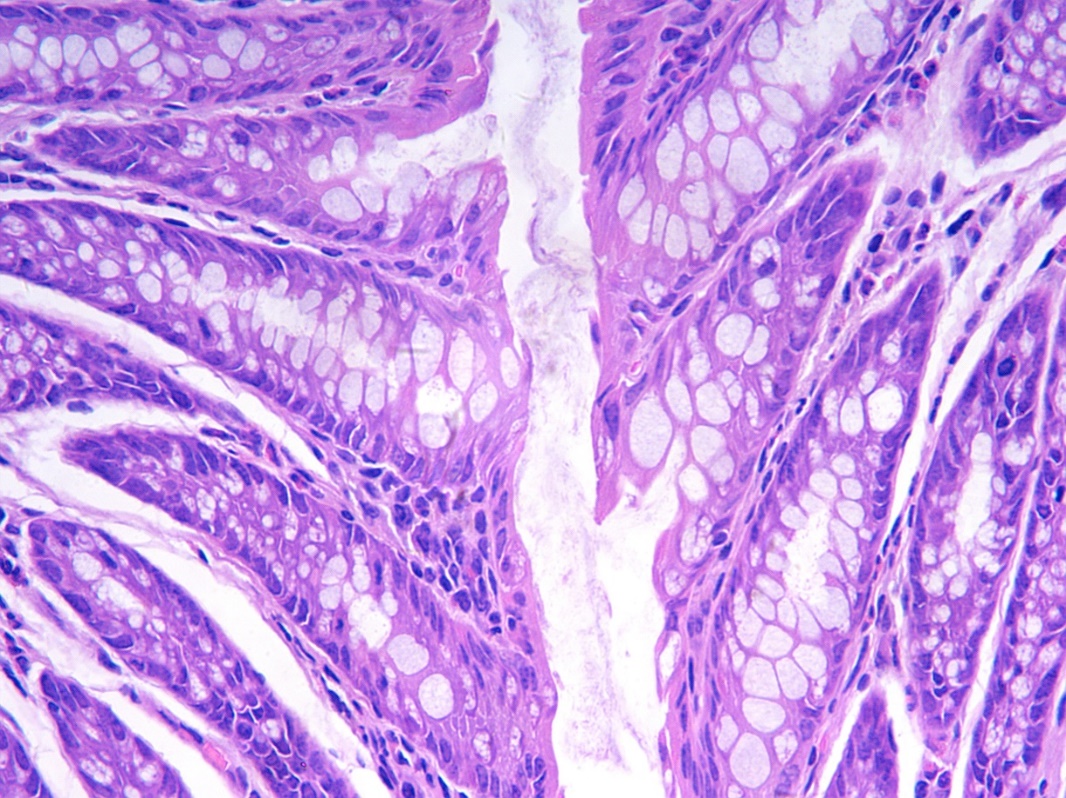


Figure 37 H&E staining of colon tissue (Urethane-induced lung cancer mice, Normal, ×200)


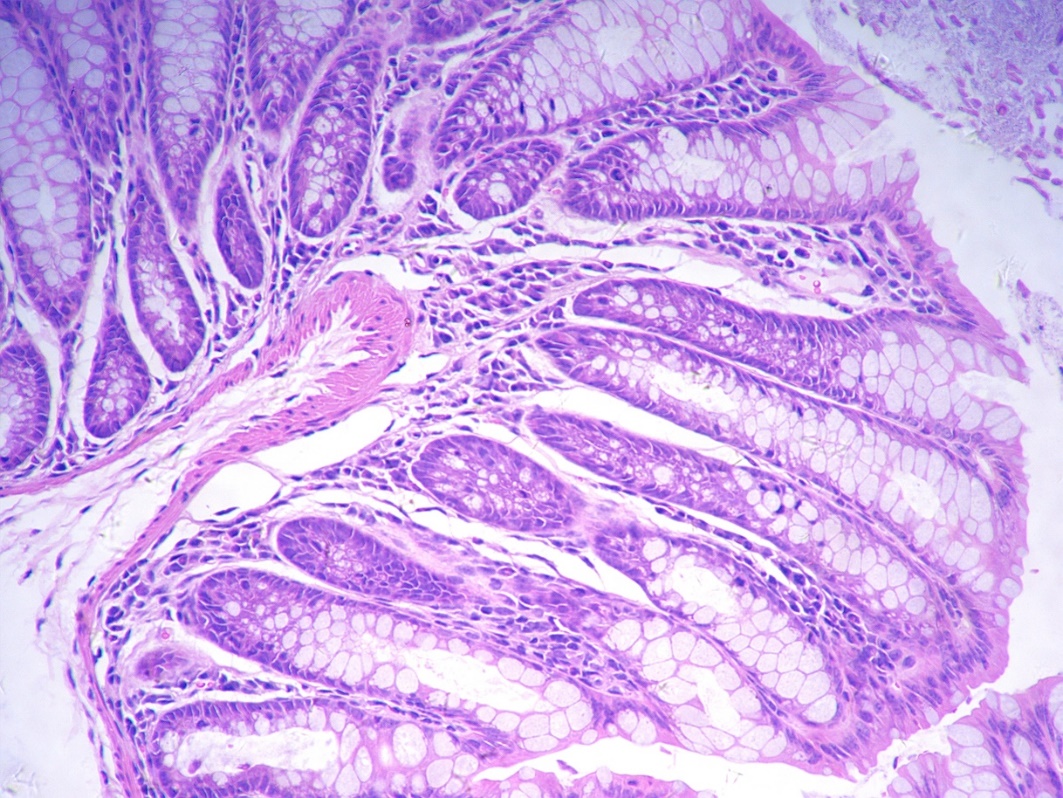


Figure 38 H&E staining of colon tissue (Urethane-induced lung cancer mice, Model, ×200)


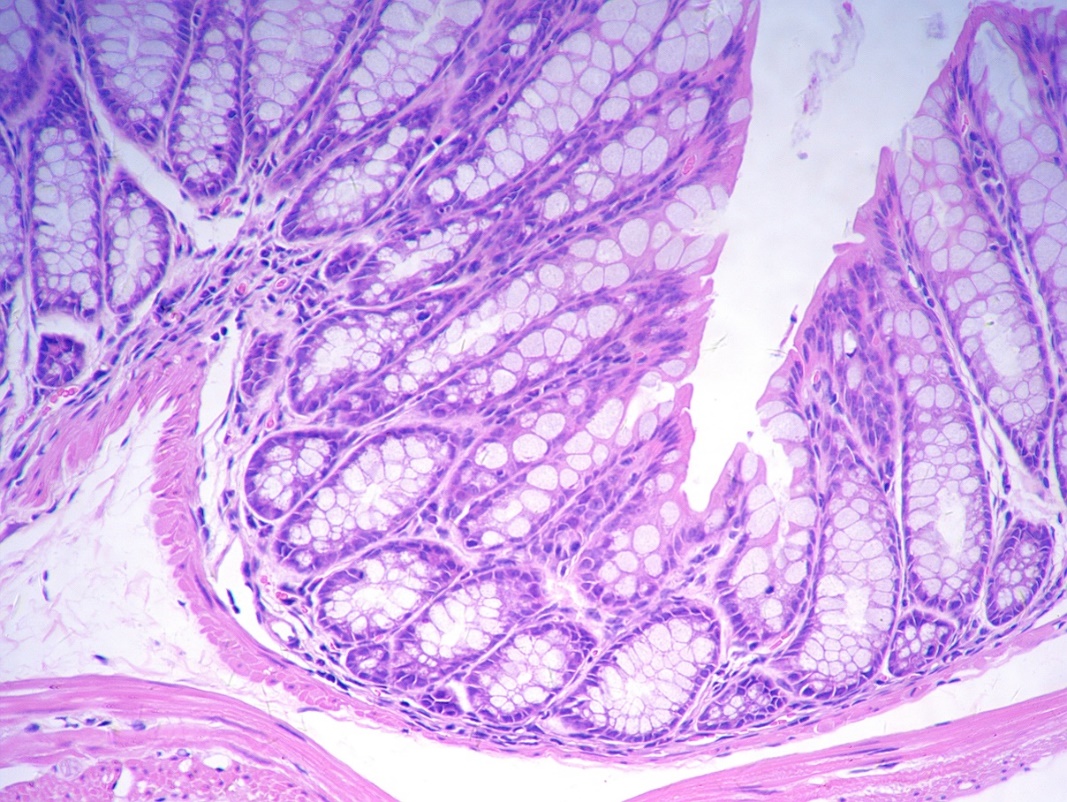


Figure 39 H&E staining of colon tissue (Urethane-induced lung cancer mice, ZSPL, ×200)


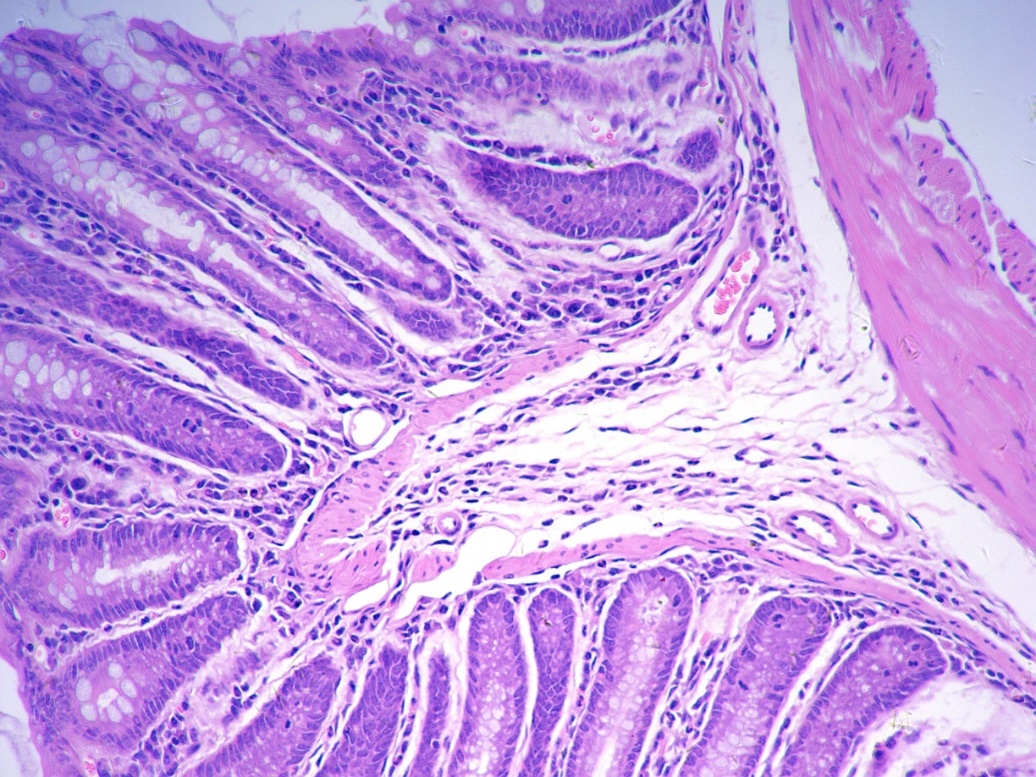


Figure 40 H&E staining of colon tissue (Urethane-induced lung cancer mice, ZSPH, ×200)
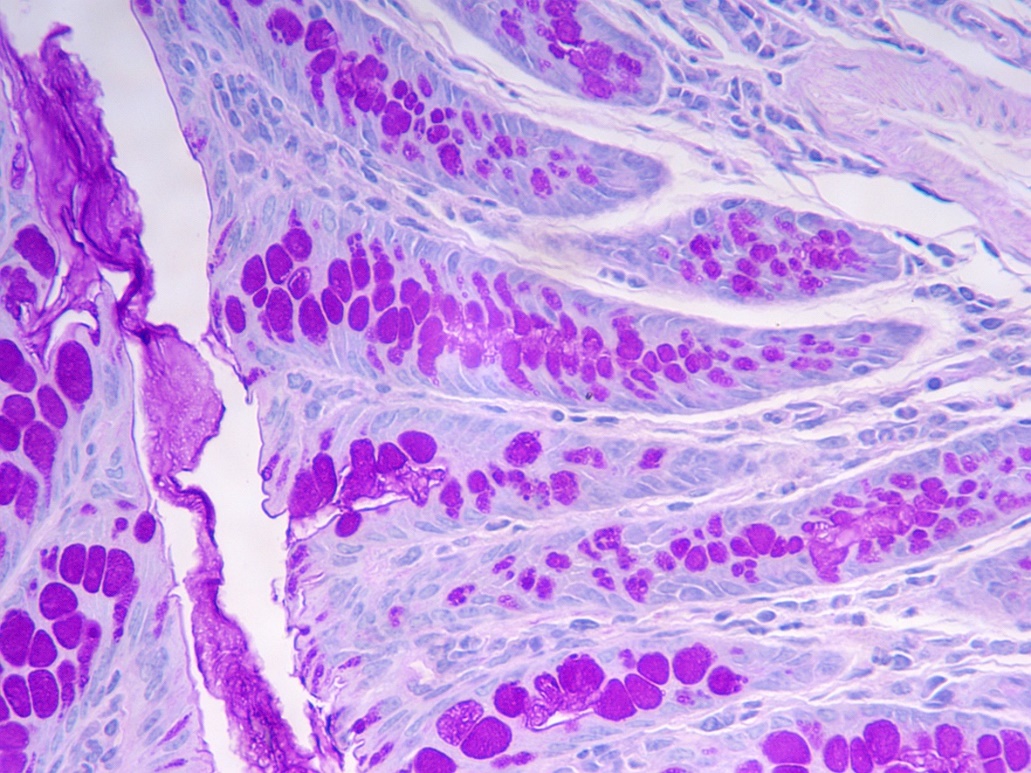


Figure 41 Periodic acid–Schiff staining of colon (Normal, ×200)


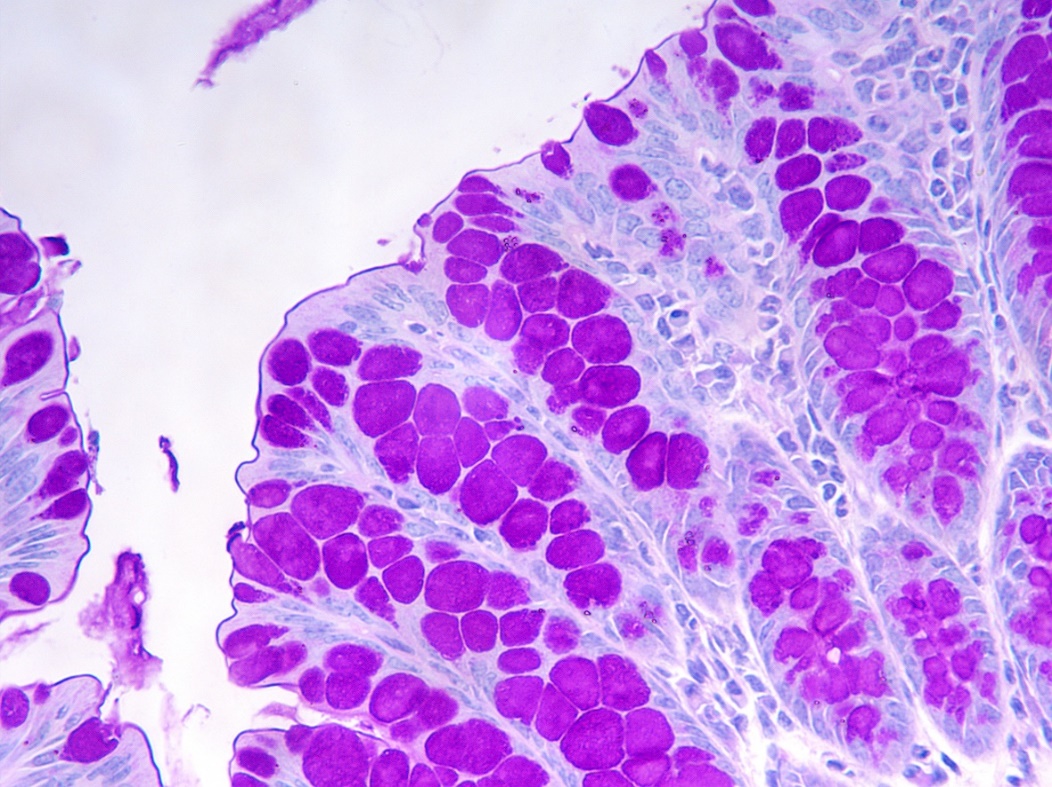


Figure 42 Periodic acid–Schiff staining of colon (Model, ×200)


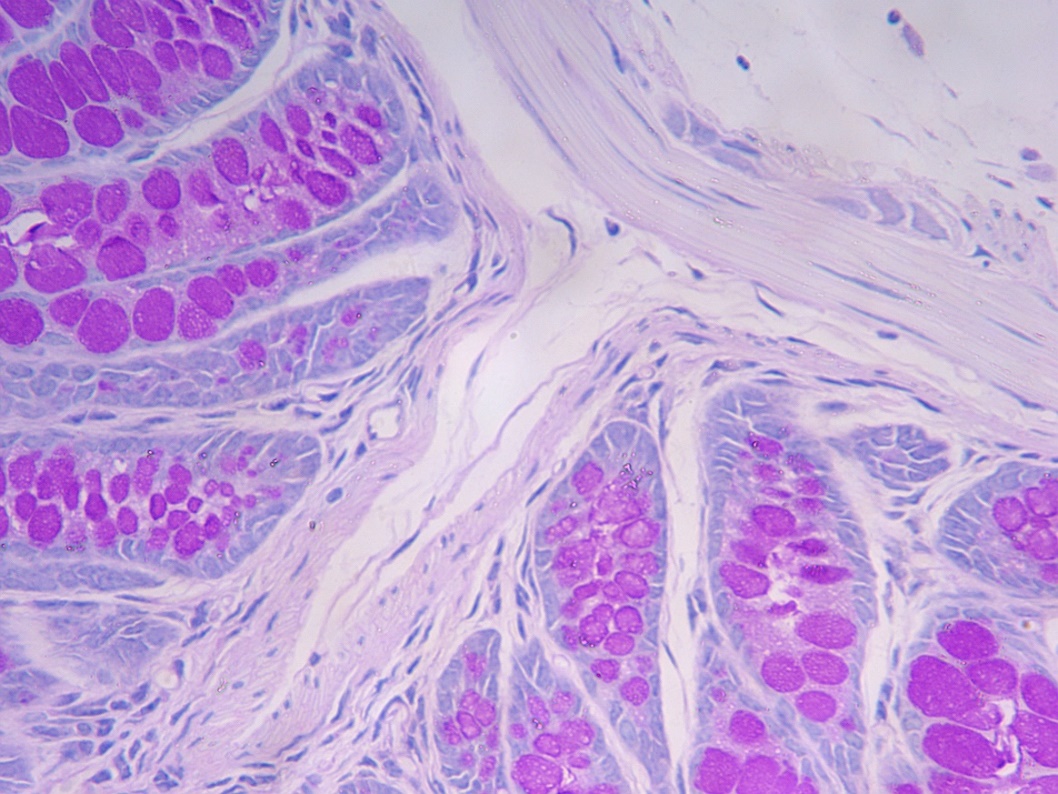


Figure 43 Periodic acid–Schiff staining of colon (ZSPL, ×200)


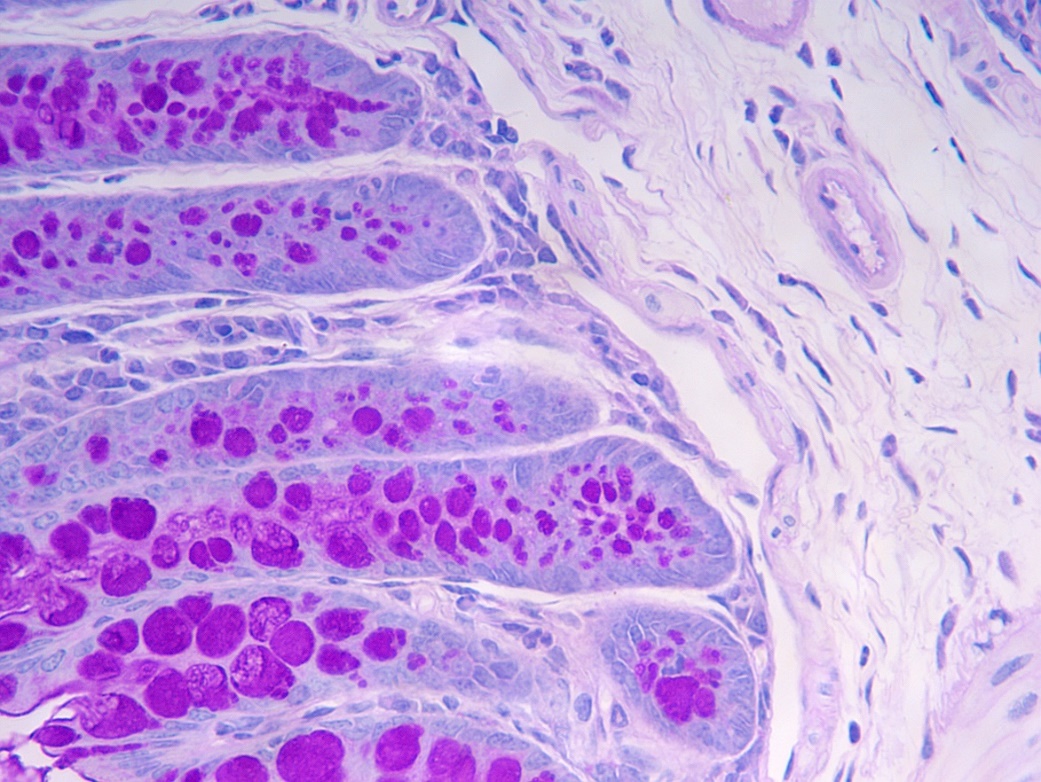


Figure 44 Periodic acid–Schiff staining of colon (ZSPH, ×200)
